# Single Cell Atlas: a single-cell multi-omics human cell encyclopedia

**DOI:** 10.1186/s13059-024-03246-2

**Published:** 2024-04-19

**Authors:** Lu Pan, Paolo Parini, Roman Tremmel, Joseph Loscalzo, Volker M. Lauschke, Bradley A. Maron, Paola Paci, Ingemar Ernberg, Nguan Soon Tan, Zehuan Liao, Weiyao Yin, Sundararaman Rengarajan, Xuexin Li, The SCA Consortium

**Affiliations:** 1 Institute of Environmental Medicine, Karolinska Institutet, 171 65 Solna, Sweden; 2 Cardio Metabolic Unit, Department of Medicine, and, Department of Laboratory Medicine , Karolinska Institutet, 141 86 Stockholm, Sweden; 3 Theme Inflammation and Ageing, Medicine Unit, Karolinska University Hospital, 141 86 Stockholm, Sweden; 4 Dr. Margarete Fischer-Bosch Institute of Clinical Pharmacology, 70376 Stuttgart, Germany; 5 University of Tuebingen, 72076 Tuebingen, Germany; 6 Department of Medicine, Brigham and Women’s Hospital, Harvard Medical School, Boston, MA 02115 USA; 7 Department of Physiology and Pharmacology, Karolinska Institutet, 171 65 Solna, Sweden; 8 Department of Computer, Control and Management Engineering, Sapienza University of Rome, 00185 Rome, Italy; 9 Department of Microbiology, Tumor and Cell Biology, Karolinska Institutet, 171 65 Solna, Sweden; 10 School of Biological Sciences, Nanyang Technological University, Singapore, 637551 Singapore; 11 Lee Kong Chian School of Medicine, Nanyang Technological University Singapore, Singapore, 308232 Singapore; 12 Department of Physical Therapy, Movement & Rehabilitation Sciences, Northeastern University, Boston, MA 02115 USA; 13 Department of General Surgery, The Fourth Affiliated Hospital, China Medical University, Shenyang, 110032 China; 14 Department of Medical Biochemistry and Biophysics, Karolinska Institutet, 171 65 Solna, Sweden

**Keywords:** Single-cell omics, Multi-omics, Single Cell Atlas, Human database, Single-cell RNA-sequencing, Spatial transcriptomics, Single-cell ATAC-sequencing, Single-cell immune profiling, Mass cytometry, Flow cytometry

## Abstract

Single-cell sequencing datasets are key in biology and medicine for unraveling insights into heterogeneous cell populations with unprecedented resolution. Here, we construct a single-cell multi-omics map of human tissues through in-depth characterizations of datasets from five single-cell omics, spatial transcriptomics, and two bulk omics across 125 healthy adult and fetal tissues. We construct its complement web-based platform, the Single Cell Atlas (SCA, www.singlecellatlas.org), to enable vast interactive data exploration of deep multi-omics signatures across human fetal and adult tissues. The atlas resources and database queries aspire to serve as a one-stop, comprehensive, and time-effective resource for various omics studies.

**Supplementary Information:**

The online version contains supplementary material available at 10.1186/s13059-024-03246-2.

## Background

The human body is a highly complex system with dynamic cellular infrastructures and networks of biological events. Thanks to the rapid evolution of single-cell technologies, we are now able to describe and quantify different aspects of single cellular activities using various omics techniques [1–4]. Observing or integrating multiple molecular layers of single cells has promoted profound discoveries in cellular mechanisms [5–8]. To accommodate the exponential growth of single-cell data [9, 10] and to provide comprehensive reference catalogs of human cells [11], many have dedicated to single-cell database or repository constructions [9, 11–15]. These databases vary in purpose and scope: some served as data repositories for raw/processed data retrieval [11, 12, 14]; quick references to cell type compositions and cellular molecular phenotypes across tissues [11, 16, 17]; summarized published study findings for global cellular queries across tissues or diseases [9, 13, 18]; or simply web-indexed published results [19]. The aim of these resources is to provide immediate information sharing among the scientific communities and real-time queries of diverse cellular phenotypes, which, in turn, to accelerate research progress and to provide additional research opportunities.

However, majority of these databases often provide simple cellular overviews or signature profiles largely based on single-cell RNA-sequencing (scRNA-seq) data confined to limited multi-omics landscape [9, 11, 13, 20]. The need for a database capable of conducting in-depth, real-time rapid queries of several single-cell omics at a time across almost all human tissues has not yet been met. This limitation has motivated us to build a one-stop single-cell multi-omics queryable database on top of constructing the multi-tissue and multi-omics human atlas.

Here, we present the Single Cell Atlas (SCA), a single-cell multi-omics map of human tissues, through a comprehensive characterization of molecular phenotypic variations across 125 healthy adult and fetal tissues and eight omics, including five single-cell (sc) omics modalities, i.e., scRNA-seq [21], scATAC-seq [22], scImmune profiling [23], mass cytometry (CyTOF) [24, 25], and flow cytometry [26, 27]; alongside spatial transcriptomics [28]; and two bulk omics, i.e., RNA-seq [29] and whole-genome sequencing (WGS) [30]. Prior to quality control (QC) filtering, we have collected 67,674,775 cells from scRNA-Seq, 1,607,924 cells from scATAC-Seq, 526,559 clonotypes from scImmune profiling, and 330,912 cells from multimodal scImmune profiling with scRNA-Seq, 95,021,025 cells from CyTOF, and 334,287,430 cells from flow cytometry; 13 tissues from spatial transcriptomics; and 17,382 samples from RNA-seq and 837 samples from WGS. We demonstrated through case studies the inter-/intra-tissue and cell-type variabilities in molecular phenotypes between adult and fetal tissues, immune repertoire variations across different T and B cell types in various tissues, and the interplay between multiple omics in adult and fetal colon tissues. We also exemplified the extensive effects of monocyte chemoattractant family ligands (i.e., the CCL family) [31] on interactions between fibroblasts and other cell types, which demonstrates its key regulatory role in immune cell recruitment for localized immunity [32, 33].

## Construction and content

### An overview of the multi-omics healthy human map

**Fig. 1:**
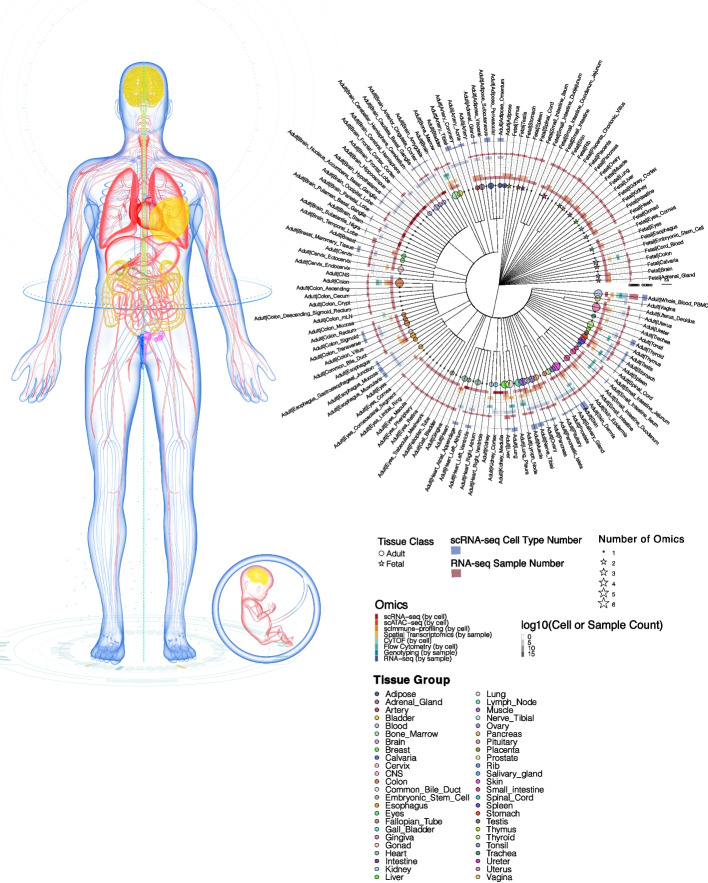
A multi-omics healthy human single-cell atlas. Circos plot depicting the tissues present in the atlas. Tissues belonging to the same organ were placed under the same cluster and marked with the same color. Circles and stars represent adult and fetal tissues, respectively. The size of a circle or a star indicates the number of its omics data sets present in the atlas. The intensity of the heatmap in the middle of the Circos plot represents the cell count for single-cell omics or the sample count for bulk omics. The bar plots on the outer surface of the Circos represent the number of cell types in the scRNA-seq tissues (in blue) or the number of samples in bulk RNA-seq tissues (in red)

We conducted integrative assessments of eight omics types from 125 adult and fetal tissues from published resources and constructed a comprehensive single-cell multi-omics healthy human map termed SCA (Fig. 1). Each tissue consisted of at least two omics types, with the colon having the full spectrum of omics layers, which allowed us to investigate extensively the key mechanisms in each molecular layer of colonic tissue. Organs and tissues with at least five omics layers included colon, blood (whole blood and PBMCs), skin, bone marrow, lung, lymph node, muscle, spleen, and uterus (Additional file 2: Table S1). Overall, the scRNA-seq data set contained the highest number of matching tissues between adult and fetal groups, which allowed us to study the developmental differences between their cell types. For scRNA-seq data, majority of the sample matrices retrieved from published studies have already undergone filtering to eliminate background noise, including low-quality cells which are most probable empty droplets. However, some samples downloaded retained their raw matrix form, which contained a significant amount of background noise. Consequently, before proceeding with any additional QC filtering, we standardized all scRNA-seq data inputs to the filtered matrix format, ensuring that all samples underwent the removal of background noise before further processing (Additional file 2: Table S2). This preprocessing step resulted in the removal of 61,774,307 cells out of the original 67,674,775 cells in the downloaded scRNA-seq dataset, leaving us with 5,900,468 cells for subsequent QC filtering. Strict QC was then carried out to filter debris, damaged cells, low-quality cells, and doublets for single-cell omics data [34], as well as low-quality samples for bulk omics data. After QC filtering, 3,881,472 high-quality cells were obtained for scRNA-Seq; 773,190 cells for scATAC-Seq; 209,708 cells for multimodal scImmune profiling with scRNA-seq data; 2,278,550 cells for CyTOF; and 192,925,633 cells for flow cytometry data. For scImmune profiling alone, clonotypes with missing CDR3 sequences and amino acid information were filtered, leaving 167,379 unique clonotypes across 21 tissues in the TCR repertoires and 16 tissues in the BCR repertoires. For RNA-seq and WGS, 163 severed autolysis samples were removed, leaving 16,704 samples for RNA-seq and 837 for genotyping data.

### Single-cell RNA-sequencing analysis of adult and fetal tissues revealed cell-type-specific developmental differences

**Fig. 2:**
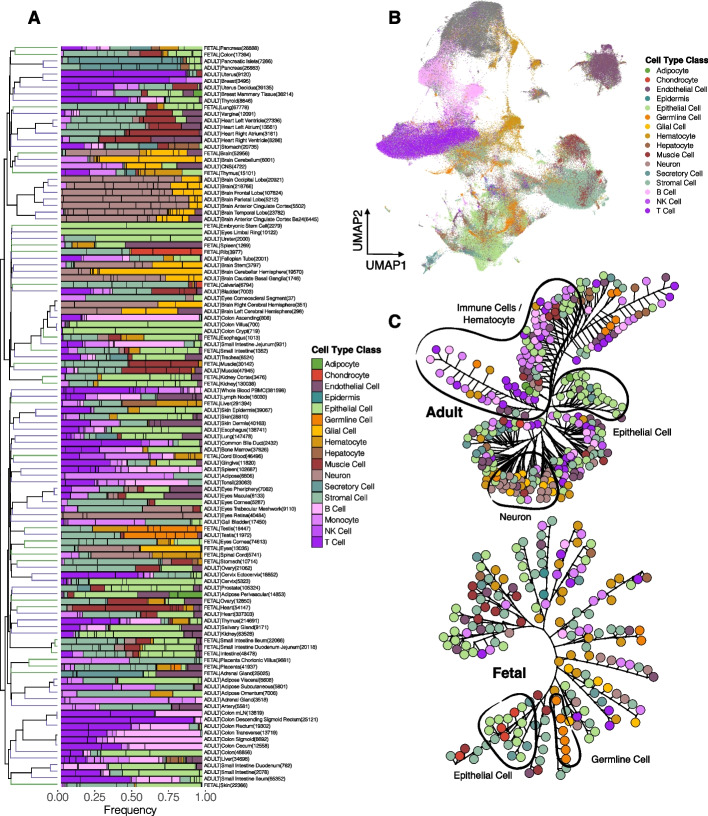
scRNA-seq integrative analysis revealed similarity and heterogeneity between adult and fetal tissues. **A** Clustering of the 417 cell types from scRNA-seq data, consisting of 92 tissues based on their cell type proportion within each tissue group. Cell types were colored based on the cell type class indicated in the legend. The numbers in the bracket represent the cell number within the tissue group. **B** UMAP of the cells present in the 94 adult and fetal tissues from scRNA-seq data, colored based on their cell type class. **C** Phylogenetic tree of the adult (left) and fetal (right) cell types. Clustering was performed based on their top regulated genes. The color represents the cell type class. Distinct clusters are outlined in black and labeled

**Fig. 3:**
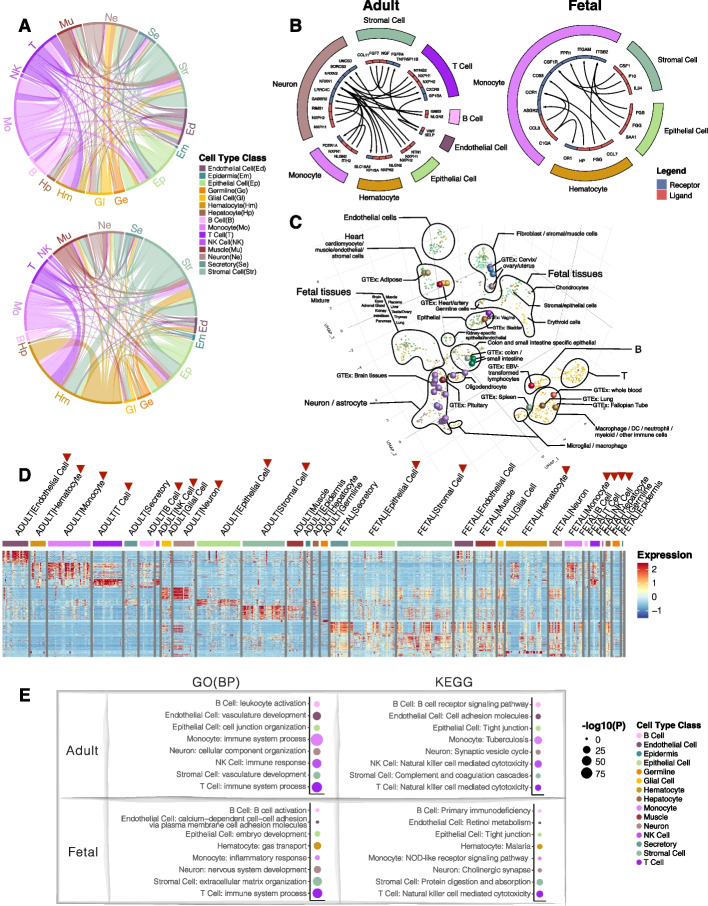
In-depth assessment of the integrated scRNA-seq further revealed inter-and intra-group similarities between adult and fetal tissues. **A** Chord diagrams of the highly correlated (AUROC > 0.9) adult and fetal cell types. Each connective line in the middle of the diagrams represents the correlation between two cell types. The color represents the cell type class. **B** Top receptor-ligand interactions between cell type classes in adult tissues (left) and fetal tissues (right). Color blocks on the outer circle represent the cell type class, and the color in the inner circle represents the receptor (blue) and ligand (red). Arrows indicate the direction of receptor-ligand interactions. **C** 3D tSNE of the integrative analysis between scRNA-seq and bulk RNA-seq tissues. The colors of the solid dots represent cell types in scRNA-seq data, and the colors of the spheres represent tissues of the bulk data. T indicates the T cell cluster, and B indicates the B cell cluster. **D** Heatmap showing the top DE genes in each cell type class of the adult and fetal tissues. Scaled expression values were used. Color blocks on the top of the heatmap represent cell type classes. Red arrows indicate the selected cell type classes for subsequent analyses. **E** Top significant GO BP and KEGG pathways for the cell type classes in adult and fetal tissues. The size of the dots represents the significance level. The color represents the cell type class

In total, out of the 125 adult and fetal tissues from all omics types, the scRNA-seq molecular layer in the SCA consisted of 92 adult and fetal tissues (Additional file 1: Fig. S1, Additional file 2: Additional file 2: Table S1), spanning almost all organs and tissues of the human body. We profiled all cells from scRNA-seq data and annotated 417 cell types at fine granularity, in which we categorized them into 17 major cell type classes (Fig. 2A). Comparing across tissues, most of them contained stromal cells, endothelial cells, monocytes, epithelial cells, and T cells (Fig. 2A). Comparing across the cell type classes, epithelial cells constituted the highest cell count proportions, followed by stromal cells, neurons, and immune cells (Fig. 2A). For adult tissues, most of the cells were epithelial cells, immune cells, and endothelial cells; whereas in fetal tissues, stromal cells, epithelial cells, and hematocytes constituted the largest cell type class proportions. Of these 92 tissues from the scRNA-seq data, we carried out integrative assessments of these tissues (Figs. 2 and 3) to study cellular heterogeneities in different developmental stages of the tissues.

For each cell type, we performed differential expression (DE) analysis for each tissue to obtain the DE gene (DEG) signature for each cell type. We assessed the global gene expression patterns between cell types across the tissues based on their upregulated genes (Additional file 2: Table S3) for adult and fetal tissues (Fig. 2C, Additional file 1: Fig. S2). In adult tissues, immune cells (i.e., B, T, monocytes, and NK cells) with hematocytes, stromal cells, neurons, endothelial cells, and epithelial cells formed distinct cellular clusters (Fig. 2C, Additional file 1: Fig. S2A), demonstrating highly similar DEG signatures within each of these cell type classes, consistent with the clustering patterns in the previous scRNA-seq atlas [35]. In fetal tissues, segregation is comparatively less distinctive such that only a subgroup of epithelial cells formed a distinct cell type cluster, cells from the immune cell type classes as well as hematocytes coalesced to form another cluster, and stromal cells formed small clusters between other fetal cell types (Fig. 2C, Additional file 1: Fig. S2B), which could represent the similarity in gene expression with other cell types during lineage commitment of stromal cell differentiation [36].

We next investigated the underlying gene regulatory network (GRN) of the transcriptional activities of cell types across adult and fetal tissues [37]. We identified active transcription factors (TFs) detected for cell types within each tissue (AUROC > 0.1), and based on these TF signatures, we measured similarities between cell types for adult and fetal tissues (Additional file 1: Fig. S3). For adult tissues, clustering patterns similar to Additional file 1: Fig. S1A were observed (Fig. 2C, Additional file 1: Fig. S3A). In fetal tissues, two unique clusters, including immune cells with hematocytes and stromal cells, were observed (Additional file 1: Fig. S3B). Higher similarity in transcription regulatory patterns of stromal cells was observed compared to their gene expression patterns. The concordance between gene expression and transcription regulatory patterns within adult and fetal tissues demonstrated a direct and uniform interplay between the two molecular activities. In terms of the varying TF and DEG clustering patterns between adult and fetal tissues, the adult cell types demonstrated more similar transcriptional activities within the cell type classes than the less-differentiated fetal cell types, which shared more common transcriptional activities.

We dissected the correlation pattern of the clusters shown in Fig. 2C by drawing inferences from their highly correlated (AUROC > 0.9) cell-type pairs (Fig. 3A). Specifically, for the immune cluster in adult tissues, monocytes accounted for most of the high correlations within the immune cell cluster, followed by T cells (Fig. 3A). For fetal tissues, a high number of correlations was observed between the immune cells (i.e., mostly monocytes and T cells) and hematocytes (Fig. 3A), which explained the clustering pattern observed in fetal tissues (Fig. 2C). For fetal stromal cells, other than with their own cell types, large coexpression patterns were observed with the hematocytes and the epithelial cells, and a smaller proportion of correlations with other clusters (Fig. 3A), which accounted for the small clusters of stromal cells formed between other cell types (Fig. 2C, Additional file 1: Fig. S2B).

To describe possible cellular networking between the cell type class clusters in Fig. 2C, we inferred cell–cell interactions [38] based on their gene expression (Additional file 2: Table S4), and variations between adult and fetal tissues were observed (Fig. 3B). In adult tissues, many cell type classes displayed interactions with the neurons, in which they networked with epithelial cells through UNC5D/NTN1 interaction; with stromal cells through SORCS3/NGF; with T cells through LRRC4C/NTNG2; etc. (Fig. 3B). Among the top interactions of fetal tissues, among the top interactions, monocytes actively network with other cells, such as via CCR1/CCL7 with hematocytes, CSF1R/CSF1 with stromal cells, and FPR1/SSA1 with epithelial cells.

We performed a pseudobulk integrative analysis of the cell types of the scRNA-seq data from 19 tissues found in both adult and fetal tissues, with the 54 tissues from the bulk RNA-seq data (Fig. 3C) to compare single-cell tissues with the corresponding tissues in the bulk datasets. For cell types of scRNA-seq data, adult cell types formed distinct clusters of T cells, B cells, hematocytes, stromal cells, epithelial cells, endothelial cells, and neurons (Fig. 3C). Fetal cell types, by comparison, formed a unique cluster of cell types separating themselves from adult cell types. Internally, a gradient of cell types from brain tissues to cell types from the digestive system was observed in this fetal cluster. Fusing the bulk tissue-specific RNA-seq data sets with the pseudobulk scRNA-seq cell types gave close proximities of the bulk brain tissues with the pseudobulk brain-specific cell types, such as neurons and astrocytes (Fig. 3C). Bulk whole blood clustered with pseudobulk hematocytes, and bulk EBV-transformed lymphocytes clustered with pseudobulk B cells. Other distinctive clusters included bulk colon and small intestine clustered with pseudobulk colon- and small intestine-specific epithelial cells, and bulk heart clustered with pseudobulk cardiomyocytes and other muscle cells (Fig. 3C).

Next, we conducted gene ontology (GO) of biological processes (BPs) and KEGG pathway analyses [39–42] of the top upregulated genes of each cell type class cluster (Fig. 3D) found in Fig. 2C. Multiple testing correction for each cell type class was performed using Benjamini & Hochberg (BH) false discovery rate (FDR) [43]. At 5% FDR and average log2-fold-change > 0.25 (ranked by decreasing fold-change), the top three most significant genes of the remaining cell type classes were each scanned through the phenotypic traits from 442 genome-wide association studies (GWAS) and the UK Biobank [44, 45] to seek significant genotypic associations of the top genes with diseases and traits. Notably, for GO pathways, the most significant BPs for B and T cells in both adult and fetal tissues were similar (Fig. 3E). In contrast, epithelial cells and neurons differ in their associated BPs between adult and fetal tissues. For KEGG pathways, adult and fetal tissues shared common top pathways in T cells and in epithelial cells (Fig. 3E). Among the top genotype–phenotype association results of the top genes (Additional file 1: Fig. S4), SNP rs2239805 in HLA-DRA of adult monocytes has a high-risk association with primary biliary cholangitis, which is consistent with previous studies showing associations of HLA-DRA or monocytes with the disease [46–50].

### Multimodal analysis of scImmune profiling with scRNA-sequencing in multiple tissues

**Fig. 4:**
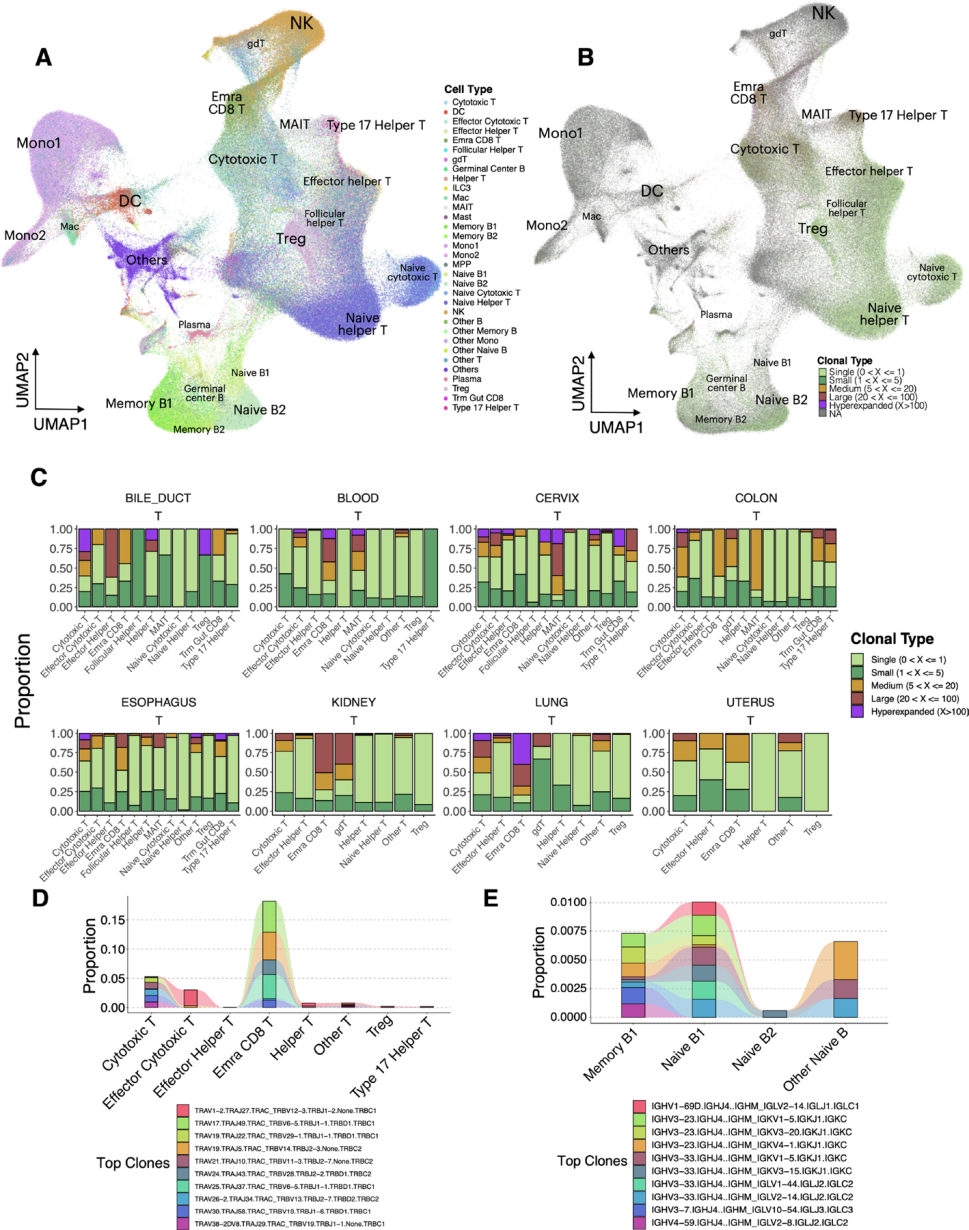
Multi-modal analysis of scImmune profiling with scRNA-seq revealed a clonotype expansion landscape in six tissues. **A** tSNE of cell types from the multi-modal tissues of the scImmune-profiling data. Colors represent cell types. Cell clusters were outlined and labeled. **B** tSNE of cell types from the multi-modal tissues of the scImmune-profiling data. Colors indicate clonal-type expansion groups of the cells. Cells not present in the T or B repertoires are shown in gray (NA group). **C** Stacked bar plots revealing the clonal expansion landscapes of the T and B cell repertoires across 6 tissues. Colors represent clonal type groups. **D** Alluvial plot showing the top clonal types in T cell repertoires and their proportions shared across the cell types. Colors represent clonotypes. **E** Alluvial plot showing the top clonal types in B cell repertoires and their proportions shared across the cell types. Colors represent clonotypes

To decipher the immune landscape at the cell type level in the scImmune profiling data, we carried out an integrative in-depth analysis of the immune repertoires with their corresponding scRNA-seq data. The overall landscape of the cell types mainly included clusters of naïve and memory B cells, naïve T/helper T/cytotoxic T cells, NK cells, monocytes, and dendritic cells (Fig. 4A) and mainly comprised immune repertoires from the blood, cervix, colon, esophagus, and lung (Additional file 1: Fig. S5). On a global scale, we examined clonal expansions [51, 52] in both T and B cells across all tissues. Here, we defined unique clonal types as unique combinations of VDJ genes of the T cell receptor (TCR) chains (i.e., alpha and beta chains) and immunoglobin (Ig) chains on T cells and B cells, respectively. Integrating clonal type information from both the T and B cell repertoires with their scRNA-seq revealed sites of differential clonal expansion in various cell types (Fig. 4B and C, Additional file 1: Fig. S5). In T cell repertoires, high proportions of large or hyperexpanded clones were found in terminally differentiated effector memory cells reexpressing CD45RA (Temra) CD8 T cells [53, 54] and cytotoxic T cells, and a large proportion of them was found in the lung (Fig. 4C, Additional file 1: Fig. S5), which interplays with the highly immune regulatory environment of the lungs to defend against pathogen or microbiota infections [55, 56]. MAIT cells [57, 58] have also demonstrated their large or high expansions across tissues, especially in the blood, colon, and cervix (Additional file 1: Fig. S5A), with their main function to protect the host from microbial infections and to maintain mucosal barrier integrity [58, 59]. In contrast, single clones were present mostly in naïve helper T cells and naïve cytotoxic T cells. (Additional file 1: Fig. S5B) and were almost homogeneously across tissues (Fig. 4C). This observation ensures the availability of high TCR diversity to trigger sufficient immune response for new pathogens [60]. For the B cell repertoire in blood, most of these immunocytes remained as single clones or small clones, with a small subset of naïve B cells and memory B cells exhibiting medium clonal expansion (Additional file 1: Fig. S5B).

Among the top clones (Fig. 4D), TRAV17.TRAJ49.TRAC_TRBV6-5.TRBJ1-1.TRBD1.TRBC1 was present mostly in Temra CD8 T cells and shared the same clonal type sequence with cytotoxic T and helper T cells (Additional file 2: Table S5). This top clone was found to be highly represented in the lung, and comparatively, other large clones of CD8 T cells were found in the blood (Additional file 1: Fig. S5C). The top ten clones were found in Temra CD8 T cells of blood and lung tissues and cytotoxic T cells and helper T cells from blood, cervix, and lung tissues (Additional file 1: Fig. S5C). Some of them exhibited a high prevalence of cell proportions in Temra CD8 T cells (Fig. 4D). In the B cell repertoire of blood, the top clones were found only in naïve and memory B cells, with similar proportions for each of the top clones (Fig. 4E).

### Multi-omics analysis of colon tissues across five omics data sets

**Fig. 5:**
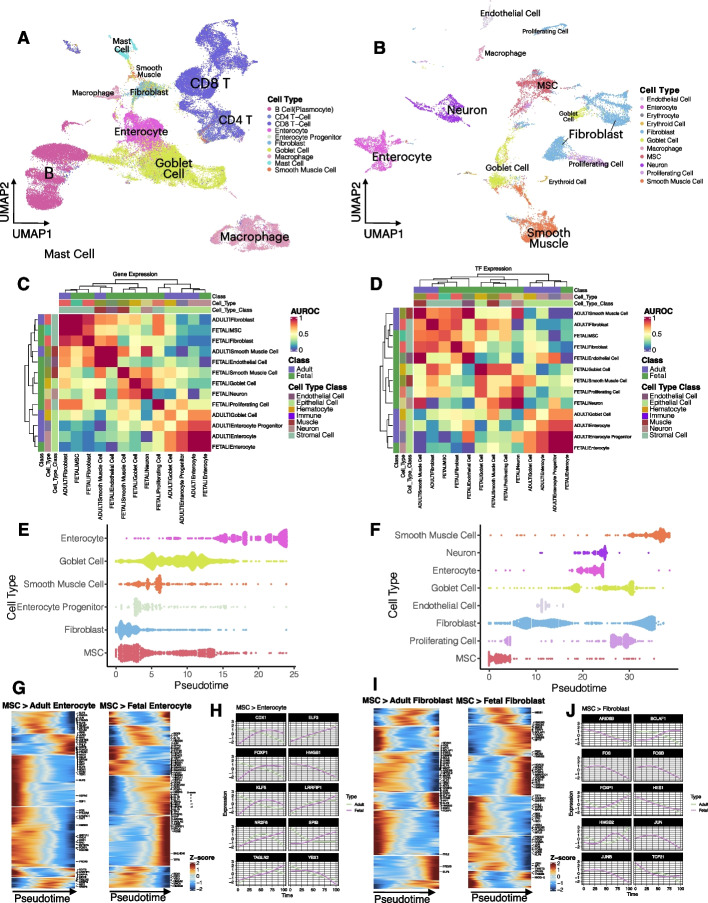
In-depth scRNA-seq analysis revealed distinct variations between adult and fetal colons. **A** tSNE of the adult colon; colors represent cell types. **B** tSNE of the fetal colon; colors represent cell types. **C** Heatmap showing the correlations of the cell types of the MSC lineage from adult and fetal colons based on their top upregulated genes. The intensity of the heatmap shows the AUROC level between cell types. Color blocks on the top of the heatmap represent classes (first row from the top), cell types (second row), and cell type classes (third row). **D** Heatmap showing the correlations of the cell types of the MSC lineage from adult and fetal colons based on the expression of the TFs. The intensity of the heatmap shows the AUROC level between cell types. Color blocks on the top of the heatmap represent classes (first row from the top), cell types (second row), and cell type classes (third row). **E** Pseudotime trajectory of the MSC lineage in the adult colon. The color represents the cell type, and the violin plots represent the density of cells across pseudo-time. **F** Pseudo-time trajectory of the MSC lineage in the fetal colon. The color represents the cell type, and the violin plots represent the density of cells across pseudotime. **G** Heatmap showing the pseudotemporal expression patterns of TFs in the lineage transition of MSCs to enterocytes in both adult and fetal colons. Intensity represents scaled expression data. The top 25 TFs for MSCs or their differentiated cells are labeled. **H** Pseudotemporal expression transitions of the top TFs in the MSC-to-enterocyte transitions for both adult and fetal colons. **I** Heatmap showing the pseudotemporal expression patterns of TFs in the lineage transition of MSCs to fibroblasts in both adult and fetal colons. Intensity represents scaled expression data. The top 25 TFs for MSCs or their differentiated cells are labeled. **J** Pseudotemporal expression transitions of the top TFs in the MSC-to-fibroblast transitions for both adult and fetal colons

To examine the phenotypic landscapes and interplays between different omics methods and data sets, we carried out an interrogative analysis of colon tissue across five omics data sets, including scRNA-Seq, scATAC-Seq, spatial transcriptomics, RNA-seq, and WGS, to examine the phenotypic landscapes across omics layers and the interplays and transitions between omics layers. In the overview of the transcriptome landscapes in adult and fetal colons (Fig. 5A and B), the adult colon consisted of a large proportion of immune cells (such as B cells, T cells, and macrophages) and epithelial cells (such as mucin-secreting goblet cells and enterocytes) (Fig. 5A). In contrast, the fetal colon contained a substantial number (proportion) of mesenchymal stem cells (MSCs), fibroblasts, smooth muscle cells, neurons, and enterocytes and a very small proportion of immune cells (Fig. 5B).

As there were fewer immune cells observed in the fetal colon as compared to the adult colon, we compared the MSC lineage cell types between the two groups. Based on their differential gene expression signatures (Fig. 5C) and their TF expression (Fig. 5D), the highly specialized columnar epithelial cells, enterocytes, for both molecular layers correlated well between adult and fetal colons, unlike other cell types, which did not demonstrate high correlations between their adult and fetal cells. Other than the enterocytes, adult and fetal fibroblasts were highly similar to MSCs in both transcriptomic and regulatory patterns (Fig. 5C and D). We modeled pseudo-temporal transitions of MSC lineage cells, and similar phenomena were observed (Fig. 5E and F). Both adult and fetal fibroblasts were pseudotemporally closer to MSCs, and the transitions were much earlier than other cells. Analysis across regulatory, gene expression, and pseudotemporal patterns showed in both adult and fetal colons that fibroblasts were more similar to MSCs phenotypically, as shown in prior literature reports [61–63] and recently with therapeutic implications [64, 65]. In addition, transient phases of cells along the MSC lineage trajectory were observed for enterocytes and goblet cells (Fig. 5E and F), which demonstrated that these high plasticity cells were at different cell-state transitions before their full maturation, as evident in the literature [66, 67]. By contrast, the fetal intestine was more primitive than the adult intestine during fetal development, and as a key cell type in extracellular matrix (ECM) construction [68], fibroblasts displayed transitional cell stages of cells along the pseudotime trajectory (Fig. 5F).

Comparing regulatory elements of these transitions demonstrated similarities and differences (Fig. 5G–J, Additional file 1: Fig. S6). For MSC-to-enterocyte transitions (Fig. 5G, Additional file 2: Table S6), the leading TFs with significant pseudotemporal changes were labeled. The expression E74 Like ETS transcription factor 3, ELF3, which belongs to the epithelium-specific ETS (ESE) subfamily [69], increased during the transition for both adult and fetal enterocytes (Fig. 5H, Additional file 2: Table S6) and as previously demonstrated is important in intestinal epithelial differentiation during embryonic development in mice [69, 70]. Conversely, high mobility group box 1, HMGB1 [71], decreased pseudotemporally for both adult and fetal enterocytes (Fig. 5H, Additional file 2: Table S6) and has been shown to inhibit enterocyte migration [72]. The nuclear orphan receptor, NR2F6, a non-redundant negative regulator of adaptive immunity, [73, 74], displayed a comparative decline in expression halfway through the pseudotime transition for adult enterocytes but continued to increase for fetal enterocytes (Fig. 5H, Additional file 2: Table S6). Another TF from the ETS family, Spi-B transcription factor, SPIB, also showed differential expression during the transition between adult and fetal enterocytes (Fig. 5H), which was up-regulated in fetal enterocytes and down-regulated in adult enterocytes, suggesting its potential bi-functional role in enterocyte differentiation in fetal-to-adult transition.

For MSC-to-fibroblast transitions (Fig. 5I, Additional file 2: Table S6), TFs such as ARID5B, FOS, FOSB, JUN, and JUNB displayed almost identical trajectory patterns between adult and fetal fibroblasts (Fig. 5J, Additional file 2: Table S6). Of these TFs, FOS, FOSB, JUN, and JUNB were shown to be absent in the healthy mucosa transcriptional networks [75], in line with their observations in Fig. 5J. By contrast, Bcl-2-associated transcription factor 1, BCLAF1, was pseudotemporally up-regulated in fetal fibroblasts but downregulated in adult fibroblasts. Prior studies showed that knocking out BCLAF1 is embryonic lethal [76, 77] and yet could be oncogenic in colon cancer [78], which could explain the trajectory difference of it in fetal and adult. Other cell types also displayed varying degrees of similarities and differences (Additional file 1: Fig. S5, Additional file 2: Table S6).

**Fig. 6:**
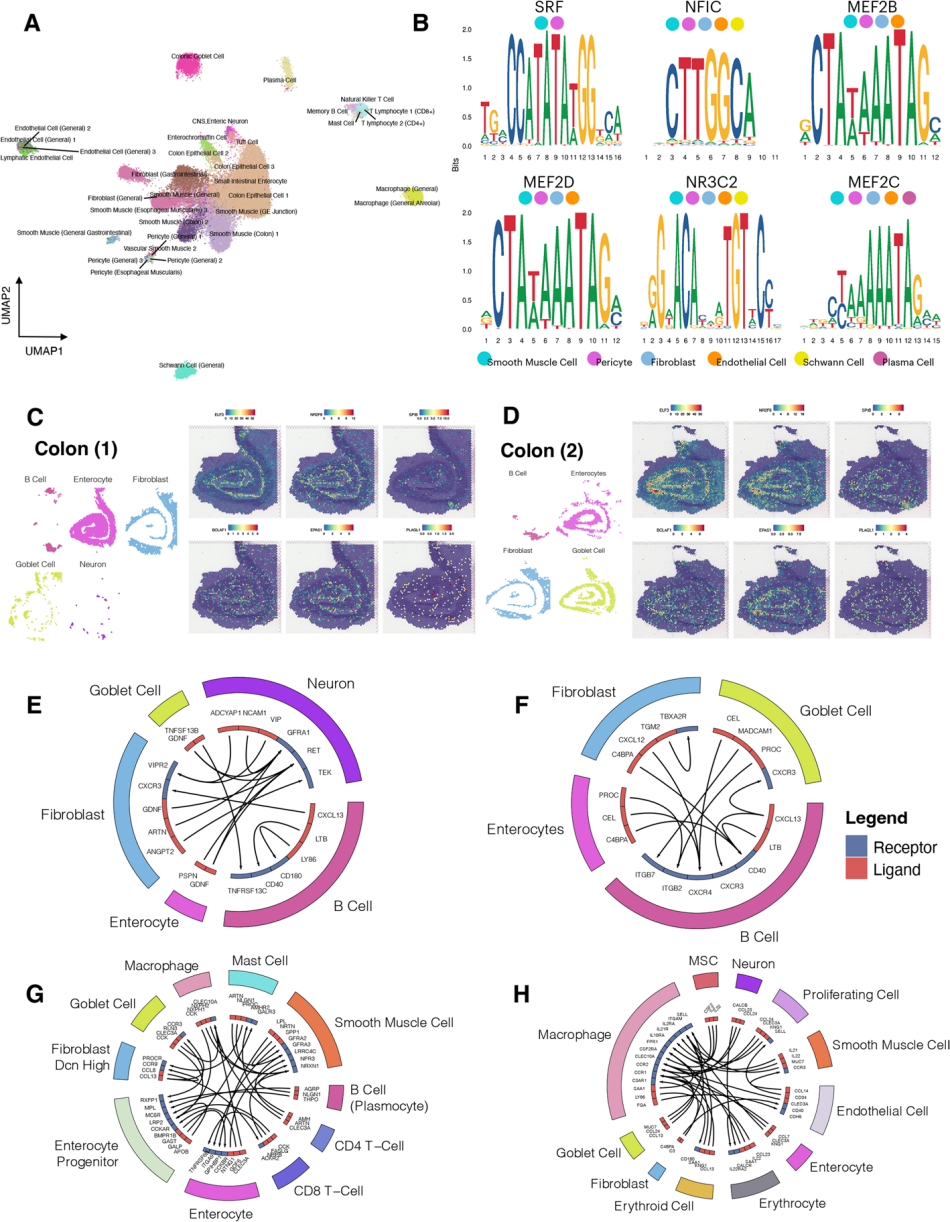
Multi-omics analysis of adult and fetal colon tissues revealed distinct variations between adults and fetuses as well as across omics. **A** UMAP of cell types present in the scATAC-Seq of the adult colon. Colors represent cell types. **B** Top enriched motif sequences in cell types of the adult colon scATAC-Seq data. **C**,**D** Spatial transcriptomic profiles of adult colon sample 1 (**C**) and sample 2 (**D**). The top TFs were selected, and their spatial expressions were mapped onto the slide images. **E**,**F** Top receptor-ligand interactions between cell type classes in colon 1 (**E**) and colon 2 (**F**) of the spatial transcriptomics data. Color blocks on the outer circle represent the cell type class, and the color in the inner circle represents the receptor (blue) and ligand (red). Arrows indicate the direction of receptor-ligand interactions. **G**,**H** Top receptor-ligand interactions between cell type classes in the adult colon (**G**) and fetal colon (**H**) of the scRNA-seq data. Color blocks on the outer circle represent the cell type class, and the color in the inner circle represents the receptor (blue) and ligand (red). Arrows indicate the direction of receptor-ligand interactions

In scATAC-Sequencing, we examined the contributions of *cis*-regulatory elements in the adult colon. We identified DA peaks for cell clusters and identified corresponding genes closest to these DA peak regions. Cell type identities were postulated based on the gene activities of the scATAC-Seq data (GSEA) [79, 80] (Fig. 6A). Common cell types were detected in scATAC-Seq compared to scRNA-seq (Figs. 5A and 6A). We performed sequence motif analysis to detect regulatory sequences unique to each cell type based on their leading DA peaks; among the top enriched motifs, many of the Myocyte Enhancer Factors such as MEF2B, MEF2C, and MEF2D from cells such as smooth muscle cells and pericytes, were found to be significantly enriched (Fig. 6B), which were also up-regulated in the scRNASeq findings shown earlier (Additional file 2: Table S6).

We examined the physical landscape of the leading TFs (found in scRNA-Seq and scATAC-Seq) in spatial transcriptomics data from two adult colons [5]. TFs ELF3 and NR2F6 were expressed generally in many locations in colonic tissue and displayed similar expression patterns for both of the adult colons (Fig. 6C and D), consistent with significant up-regulation in almost all MSC lineage cell types in the pseudotemporal transitions (Additional file 2: Table S6). In contrast, SPIB was not up-regulated in general, while displaying higher expression in B cells (Fig. 6C and D), consistent with its role in adaptive immunity, as previously discussed. For other leading TFs, such as BCLAF1, EPAS1, and PLAG1, there were no clear discrete patterns of expression among the cell types.

To examine how cells interact with one another in spatial transcriptomics of the adult colon, we performed receptor-ligand interaction analysis [38]. Leading interactions included VIP/VIPR2 and ADCYAP1/VIPR2 interactions between neurons and fibroblasts, the NCAM1/GFRA1 interaction between neuronal cells, as well as LTB/CD40 and LY86/CD180 interactions between B cells (Fig. 6E, Additional file 2: Table S7). In colon 2, leading interactions occurred between the B cells and between the B cells and enterocytes or fibroblasts. These included LTB/CD40, APOE/LRP8, LY86/CD180, and VCAM1/ITGB7 between B cells; APOE/VLDLR between B cells (APOE) and enterocytes (VLDLR); and CXCL12/CXCR4, FN1/CD79A, CD34/SELL, and ICAM2/ITGAL between fibroblasts and B cells (Fig. 6F, Additional file 2: Table S7).

The same type of analysis was performed on both scRNA-seq from both adult and fetal colons. In the adult colon in scRNA-seq (Fig. 6G), the fibroblasts comprised the leading interactions with cells such as CD8 T cells (CCL8-ACKR2), with (other) fibroblasts (CCL13-CCR9), goblet cells (CCL13-CCR3), and mast cells (PROC-PROCR). In the fetal colon, leading interaction pairs were derived mostly from fibroblasts and macrophages with other cells (Fig. 6H, Additional file 2: Table S7), including C4BPA-CD40 between fibroblasts (C4BPA) and endothelial cells (CD40); CCL24-CCR2 between neuronal cells (CCL24) and macrophages (CCR2); CCL13-CCR1 and MUC7-SELL between goblet cells (CCL13 and MUC7) and macrophages (CCR1 and SELL); and IL21-IL21R between smooth muscle cells (IL21) and macrophages (IL21R). In scRNA-seq of both adult and fetal colons, the active interactions of fibroblasts with other cells based on CCL family ligand-receptor interactions seemed to suggest its key regulatory role in immune cell recruitment in the colon (via the active interaction and activation of monocyte chemoattractants, i.e., the CCL family), consistent with prior publications [32, 33].

Comparing the two omics data sets, both colon samples from spatial transcriptomics data shared leading interactions with that of the scRNA-seq from adult and fetal colons (Additional file 2: Table S7). Between spatial colon 1 and the scRNA-seq fetal colon, common interaction pairs were found between neuronal cells, enterocytes with neurons, and neurons with fibroblasts (Additional file 2: Table S7). For spatial colon 2, 25 of its 95 top unique interactions were shared with the scRNA-seq adult colon, and 10 were shared with the scRNA-seq fetal colon (Additional file 2: Table S7). For the scRNA-seq adult colon, 445 of its 852 top unique interactions were found in the scRNA-seq fetal colon. For example, CLEC3A-CLEC10A interactions between macrophages (CLEC10A) and enterocytes (CLEC3A), goblet cells (CLEC3A), or smooth muscle cells (CLEC3A), as well as between macrophages. Among them, the scRNA-seq fetal colon seemed to share the greatest number of cell-type-specific interactions with the other three groups (Additional file 2: Table S7).

At 1% BH FDR and log2FC > 0.25 for the bulk RNA-seq data in adult transverse colon data, we compared these upregulated genes with the top genes in scRNA-seq and the top genes in expression quantitative trait loci (eQTL) (eGenes) and splicing QTL (sQTL) (sGenes) of WGS of the corresponding transverse colon data (Additional file 1: Fig. S6). Comparing the top 10 genes of eGenes and sGenes, no common genes were found (Additional file 1: Figs. S7A and S7B). Comparing the overlapping patterns in bulk transcriptomics with scRNA-seq data, there was a much higher number of overlaps in scRNA-seq with eGenes and sGenes compared to bulk RNA-seq (Additional file 1: Fig. S7C). We grouped the overlapping genes according to their cell types in scRNA-seq (Additional file 1: Fig. S7D). In particular, the goblet cells and enterocytes in eGenes were similar in proportion within eGenes for bulk RNA-seq compared to scRNA-Seq. Similar phenomena were observed in sGenes (Additional file 1: Fig. S7D).

## Utility and discussion

### User interface (UI) overview

SCA offers an intuitive, user-friendly interface designed to facilitate seamless navigation and efficient phenotype retrieval by researchers across eight single-cell and bulk omics from 125 healthy adult and fetal tissues. Designed with a focus on user experience, the UI offers intuitive and simple navigations for users to explore complex layers of multi-omics multi-tissue resources. Here is an overview of the SCA UI, (I) Home Page: Landing page of the database to serve as the gateway to the comprehensive features of the SCA, offering users a starting point to dive into the wealth of multi-omics data. (II) About: This section offers a thorough description of the portal, complemented by an introductory video summarizing the key features of the database to provide guidance to new users. (II) Overview: Here, we highlight the diversity of omics data available, providing a snapshot of the various omics types and summarizing key information about each. (IV) Atlas: Features interactive representations of human adult and fetal anatomies, and a gateway for users to explore each tissue in-depth with detailed phenotypes specific to each tissue and their corresponding omics. (V) Query: While the Atlas tab is to showcase comprehensive features in each tissue, the Query tab is dedicated to exploring key phenotypic features across all tissues for different omics types, such as regulon search, receptor-ligand interactions, and clonotype abundance, etc. (VI) Demo: Offers a comprehensive walkthrough of the database, using the adult colon transverse tissue as an illustrative example, to demonstrate the capability of the platform and how users can extract meaningful insights. (VII) Analyze: Provides an extensive suite of tools tailored to assist users in performing single-cell analyses across a wide array of omics, along with rapid plotting tools that allow for the creation of customizable plots quickly and efficiently. (VIII) Download: Provides the option for batch downloads, enabling users to conveniently download the data utilized within the database based on their specific selections. (IX) Sources: Offers detailed information about the origins of the raw data used to construct the database, ensuring transparency and trust in the data provided. (X) Discussion: Facilitates a collaborative community space where users can interact, offer assistance, pose questions, and share feedback and suggestions, enhancing the collective utility of the platform. (XI) News: Keeps users informed about the latest updates, additions, and enhancements to the database, ensuring the SCA community stays abreast of new developments.

### Intended uses of the database and envisioned benefits

SCA is crafted to serve as a comprehensive resource in the burgeoning field of single-cell and multi-omics research. Its primary intention is to facilitate a deeper understanding of the cellular complexity and diversity inherent in healthy adult and fetal tissues through simultaneous exploration of multiple omics. Beyond this, SCA aims to serve as a robust analysis platform to support post-quantification analysis of high-throughput single-cell sequencing data. As such, researchers can leverage SCA for comparative studies, hypothesis generation, and validation purposes. The integration of multi-omics data facilitates a deeper understanding of cellular mechanisms, potentially accelerating discoveries in cellular mechanisms, developmental biology, and potential therapeutic targets.

Explicitly, SCA enables scientists to quickly derive insights that would otherwise require extensive time and resources to uncover, thereby speeding up the cycle of hypothesis, experimentation, and conclusion. The database will significantly enhance data accessibility and integration, allowing researchers to easily combine data from different omics types and tissues to obtain a holistic view of cellular functions. This integrative approach is crucial for understanding complex biological systems and for the development of comprehensive models of human health and disease. By cataloging cellular characteristics across a range of tissues and conditions, SCA empowers precision medicine initiatives. It provides a detailed cellular context for phenotypic variations and potential markers at the single-cell level and with bulk level for comparative assessments, supporting the development of potential personalized treatment plans based on cellular profiles.

SCA fosters a collaborative research environment by providing a common platform for scientists from diverse backgrounds with research specialties across tissues, diseases, and omics analysis. It encourages interdisciplinary approaches, connecting researchers from diverse fields and promoting the exchange of knowledge and methodologies. This collaborative ethos is expected to drive forward innovations in research and technology.

### Benchmarking with existing databases

Here, we evaluated our SCA database against other existing databases [9, 11, 13, 20, 81], emphasizing the distinctive attributes that make SCA stand out (Additional file 2: Table S8). SCA integrates eight distinct omics types, surpassing the scope of Single Cell Portal (SCP) [20], Human Cell Atlas (HCA) [11], GTEx Portal [81], DISCO [9], and Panglaodb [13] in providing a wide-ranging multi-omics platform for exhaustive single-cell omics research. Data accessibility is publicly available for all these platforms, except that GTEx Portal encompassing both public and protected datasets (Additional file 2: Table S8). SCA is noteworthy for its extensive coverage of eight single-cell and bulk omics over 125 differentiated tissues, established a significant lead over the other portals in terms of omics types. Furthermore, SCA sets a new standard with its unmatched capabilities. Other than the typical representations of cell type proportions and visualizing basic features in cell types, features that are notably limited or absent in SCP, HCA, DISCO, and Panglaodb, such as cell–cell interactions, transcription factor activities, the visualization of regulon modules, motif enrichments, clonotype abundance, detailed repertoire profiles, etc., are areas unaddressed by other databases. SCA is the sole provider of specialized queries targeting various phenotypes across multiple omics (Additional file 2: Table S8). This specificity of analysis remains unparalleled when juxtaposed with other databases in our comparative cohort. Ultimately, SCA stands out as a premier, all-encompassing resource for the omics research community.

### Future development and maintenance

In an effort to ensure the platform remains relevant, up-to-date, and increasingly valuable to the broad spectrum of researchers, we will be implementing annual updates. These will incorporate findings from newly published studies and novel phenotypic analyses gathered over the year. As we strive to continually enrich our platform, these updates will address gaps in tissue representation for each omics type, and simultaneously expand the sample size within each tissue. Our commitment to transparency and traceability is reflected in our approach to versioning. We will systematically denote improvements to the database, including new features and datasets, in an accessible point-form format. Updates will be marked by adjustments to the database accession number, with the current version designated as SCA V1.0.0. In addition to serving as a resource for data and phenotypic features, our ultimate aim is for SCA to function as a user-friendly platform, facilitating rapid access to multi-omics data resources and enabling cross-comparison of user datasets with our own.

## Conclusions

Our study establishes a comprehensive evaluation of the healthy human multi-tissue and multi-omics landscape at the single-cell level, culminating in the construction of a multi-omics human map and its accompanying web-based platform SCA. This innovative platform streamlines the delivery of multi-omics insights, potentially reducing costs and accelerating research by obviating the need for extensive data consolidation. The big data framework of SCA facilitates the exploration of a broad spectrum of phenotypic features, offering a more representative snapshot of the study population than traditional single omics or bulk analysis could achieve. This multi-omics approach is poised to be instrumental in unraveling the complexities of multidimensional biological systems, offering a holistic perspective that enhances our understanding of biological phenomena.

Despite its robust capabilities, SCA faces challenges associated with the technological limitations of flow cytometry and CyTOF modalities, which restrict the number of detectable proteins. These constraints complicate the integration of data from different studies. We have consciously chosen not to pursue the imputation of expression values across these datasets due to concerns about reliability. Moving forward, we aim to refine tissue stratification within the portal by introducing more detailed sample classifications, such as sampling sites, age groups, genders across tissues, and for fetal tissues, different developmental stages. This advancement depends on the acquisition of comprehensive data to support more precise and accurate analyses.

SCA is designed not only as a database but as a catalyst for a paradigm shift towards a multi-omics-focused research approach. It encourages the scientific community to embrace a multi-omics perspective in their research, facilitating the generation of new hypotheses and the discovery of novel insights. This platform is expected to foster an environment rich in intellectual exploration, propelling forward the development of groundbreaking research trajectories. In essence, SCA emerges as a pioneering open-access, single-cell multi-omics atlas, offering an in-depth view of healthy human tissues across a wide array of omics disciplines and 125 diverse adult and fetal tissues. It unlocks new avenues for exploration in multi-omics research, positioning itself as a vital tool in advancing our understanding of life sciences. SCA is set to become an invaluable asset in the research community, significantly contributing to advancements in biology and medicine by facilitating a deeper comprehension of complex biological systems.

### Supplementary Information

**Additional file 1:Figure S1.** Sample count in fetal and adult groups across tissues and omics types. **Figure S2.** Correlations between cell types based on gene expression signatures revealed distinct cell type class clusters. (A-B) Heatmap showing the correlations of the cell types from adult (A) and fetal (B) cell types based on the expression of their top upregulated genes. The intensity of the heatmap shows the AUROC level between cell types. Colour blocks on the top of the heatmap represent tissues (first row from the top), biological systems (second row), cell types (third row) and cell type classes (fourth row). **Figure S3.** Correlations between cell types based on TF signatures revealed similar clustering patterns. (A-B) Heatmap showing the correlations of the cell types from adult (A) and fetal (B) cell types based on the expression of the TF signatures of each cell type. The intensity of the heatmap shows the AUROC level between cell types. Colour blocks on the top of the heatmap represent tissues (first row from the top), biological systems (second row), cell types (third row) and cell type classes (fourth row). **Figure S4.** Phenotype or disease trait associations. Forest plot showing the associations of phenotype or disease traits in selected cell type classes of scRNA-seq data for both adult and fetal tissues. The X-axis displays the odds ratio of each trait, and the colors of the points represent cell type classes. **Figure S5.** Landscape of clonal expansion patterns across tissues. (A) tSNE of the tissues from the multi-modal tissues of the scImmune-profiling data. Colors indicate clonal type expansion groups of the cells. Cells not present in the T or B repertoires are colored gray (NA group). Tissues with too few cells present in the T or B repertoires were filtered (i.e., bile duct and kidney) in the main analysis. (B) Stacked bar plots revealing the overall clonal expansion landscapes of the T and B cell repertoires. Colors represent clonal type groups. (C) Alluvial plot showing the top clonal types in T cell repertoires and their proportions shared across tissues containing these clonotypes. Colors represent clonotypes. **Figure S6.** Pseudotime heatmaps of MSC lineage cell types in the adult and fetal colon. (A-B) Pseudotime trajectory of each cell type in the MSC lineage of adult (A) and fetal (B) colons. The color represents the cell type, and the violin plots represent the density of cells across pseudotime. **Figure S7.** Comparison of DE gene overlaps between bulk RNA-seq, scRNA-seq and WGS. (A) Chromosomal positions of the top 10 eGenes in colon transverse bulk RNA-seq data. Gene names and their SNP rsid are shown. (B) Chromosomal positions of the top 10 sGenes in colon transverse bulk RNA-seq data. Gene names and their SNP rsid are shown. (C) Stacked bar plot showing the number of shared DE genes of the bulk RNA-seq data and the scRNA-seq data with the genes of the top eQTLs and sQTLs. The color represents the omics type. (D) Stacked bar plot showing the number of shared DE genes across the bulk RNA-seq data, the scRNA-seq data, genes of the top eQTLs and sQTLs. Colors represent the cell types to which the genes belonged with reference to the DE genes of the cell types in the scRNA-seq data. **Fig. S8.** Comprehensive workflow for scATAC-Seq data analyses in SCA V1.0.0.

**Additional file 2:Table S1.** Cell counts of the adult and fetal tissue groups at each omics level. **Table S2.** Filtered matrix raw read counts for scRNA-Seq across tissues in both fetal and adult groups. Cell_Count_Filtered_Matrix column represents raw read counts initially obtained from published studies or after filtering for the removal of background noises. **Table S3.** Statistics of the upregulated genes from adult and fetal tissues, filtered by average Log2FoldChange > 0.25 and adjusted P of 0.05. Clusters represent cell types. Genes were ranked by average log2-fold-change. **Table S4.** Top receptor–ligand interaction profiles of the cell types in the 38 matching adult and fetal tissues. Interaction analysis was done separately for each tissue, and information on the interaction pairs can be viewed from the first column. Table S5: Top clonotypes (VDJ gene combinations) of each cell type present in the T and B cell repertoires. **Table S6.** Top TFs in the pseudotime transitions of adult and fetal colon cell types. **Table S7**. Top receptor-ligand pairs in spatial transcriptomics of adult colons (colon 1 and colon 2) as well as in scRNA-seq adult and fetal colons. The first column represents the data type to which the interactions belong. Table ranked by decreasing interaction ratios. **Table S8**. Comparison of SCA with other single-cell omics databases. Green tick indicates a yes and a red cross indicates a no. **Table S9.** List of public resources included in the SCA database portal. SCA_PID refers to SCA-designated project identity number (PID).

**Additional file 3.** Supplementary Methods.

**Additional file 4.** Review history.

## Data Availability

This paper used and analyzed publicly available data sets and their resource references are available at http://www.singlecellatlas.org. Codes used for the construction of the database, data analysis, and visualization have been deposited on GitHub and can be accessed via https://github.com/eudoraleer/sca and is under the MIT License [82], and is also on Zenodo at https://zenodo.org/records/10906053 [83]. Web-based platforms hosting the interactive atlas and database queries are available at https://www.singlecellatlas.org.
