# Supplementary material for: Single Cell Atlas: a single-cell multi-omics human cell encyclopedia
Source: Genome Biol. 2024 Apr 19;25:104. doi: 10.1186/s13059-024-03246-2 (PMC11027364; doi:10.1186/s13059-024-03246-2)
Supplement: Supplementary file 1 — Additional file 1:Figure S1. Sample count in fetal and adult groups across tissues and omics types. Figure S2. Correlations between cell types based on gene expression signatures revealed distinct cell type class clusters. (A-B) Heatmap showing the correlations of the cell types from adult (A) and fetal (B) cell types based on the expression of their top upregulated genes. The intensity of the heatmap shows the AUROC level between cell types. Colour blocks on the top of the heatmap represent tissues (first row from the top), biological systems (second row), cell types (third row) and cell type classes (fourth row). Figure S3. Correlations between cell types based on TF signatures revealed similar clustering patterns. (A-B) Heatmap showing the correlations of the cell types from adult (A) and fetal (B) cell types based on the expression of the TF signatures of each cell type. The intensity of the heatmap shows the AUROC level between cell types. Colour blocks on the top of the heatmap represent tissues (first row from the top), biological systems (second row), cell types (third row) and cell type classes (fourth row). Figure S4. Phenotype or disease trait associations. Forest plot showing the associations of phenotype or disease traits in selected cell type classes of scRNA-seq data for both adult and fetal tissues. The X-axis displays the odds ratio of each trait, and the colors of the points represent cell type classes. Figure S5. Landscape of clonal expansion patterns across tissues. (A) tSNE of the tissues from the multi-modal tissues of the scImmune-profiling data. Colors indicate clonal type expansion groups of the cells. Cells not present in the T or B repertoires are colored gray (NA group). Tissues with too few cells present in the T or B repertoires were filtered (i.e., bile duct and kidney) in the main analysis. (B) Stacked bar plots revealing the overall clonal expansion landscapes of the T and B cell repertoires. Colors represent clonal type groups. (C) [file 13059_2024_3246_MOESM1_ESM.pdf]

Fig. S1

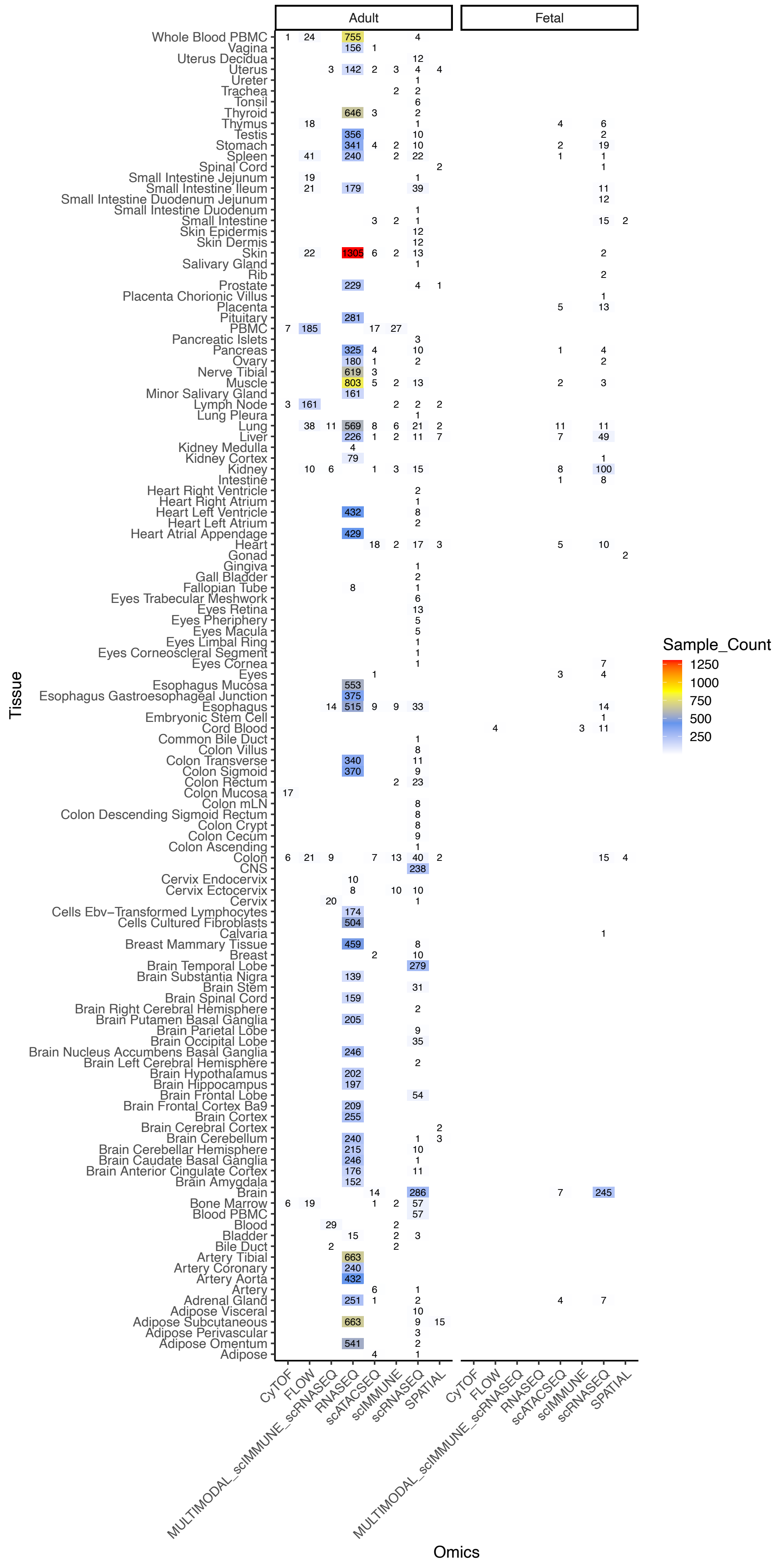

Fig. S1. Sample count in fetal and adult groups across tissues and omics types.

Fig. S2

A

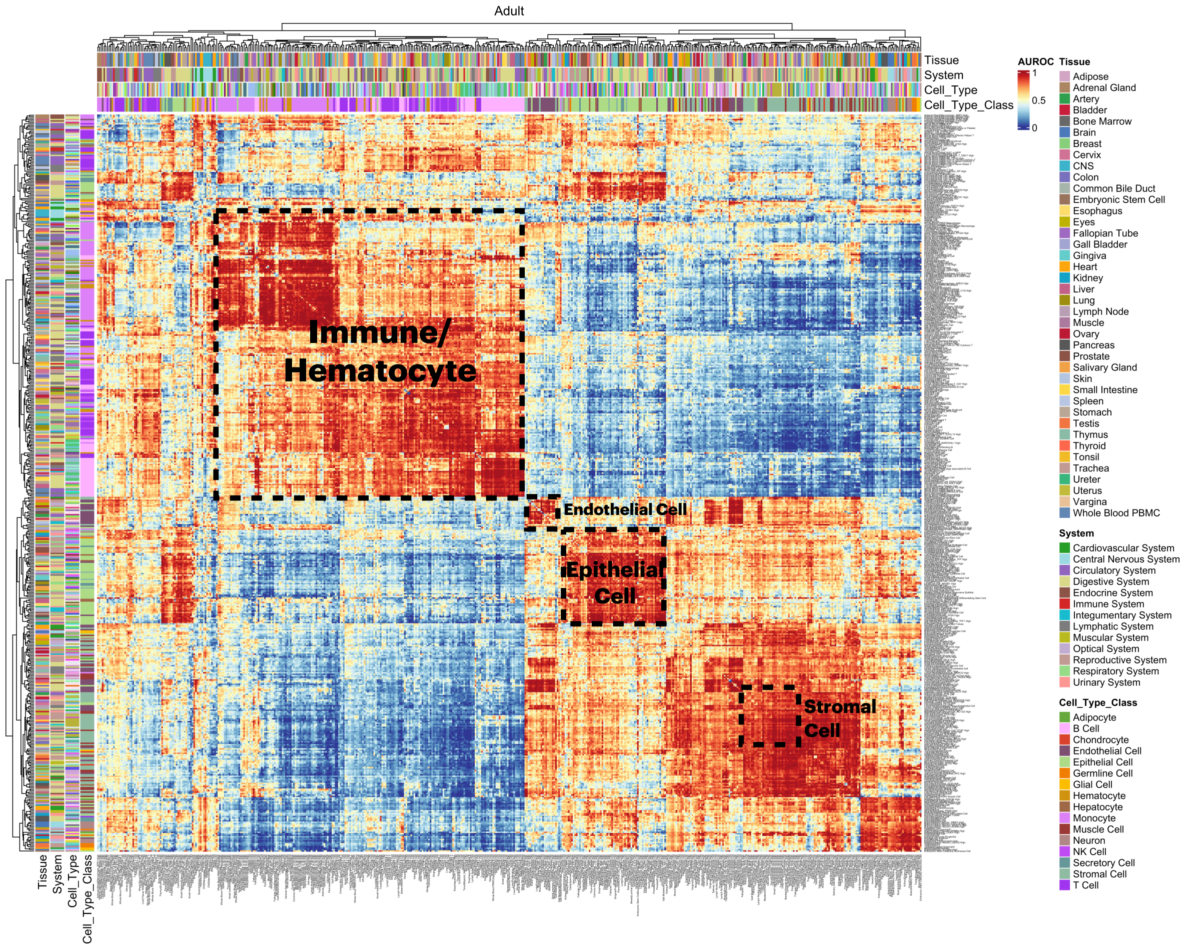

Adult
Tissue
System
Cell_Type
Cell_Type_Class
Immune/ Hematocyte
Endothelial Cell
Epithelial Cell
Stromal Cell
AUROC
1
0.5
0
Tissue
Adipose
Adrenal Gland
Artery
Bladder
Bone Marrow
Brain
Breast
Cervix
CNS
Colon
Common Bile Duct
Embryonic Stem Cell
Esophagus
Eyes
Fallopian Tube
Gall Bladder
Gingiva
Heart
Kidney
Liver
Lung
Lymph Node
Muscle
Ovary
Pancreas
Prostate
Salivary Gland
Skin
Small Intestine
Spleen
Stomach
Testis
Thymus
Thyroid
Tonsil
Trachea
Ureter
Uterus
Vargina
Whole Blood PBMC
System
Cardiovascular System
Central Nervous System
Circulatory System
Digestive System
Endocrine System
Immune System
Integumentary System
Lymphatic System
Muscular System
Optical System
Reproductive System
Respiratory System
Urinary System
Cell_Type_Class
Adipocyte
B Cell
Chondrocyte
Endothelial Cell
Epithelial Cell
Germline Cell
Glial Cell
Hematocyte
Hepatocyte
Monocyte
Muscle Cell
Neuron
NK Cell
Secretory Cell
Stromal Cell
T Cell
Tissue
System
Cell_Type
Cell_Type_Class

B

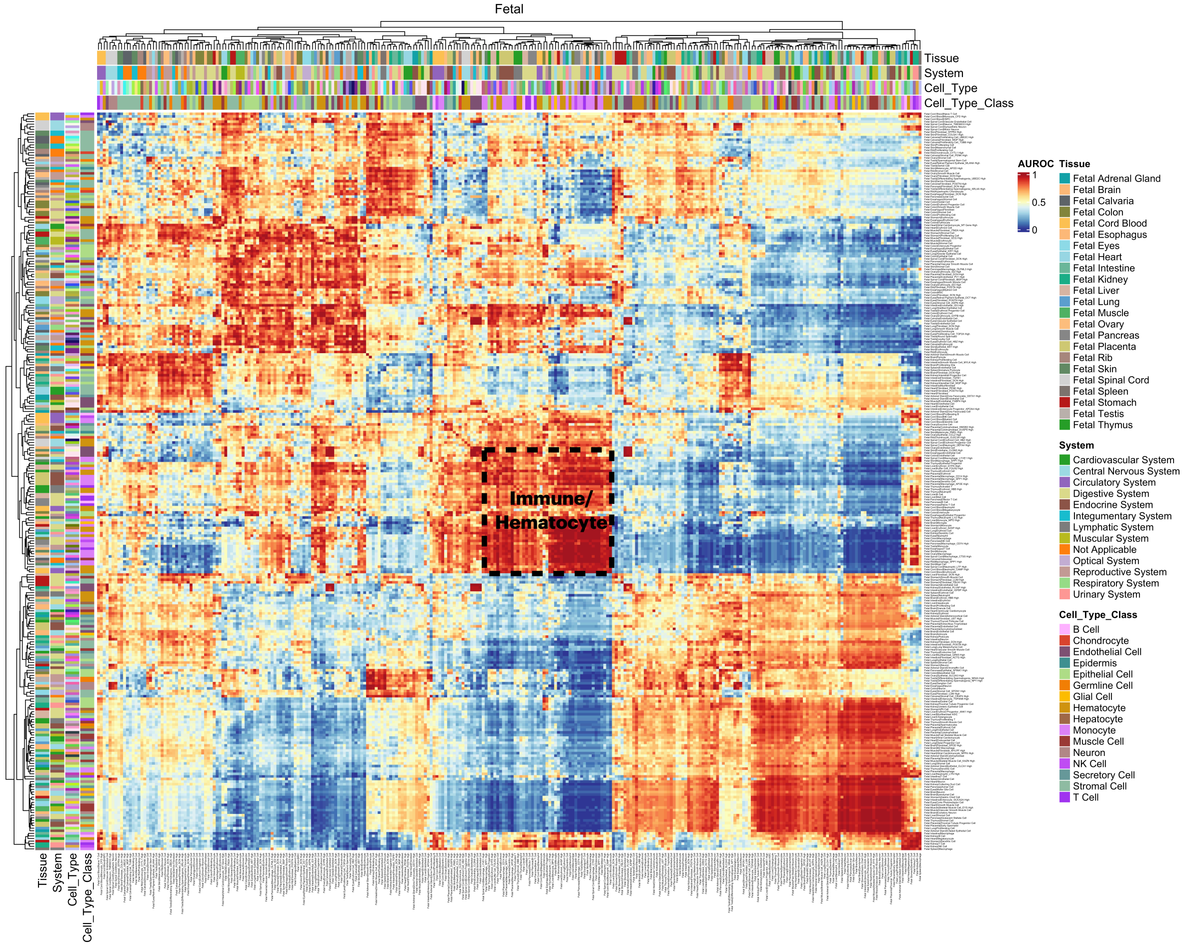

Fetal
Tissue
System
Cell_Type
Cell_Type_Class
Immune/ Hematocyte
AUROC
1
0.5
0
Tissue
Fetal Adrenal Gland
Fetal Brain
Fetal Calvaria
Fetal Colon
Fetal Cord Blood
Fetal Esophagus
Fetal Eyes
Fetal Heart
Fetal Intestine
Fetal Kidney
Fetal Liver
Fetal Lung
Fetal Muscle
Fetal Ovary
Fetal Pancreas
Fetal Placenta
Fetal Rib
Fetal Skin
Fetal Spinal Cord
Fetal Spleen
Fetal Stomach
Fetal Testis
Fetal Thymus
System
Cardiovascular System
Central Nervous System
Circulatory System
Digestive System
Endocrine System
Integumentary System
Lymphatic System
Muscular System
Not Applicable
Optical System
Reproductive System
Respiratory System
Urinary System
Cell_Type_Class
B Cell
Chondrocyte
Endothelial Cell
Epidermis
Epithelial Cell
Germline Cell
Glial Cell
Hematocyte
Hepatocyte
Monocyte
Muscle Cell
Neuron
NK Cell
Secretory Cell
Stromal Cell
T Cell
Tissue
System
Cell_Type
Cell_Type_Class

Fig. S2. Correlations between cell types based on gene expression signatures revealed distinct cell type class clusters.

(A-B) Heatmap showing the correlations of the cell types from adult (A) and fetal (B) cell types based on the expression of their top upregulated genes. The intensity of the heatmap shows the AUROC level between cell types. Colour blocks on the top of the heatmap represent tissues (first row from the top), biological systems (second row), cell types (third row) and cell type classes (fourth row).

Fig. S3

A

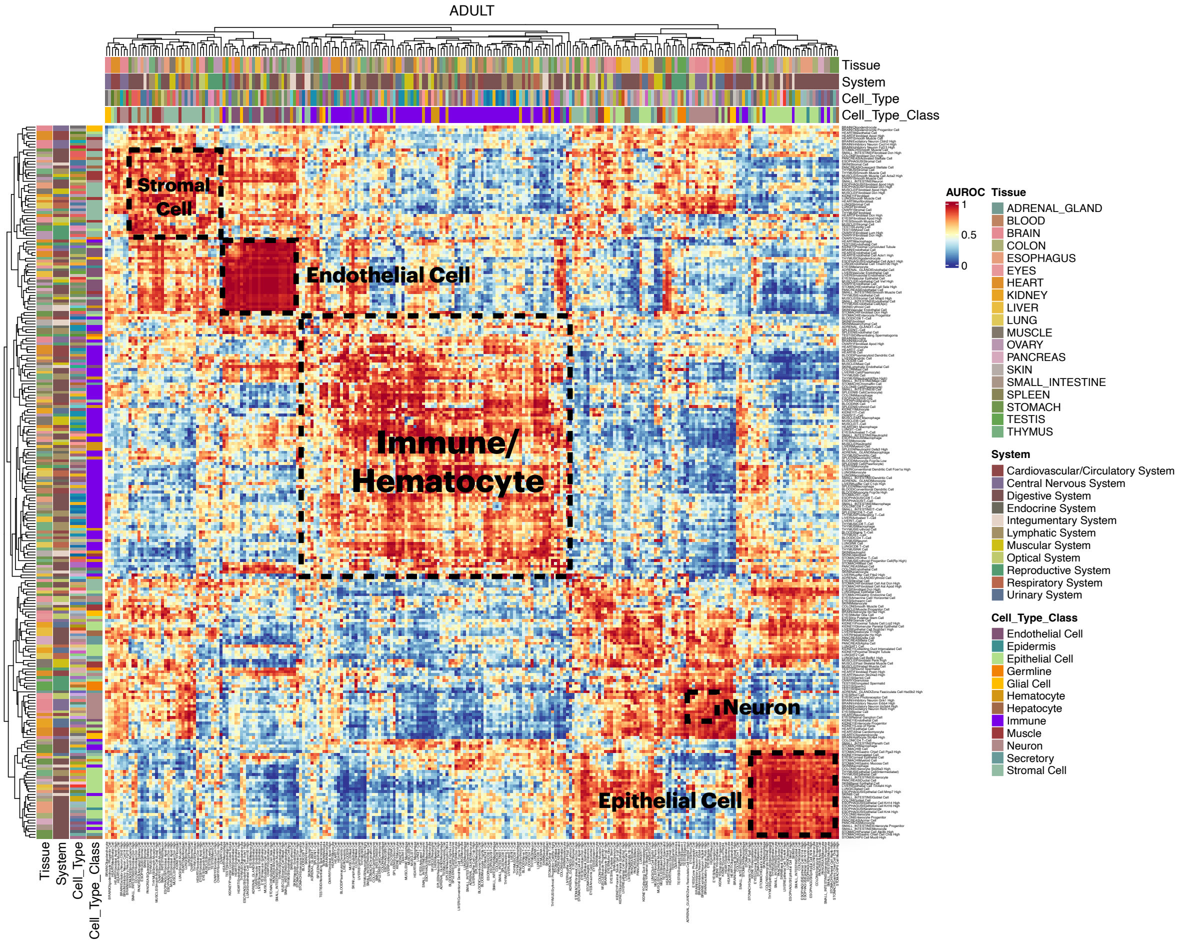
ADULT
Tissue
System
Cell_Type
Cell_Type_Class
Stromal Cell
Endothelial Cell
Immune/ Hematocyte
Neuron
Epithelial Cell
AUROC
1
0.5
0
Tissue
ADRENAL_GLAND
BLOOD
BRAIN
COLON
ESOPHAGUS
EYES
HEART
KIDNEY
LIVER
LUNG
MUSCLE
OVARY
PANCREAS
SKIN
SMALL_INTESTINE
SPLEEN
STOMACH
TESTIS
THYMUS
System
Cardiovascular/Circulatory System
Central Nervous System
Digestive System
Endocrine System
Integumentary System
Lymphatic System
Muscular System
Optical System
Reproductive System
Respiratory System
Urinary System
Cell_Type_Class
Endothelial Cell
Epidermis
Epithelial Cell
Germline
Glial Cell
Hematocyte
Hepatocyte
Immune
Muscle
Neuron
Secretory
Stromal Cell

B

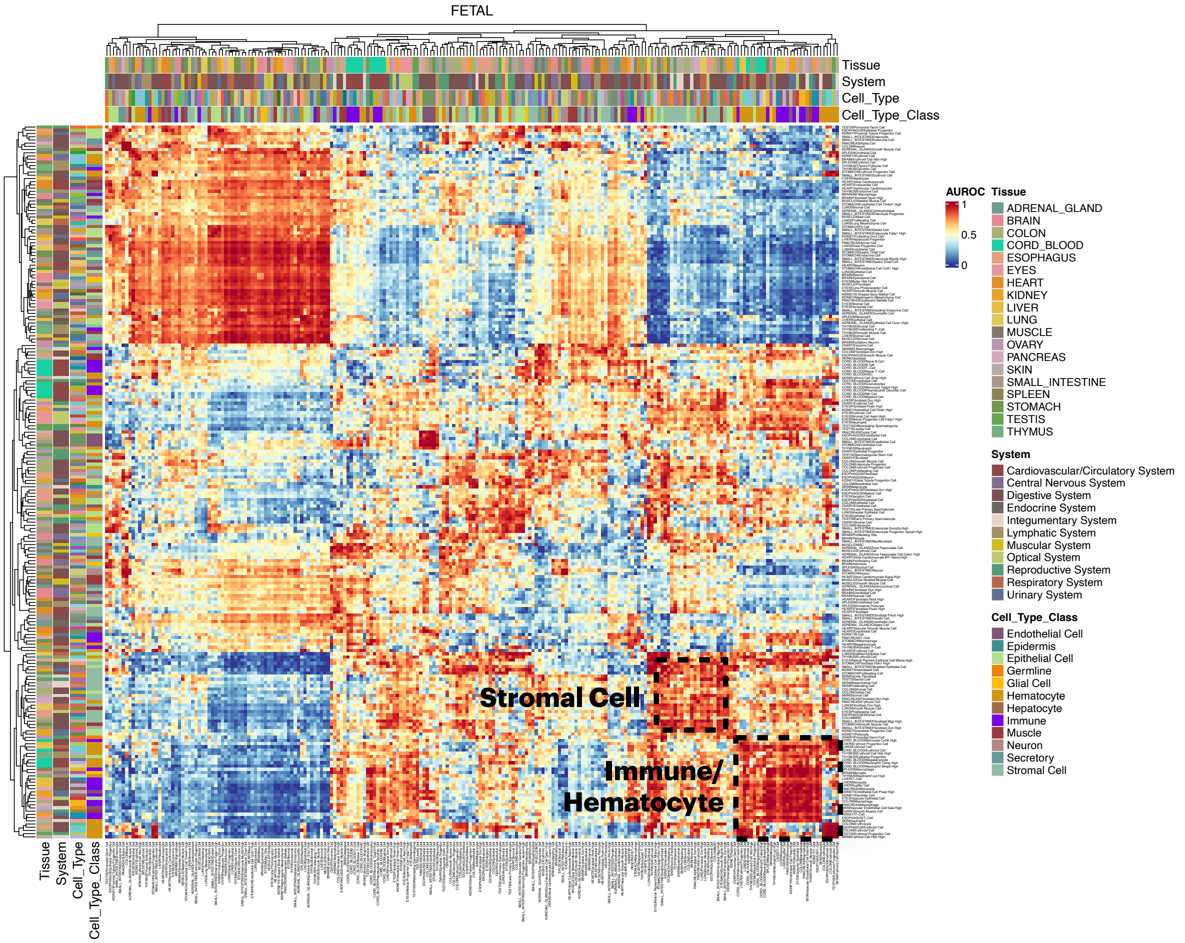
FETAL
Tissue
System
Cell_Type
Cell_Type_Class
Stromal Cell
Immune/ Hematocyte
AUROC
1
0.5
0
Tissue
ADRENAL_GLAND
BRAIN
COLON
CORD_BLOOD
ESOPHAGUS
EYES
HEART
KIDNEY
LIVER
LUNG
MUSCLE
OVARY
PANCREAS
SKIN
SMALL_INTESTINE
SPLEEN
STOMACH
TESTIS
THYMUS
System
Cardiovascular/Circulatory System
Central Nervous System
Digestive System
Endocrine System
Integumentary System
Lymphatic System
Muscular System
Optical System
Reproductive System
Respiratory System
Urinary System
Cell_Type_Class
Endothelial Cell
Epidermis
Epithelial Cell
Germline
Glial Cell
Hematocyte
Hepatocyte
Immune
Muscle
Neuron
Secretory
Stromal Cell

Fig. S3. Correlations between cell types based on TF signatures revealed similar clustering patterns.

(A-B) Heatmap showing the correlations of the cell types from adult (A) and fetal (B) cell types based on the expression of the TF signatures of each cell type. The intensity of the heatmap shows the AUROC level between cell types. Colour blocks on the top of the heatmap represent tissues (first row from the top), biological systems (second row), cell types (third row) and cell type classes (fourth row).

Fig. S4

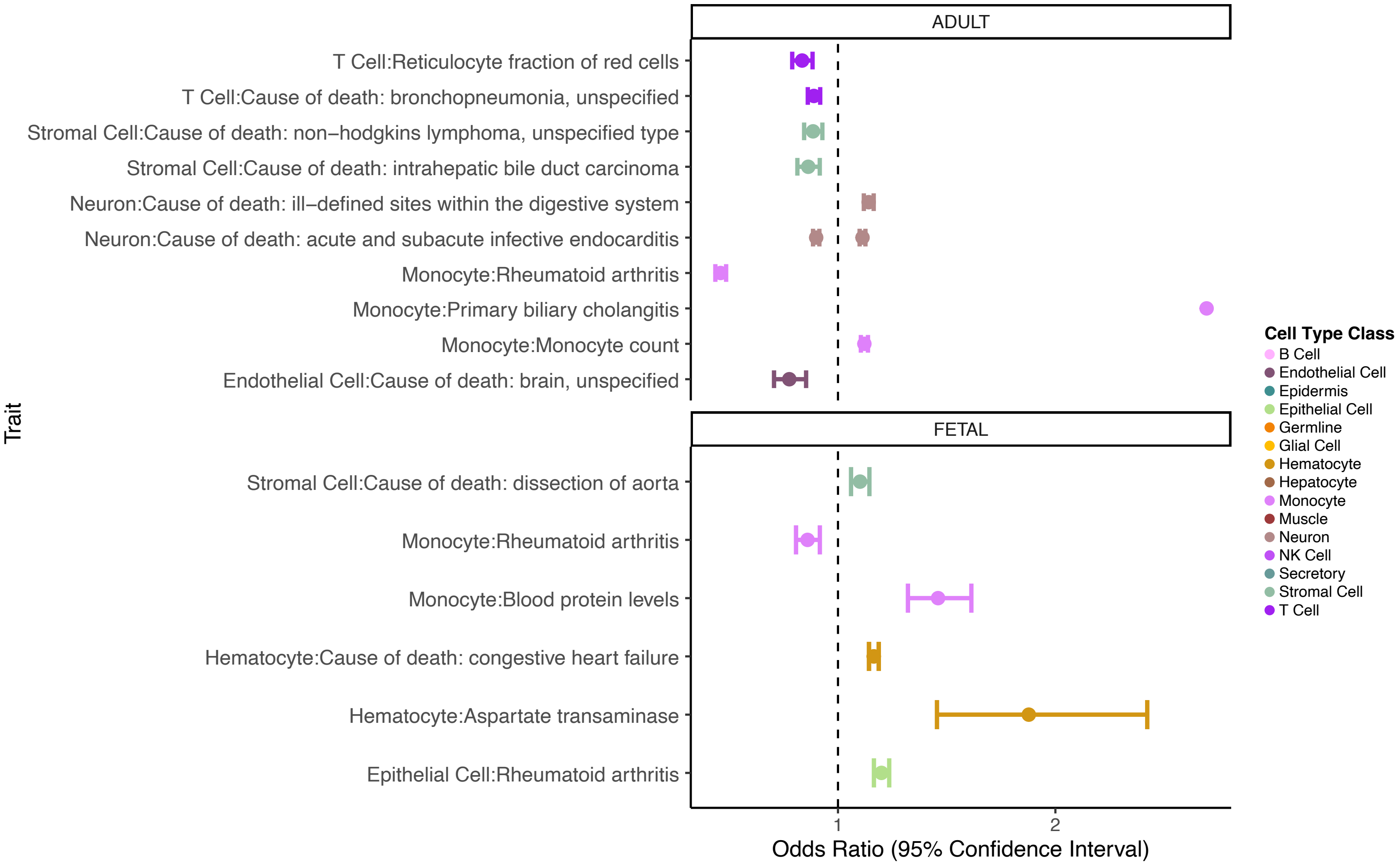

Fig. S4. Phenotype or disease trait associations. Forest plot showing the associations of phenotype or disease traits in selected cell type classes of scRNA-seq data for both adult and fetal tissues. The X-axis displays the odds ratio of each trait, and the colors of the points represent cell type classes.

Fig. S5

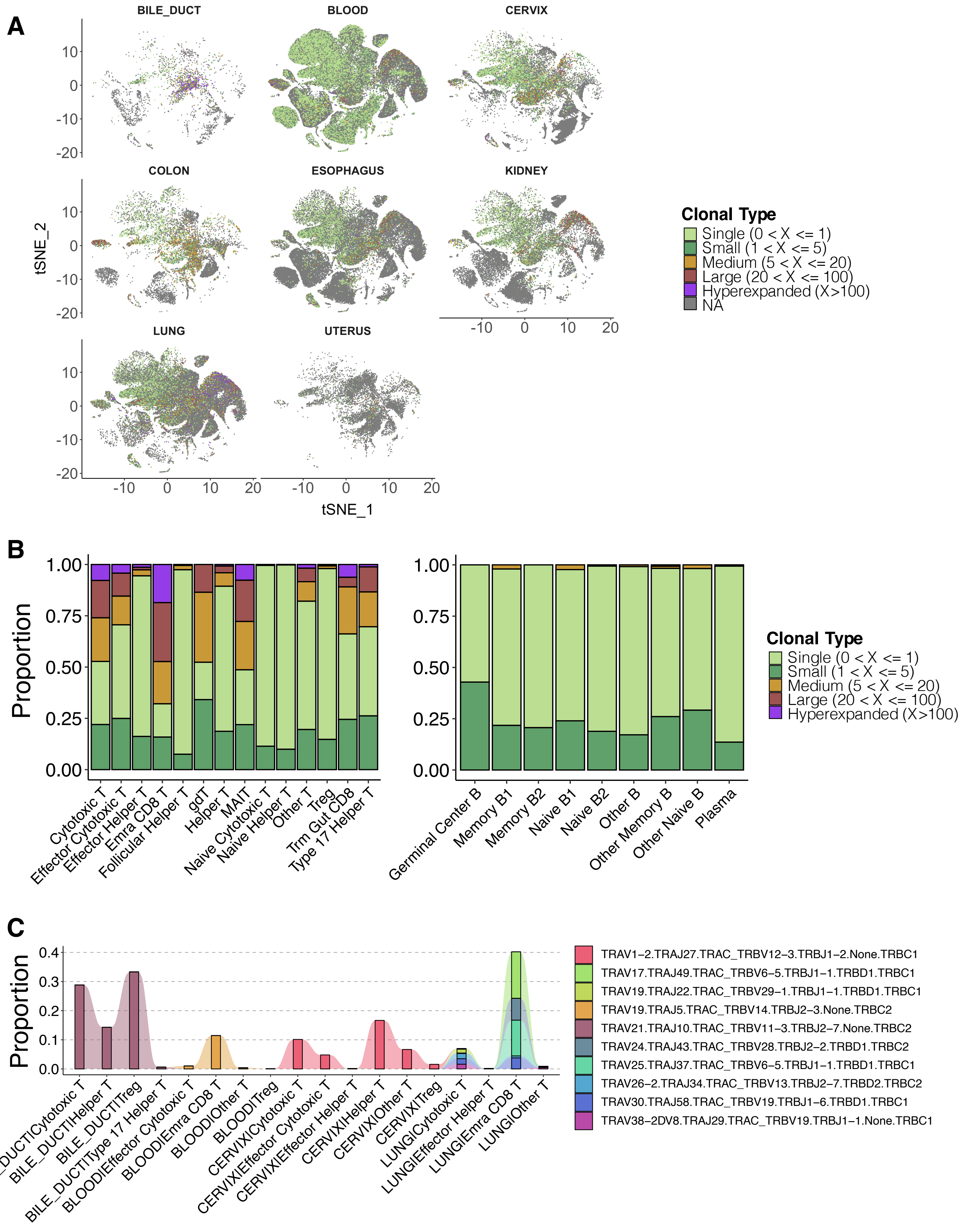
A
BILE_DUCT
BLOOD
CERVIX
COLON
ESOPHAGUS
KIDNEY
LUNG
UTERUS
tSNE_2
tSNE_1
Clonal Type
Single (0 < X <= 1)
Small (1 < X <= 5)
Medium (5 < X <= 20)
Large (20 < X <= 100)
Hyperexpanded (X>100)
NA
B
Proportion
Cytotoxic T
Effector Cytotoxic T
Effector Helper T
Emra CD8 T
Follicular Helper T
gdT
Helper T
MAIT
Naive Cytotoxic T
Naive Helper T
Other T
Treg
Trm Gut CD8
Type 17 Helper T
Germinal Center B
Memory B1
Memory B2
Naive B1
Naive B2
Other B
Other Memory B
Other Naive B
Plasma
Clonal Type
Single (0 < X <= 1)
Small (1 < X <= 5)
Medium (5 < X <= 20)
Large (20 < X <= 100)
Hyperexpanded (X>100)
C
Proportion
BILE_DUCT|Cytotoxic T
BILE_DUCT|Helper T
BILE_DUCT|Treg
BILE_DUCT|Type 17 Helper T
BLOOD|Effector Cytotoxic T
BLOOD|Emra CD8 T
BLOOD|Other T
BLOOD|Treg
CERVIX|Cytotoxic T
CERVIX|Effector Cytotoxic T
CERVIX|Effector Helper T
CERVIX|Helper T
CERVIX|Other T
CERVIX|Treg
LUNG|Cytotoxic T
LUNG|Effector Helper T
LUNG|Emra CD8 T
LUNG|Other T
TRAV1–2.TRAJ27.TRAC_TRBV12–3.TRBJ1–2.None.TRBC1
TRAV17.TRAJ49.TRAC_TRBV6–5.TRBJ1–1.TRBD1.TRBC1
TRAV19.TRAJ22.TRAC_TRBV29–1.TRBJ1–1.TRBD1.TRBC1
TRAV19.TRAJ5.TRAC_TRBV14.TRBJ2–3.None.TRBC2
TRAV21.TRAJ10.TRAC_TRBV11–3.TRBJ2–7.None.TRBC2
TRAV24.TRAJ43.TRAC_TRBV28.TRBJ2–2.TRBD1.TRBC2
TRAV25.TRAJ37.TRAC_TRBV6–5.TRBJ1–1.TRBD1.TRBC1
TRAV26–2.TRAJ34.TRAC_TRBV13.TRBJ2–7.TRBD2.TRBC2
TRAV30.TRAJ58.TRAC_TRBV19.TRBJ1–6.TRBD1.TRBC1
TRAV38–2DV8.TRAJ29.TRAC_TRBV19.TRBJ1–1.None.TRBC1

Fig. S5. Landscape of clonal expansion patterns across tissues.

(A) tSNE of the tissues from the multi-modal tissues of the scImmune-profiling data. Colors indicate clonal type expansion groups of the cells. Cells not present in the T or B repertoires are colored gray (NA group). Tissues with too few cells present in the T or B repertoires were filtered (i.e., bile duct and kidney) in the main analysis.

(B) Stacked bar plots revealing the overall clonal expansion landscapes of the T and B-cell repertoires. Colors represent clonal type groups.

(C) Alluvial plot showing the top clonal types in T-cell repertoires and their proportions shared across tissues containing these clonotypes. Colors represent clonotypes.

Fig. S6

A

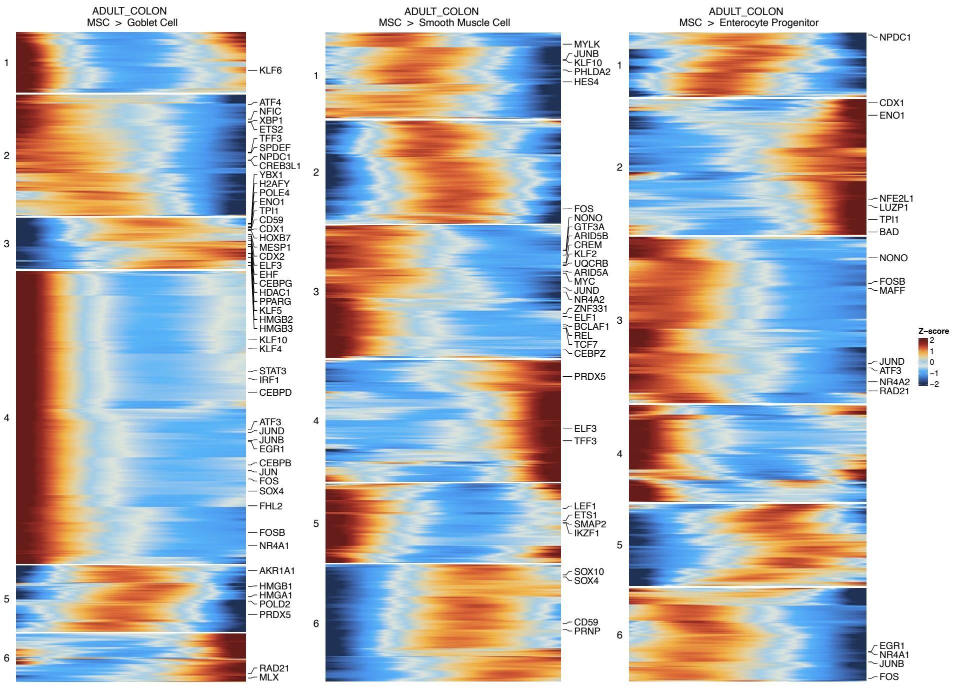
ADULT_COLON
MSC > Goblet Cell
KLF6
ATF4
NFIC
XBP1
ETS2
TFF3
SPDEF
NPDC1
CREB3L1
YBX1
H2AFY
POLE4
ENO1
TPI1
CD59
CDX1
HOXB7
MESP1
CDX2
ELF3
EHF
CEBPG
HDAC1
PPARG
KLF5
HMGB2
HMGB3
KLF10
KLF4
STAT3
IRF1
CEBPD
ATF3
JUND
JUNB
EGR1
CEBPB
JUN
FOS
SOX4
FHL2
FOSB
NR4A1
AKR1A1
HMGB1
HMGA1
POLD2
PRDX5
RAD21
MLX
ADULT_COLON
MSC > Smooth Muscle Cell
MYLK
JUNB
KLF10
PHLDA2
HES4
FOS
NONO
GTF3A
ARID5B
CREM
KLF2
UQCRB
ARID5A
MYC
JUND
NR4A2
ZNF331
ELF1
BCLAF1
REL
TCF7
CEBPZ
PRDX5
ELF3
TFF3
LEF1
ETS1
SMAP2
IKZF1
SOX10
SOX4
CD59
PRNP
ADULT_COLON
MSC > Enterocyte Progenitor
NPDC1
CDX1
ENO1
NFE2L1
LUZP1
TPI1
BAD
NONO
FOSB
MAFF
JUND
ATF3
NR4A2
RAD21
EGR1
NR4A1
JUNB
FOS
Z-score
2
1
0
−1
−2

B

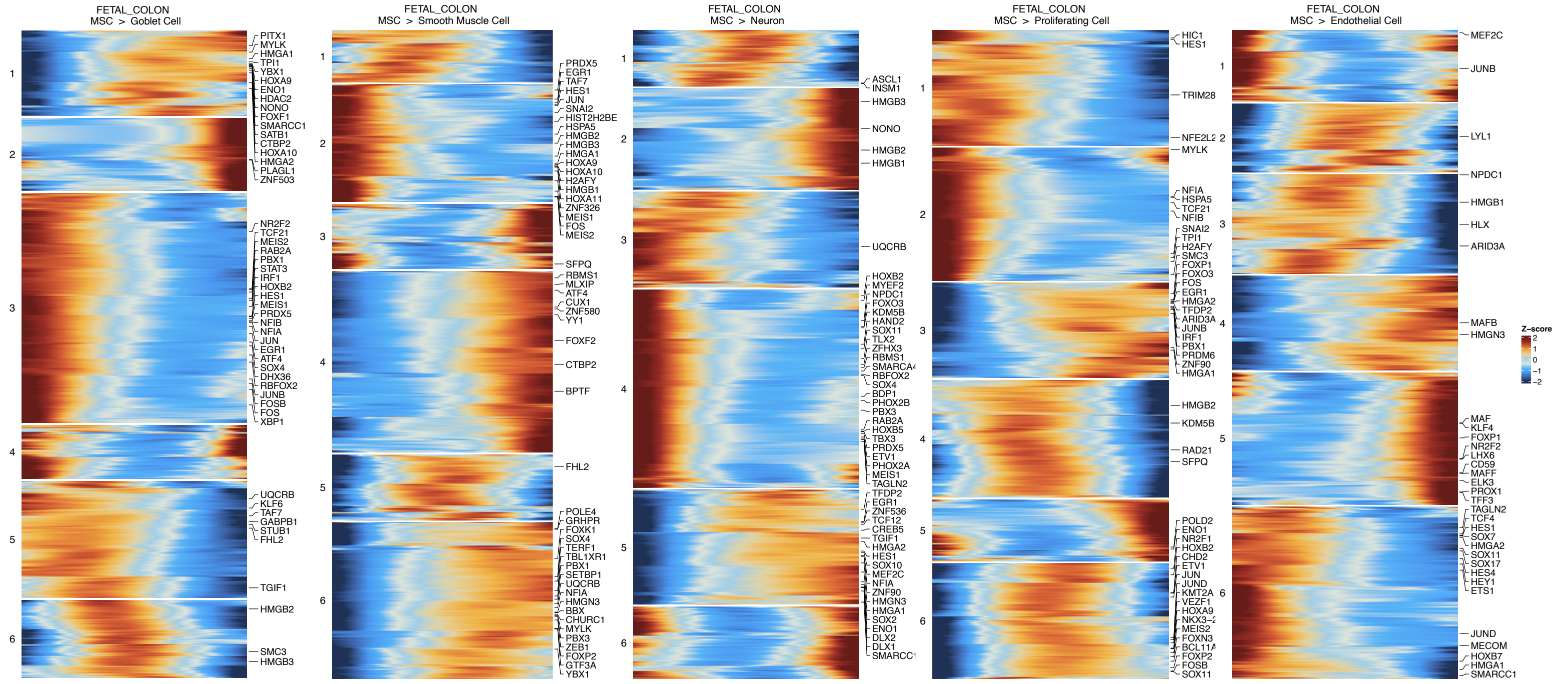
FETAL_COLON
MSC > Goblet Cell
PITX1
MYLK
HMGA1
TPI1
YBX1
HOXA9
ENO1
HDAC2
NONO
FOXF1
SMARCC1
SATB1
CTBP2
HOXA10
HMGA2
PLAGL1
ZNF503
NR2F2
TCF21
MEIS2
RAB2A
PBX1
STAT3
IRF1
HOXB2
HES1
MEIS1
PRDX5
NFIB
NFIA
JUN
EGR1
ATF4
SOX4
DHX36
RBFOX2
JUNB
FOSB
FOS
XBP1
UQCRB
KLF6
TAF7
GABPB1
STUB1
FHL2
TGIF1
HMGB2
SMC3
HMGB3
FETAL_COLON
MSC > Smooth Muscle Cell
PRDX5
EGR1
TAF7
HES1
JUN
SNAI2
HIST2H2BE
HSPA5
HMGB2
HMGB3
HMGA1
HOXA9
HOXA10
H2AFY
HMGB1
HOXA11
ZNF326
MEIS1
FOS
MEIS2
SFPQ
RBMS1
MLXIP
ATF4
CUX1
ZNF580
YY1
FOXF2
CTBP2
BPTF
FHL2
POLE4
GRHPR
FOXK1
SOX4
TERF1
TBL1XR1
PBX1
SETBP1
UQCRB
NFIA
HMGN3
BBX
CHURC1
MYLK
PBX3
ZEB1
FOXP2
GTF3A
YBX1
FETAL_COLON
MSC > Neuron
ASCL1
INSM1
HMGB3
NONO
HMGB2
HMGB1
UQCRB
HOXB2
MYEF2
NPDC1
FOXO3
KDM5B
HAND2
SOX11
TLX2
ZFHX3
RBMS1
RBFOX2
SOX4
BDP1
PHOX2B
PBX3
RAB2A
HOXB5
TBX3
PRDX5
ETV1
PHOX2A
MEIS1
TAGLN2
TFDP2
EGR1
ZNF536
TCF12
CREB5
TGIF1
HMGA2
HES1
SOX10
MEF2C
NFIA
ZNF90
HMGN3
HMGA1
SOX2
ENO1
DLX2
DLX1
FETAL_COLON
MSC > Proliferating Cell
HIC1
HES1
TRIM28
NFE2L2
MYLK
NFIA
HSPA5
TCF21
NFIB
SNAI2
TPI1
H2AFY
SMC3
FOXP1
FOXO3
FOS
EGR1
HMGA2
TFDP2
ARID3A
JUNB
IRF1
PBX1
PRDM6
ZNF90
HMGA1
HMGB2
KDM5B
RAD21
SFPQ
POLD2
ENO1
NR2F1
HOXB2
CHD2
ETV1
JUN
JUND
KMT2A
VEZF1
HOXA9
MEIS2
FOXN3
FOXP2
FOSB
SOX11
FETAL_COLON
MSC > Endothelial Cell
MEF2C
JUNB
LYL1
NPDC1
HMGB1
HLX
ARID3A
MAFB
HMGN3
MAF
KLF4
FOXP1
NR2F2
LHX6
CD59
MAFF
ELK3
PROX1
TFF3
TAGLN2
TCF4
HES1
SOX7
HMGA2
SOX11
SOX17
HES4
HEY1
ETS1
JUND
MECOM
HOXB7
HMGA1
SMARCC1
Z-score
2
1
0
−1
−2

Fig. S6. Pseudotime heatmaps of MSC lineage cell types in the adult and fetal colon.

(A-B) Pseudotime trajectory of each cell type in the MSC lineage of adult (A) and fetal (B) colons. The color represents the cell type, and the violin plots represent the density of cells across pseudotime.

Fig. S7

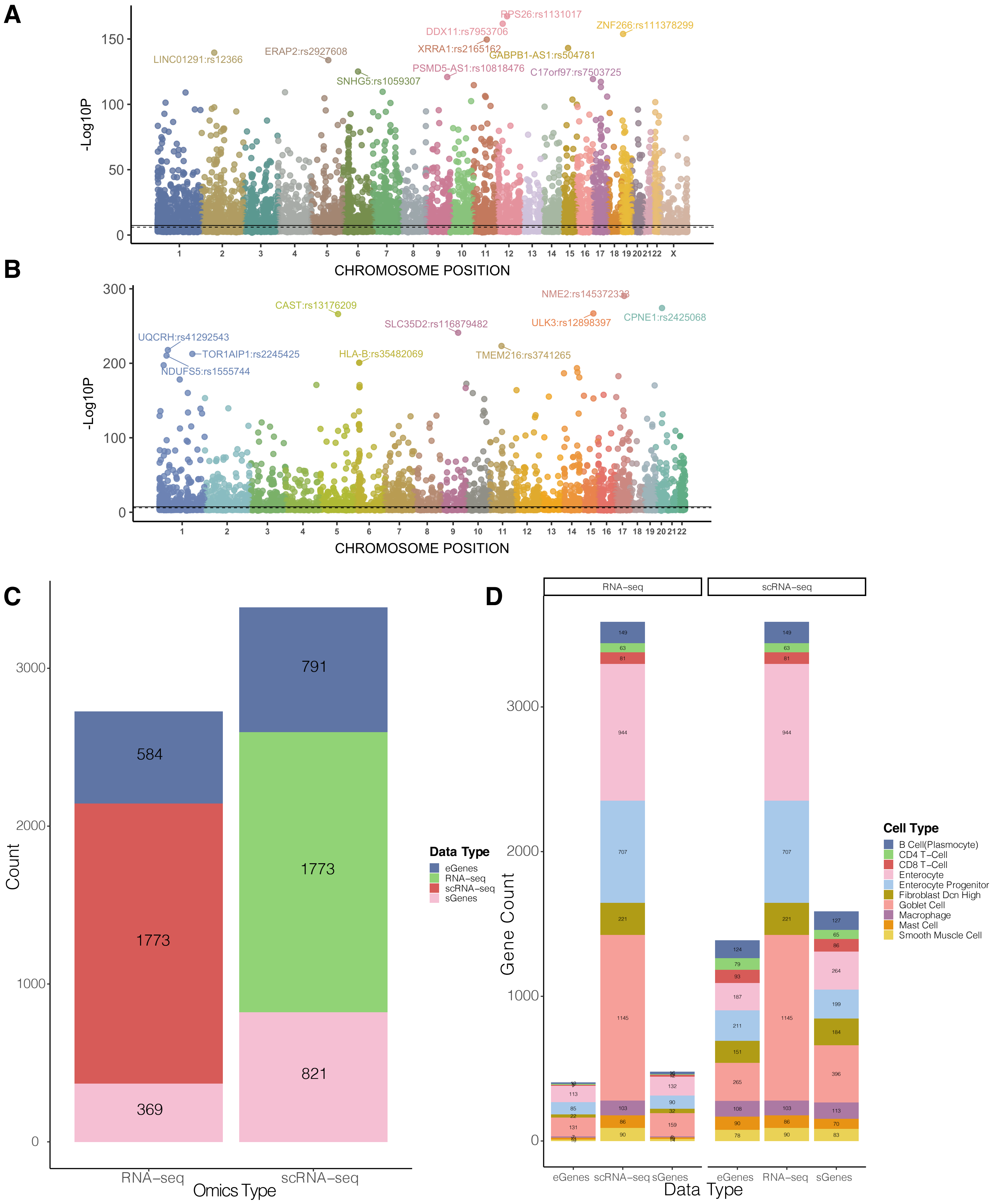
A
-Log10P
150
100
50
0
LINC01291:rs12366
ERAP2:rs2927608
SNHG5:rs1059307
DDX11:rs7953706
XRRA1:rs2165162
PSMD5-AS1:rs10818476
RPS26:rs1131017
GABPB1-AS1:rs504781
C17orf97:rs7503725
ZNF266:rs111378299
1 2 3 4 5 6 7 8 9 10 11 12 13 14 15 16 17 18 19 20 21 22 X
CHROMOSOME POSITION
B
-Log10P
300
200
100
0
UQCRH:rs41292543
TOR1AIP1:rs2245425
NDUFS5:rs1555744
CAST:rs13176209
HLA-B:rs35482069
SLC35D2:rs116879482
TMEM216:rs3741265
NME2:rs145372332
ULK3:rs12898397
CPNE1:rs2425068
1 2 3 4 5 6 7 8 9 10 11 12 13 14 15 16 17 18 19 20 21 22
CHROMOSOME POSITION
C
Count
3000
2000
1000
0
584
1773
369
791
1773
821
RNA–seq
scRNA–seq
Omics Type
Data Type
eGenes
RNA–seq
scRNA–seq
sGenes
D
RNA–seq
scRNA–seq
Gene Count
3000
2000
1000
0
149
63
81
944
707
221
1145
103
86
90
113
85
22
131
132
90
32
159
124
79
93
187
211
151
265
108
90
78
127
65
86
264
199
184
396
113
70
83
eGenes
scRNA–seq
sGenes
eGenes
RNA–seq
sGenes
Data Type
Cell Type
B Cell(Plasmocyte)
CD4 T–Cell
CD8 T–Cell
Enterocyte
Enterocyte Progenitor
Fibroblast Dcn High
Goblet Cell
Macrophage
Mast Cell
Smooth Muscle Cell

Fig. S7. Comparison of DE gene overlaps between bulk RNA-seq, scRNA-seq and WGS.

(A) Chromosomal positions of the top 10 eGenes in colon transverse bulk RNA-seq data. Gene names and their SNP rsid are shown.

(B) Chromosomal positions of the top 10 sGenes in colon transverse bulk RNA-seq data. Gene names and their SNP rsid are shown.

(C) Stacked bar plot showing the number of shared DE genes of the bulk RNA-seq data and the scRNA-seq data with the genes of the top eQTLs and sQTLs. The color represents the omics type.

(D) Stacked bar plot showing the number of shared DE genes across the bulk RNA-seq data, the scRNA-seq data, genes of the top eQTLs and sQTLs. Colors represent the cell types to which the genes belonged with reference to the DE genes of the cell types in the scRNA-seq data.

Fig. S8

scATAC-Seq Workflow

SCA V1.0.0

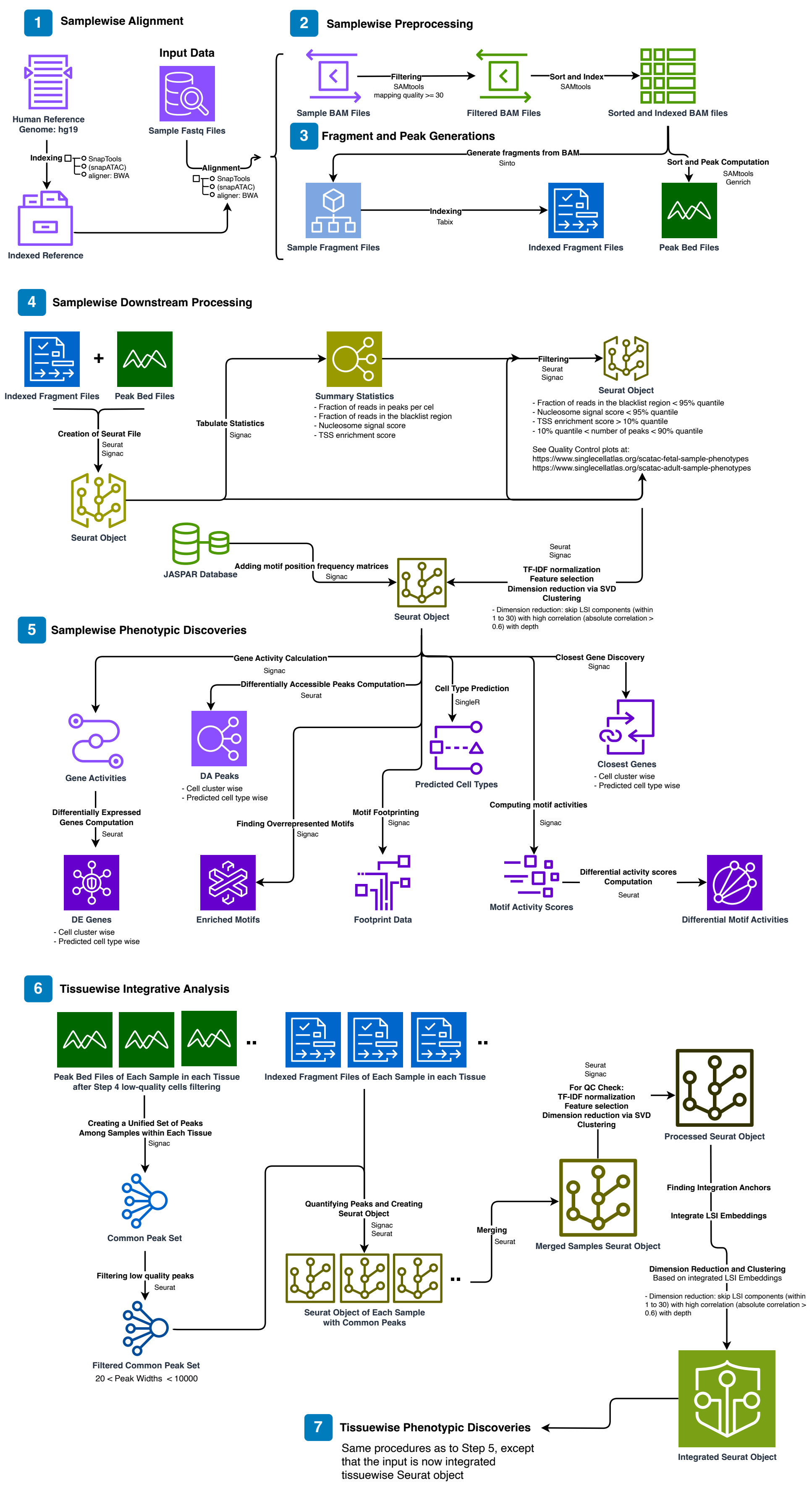

Fig. S8. Comprehensive workflow for scATAC-Seq data analyses in SCA V1.0.0.
