# Supplementary material for: Single Cell Atlas: a single-cell multi-omics human cell encyclopedia
Source: Genome Biol. 2024 Apr 19;25:104. doi: 10.1186/s13059-024-03246-2 (PMC11027364; doi:10.1186/s13059-024-03246-2)
Supplement: Supplementary file 3 — Additional file 3. Supplementary Methods. [file 13059_2024_3246_MOESM3_ESM.docx]

**Supplementary Methods**

Data source and data retrieval

To construct the database, we first retrieved raw read counts (produced after quantification) of five single-cell omics (i.e., scRNA-seq, scATAC-Seq, scImmune, CyTOF, and flow cytometry), spatial transcriptomics, and two bulk omics (i.e., RNA-seq, and WGS) from publicly available data sources (refer to Additional file 2: Table S9 or <https://www.singlecellatlas.org/sources>), covering 125 healthy adult and fetal tissues. Specifically, for some of the 10X Genomics scRNA-Seq outputs with filtered_feature_matrix files readily available online, we utilized their filtered_feature_matrix files instead of the raw_feature_matrix files as raw matrices contain large number of empty droplets. For others without publicly available filtered_feature_matrix files, we measured noises in their raw feature matrices based on the inflection point of the number of barcodes to UMI count rank plot curve for each of these samples to obtain filtered matrices similar with background noise removed. Analyses were performed on filtered read count matrices of each sample, and integrative analyses were performed at the tissue level across tissues in single or multiomics ways.

Data processing and quality control

**ScRNA-sequencing:** For each sample, cells with less than or equal to 200 detected expressed genes/cells were removed to filter off low-quality cells or potential empty droplets for droplet-based libraries. Cells with more than 10% of the mitochondrial genes expressed were identified as damaged cells and were discarded. Using deconvolution approach [1] and unique cell-state gene expression identification, doublets were removed while retaining transitioning cells with similar phenotypic characteristics. Each cell was then normalized using Seurat [2] by taking the read count of each gene of the cell divided by the total read count of the cell, multiplied by a scale factor of 10,000. The values were then natural-log-transformed and scaled via z-score transformation using highly variable (HV) genes across cells, identified using the variance-stabilizing transformation (VST) [2].

**ScATAC-Sequencing:** Samples mapped with genome reference other than hg19 have conducted alignment and quantification from raw fastq files using genome reference hg19 using SnapTools from SnapATAC [3]. SAMtools [4] was used for filtering the bam files based on options ‘-f 2 -F 0x70c -q 30’, followed by sorting and indexing. A detailed workflow is enclosed in Additional file 1: Fig. S8. For each sample, cells with the following criteria were retained (see Step 4, Samplewise Downstream Processing in Additional file 1: Fig. S8); cells (i) with the fraction of reads in the blacklist region being less than 95% quantile of the fraction of reads in the blacklist region across all cells; (ii) nucleosome signal score less than 95% quantile of the nucleosome signal scores across all cells; (iii) TSS enrichment score larger than 10% quantile of the TSS enrichment scores across all cells; (iv) and the number of peaks that are more than10% quantile and smaller than 90% quantile of the number of peaks across all cells. Cells not fulfilling these criteria might be a sign of doublets or artifacts and were removed using Signac [5]. As part of the latent semantic indexing (LSI) process, normalization was done using the frequency-inverse document frequency (TF-IDF) procedure on each sample to correct for sequencing depths between cells and across peaks. Feature selection was then carried out using the VST method to identify top peaks for use in the subsequent steps of the analysis (Additional file 1: Fig. S8).

**ScImmune profiling:** high-confidence cellular contigs were considered. Clonotypes with missing annotation in all the V, D, and J nucleotide sequences due to either low mapping quality or low confidence assignment of reads due to somatic hypermutations in the gene and junctions were discarded. The T and B repertoires in each sample were processed separately, and reads were converted into different clonotype calls using scRepertoire [6] and immunarch [7]. Clonotype calls included the CDR3 nucleotide sequence, amino acid sequence, VDJC gene sequence, and the combination of CDR3 nucleotide and VDJC gene sequences, which were used for further analyses. Immune data processing of scImmune profiling was done after scRNA-Seq data were fully annotated for multi-modal 10X 5’ V(D)J immune profiling samples [8]. For each sample, T and/or B immune repertoires were mapped onto their scRNA-Seq data via the unique cell identifiers, and cells annotated with non-T or non-B cells that were present in non-T or B-cell clusters but were mapped with T/B repertoire information were considered sequencing errors or other problems, such as doublets or artifacts, and were discarded.

**Spatial transcriptomics:** Since it is a gene expression by spot technology, data normalization was performed using scTransform in Seurat by modelling the gene expression using regularized binomial distribution to account for sequence depth variations across each spot while preserving biological variations. Feature selection was performed to identify highly variable genes across the spots using VST.

**CyTOF:** for each sample, hyperbolic arcsine transformation was applied to the expression matrix with a co-factor value of 5 using flowCore [9]. Cells with the number of RNA molecules outside the 1.5 interquartile range were regarded as low-quality cells or doublets and were removed.

**Flow cytometry:** for each sample, the hyperbolic arcsine transformation was applied with a co-factor value of 150. Gating was performed to remove doublets and to retain live cells using the 1) forward scatter area (FSC-A) versus side scatter area (SSC-A) gating, followed by 2) FSC-A versus forward scatter height (FSC-H) gating, 3) side scatter height (SSC-H) versus side scatter width (SSC-W) gating, and 4) forward scatter width (FSC-W) versus forward scatter height (FSC-H) gating approaches, via openCyto [10].

**RNA-sequencing:** served autolysis samples were removed. edgeR [11] was used for data processing. Counts per million (CPM) values were computed based on the raw gene read counts, and a prior count of 0.25 was added to each observation to avoid any division by zero. Genes expressing at >1 CPM in at least 5% of the samples will be retained. A trimmed mean of M-values (TMM) between each pair of samples was computed to serve as a set of scaling factors for normalizing the library sizes of the samples.

Data integration with batch correction

**ScRNA-sequencing**: To prevent batch effects introduced by different data sources from confounding the result, we treated each data source as a separate batch. Cells were first projected onto a lower dimension under the PCA dimension space with their batch assignments using Seurat while ensuring that the Euclidean distance between cells was preserved in a lower-dimensional space. PCA embeddings were corrected for batch effects using Harmony [12], and a corrected set of lower-dimensional Harmony embeddings was returned. The integration process was done separately for each tissue.

**ScATAC-Sequencing:** For each tissue, a common peak set was created [5] for all samples in the tissue based on their peaks information in the bed files and subsequently followed by the production of a fragment object file for each sample (Additional file 1: Fig. S8). Low quality common peaks were removed based on the criteria stated in Additional file 1: Fig. S8. Based on the common peak sets for samples in each tissue, merging was performed to join samples containing only the common peaks in each tissue. After which, uncorrected LSI embeddings were calculated post-TF-IDF normalization for the merged samples [5]. For each tissue, integration was carried out by identifying shared correlation structures across peaks of different samples using canonical correlation analysis with L2 normalization of the correlation vectors under the LSI projection. LSI coordinates were corrected across the samples based on the uncorrected LSI computed prior. For tissues with only one sample present, no merging and integration were carried out.

**ScImmune profiling:** for T and B immune repertoires, sample-level and combined tissue-level analyses were both performed for each repertoire. For multi-modal integrative analysis of immune repertoires with their corresponding scRNA-Seq data, integration of the scRNA-Seq data was done using Harmony, by assigning each project as an individual batch, and this was done separately for each tissue. Both T and B immune repertoires were then mapped to their corresponding scRNA-Seq data based on their unique cell barcodes in each multi-modal sample.

**Spatial transcriptomics:** each spatial transcriptomics dataset was analyzed separately, and no integration was done for samples with the same or different tissue types.

**CyTOF:** The panel design varied across samples from different projects and was limited by the number of detectable antibodies that could be tagged using naturally available heavy metal isotopes per CyTOF run. Therefore, the number of antibodies in a panel design has less extensive or no overlaps with antibodies used in another panel. Due to this technical limitation, a separate integration procedure was done for samples from the same study and the same panel design. In detail, for each tissue type and each set of samples grouped according to this criterion, samples were processed using arcsine-transformed expression data from the post-data processing step, scaling was carried out, and PCA projections were calculated. Integration anchors between samples were determined using Seurat, and samples were integrated according to the anchors identified to correct for batch effects.

**Flow cytometry:** similar to CyTOF, the panel design varied across projects, and the limitation due to the number of antibodies that can be stained in a single run is even more intensive, i.e., a much lower number of antibodies per panel design can be used. Samples in each group were merged and scaled based on the arcsine-transformed expression data from the postdata processing step. Considering the heightening of time and computational complexities that might arise due to a much larger number of cells present in each group compared to CyTOF after merging, similar to Harmony integration in scRNA-Seq, Harmony was used for integration with a significant reduction in integration time while maintaining similar integration efficacy. The integration process was performed separately for each tissue.

**RNA-sequencing:** generalized: Generalized linear models were used to fit the expression counts of each gene. The Cox-Reid adjusted profile likelihood (CR-APL) method was used for estimating the common, trended, and gene-wise dispersions based on approximate conditional likelihood to account for all systematic sources of variations [13]. The APL for the square of the biological coefficient of variation (CV) of the fraction of all cDNA fragments in sample *i* of gene *g* is the penalized log-likelihood,

${APL}_{g}\left( \phi_{g} \right)\mathcal{= l}\left( \phi_{g};\boldsymbol{y}_{g},\hat{\beta}_{g} \right)-\frac{1}{2}\log det\mathcal{I}_{g}$,

where $\boldsymbol{y}_{g}$ are the gene counts for gene *g*, $\hat{\beta}_{g}$ are the estimated coefficients, $\mathcal{l()}$ is the likelihood function, $\mathcal{I}_{g}$ is the Fisher information matrix for gene *g* and $\phi_{g}$ is the square of the CV of the fraction of all cDNA fragments in sample *i* of gene *g* (*36*). In other words, $\phi_{g}$ is the dispersion of gene *g*. The common dispersion can be estimated by maximizing the shared likelihood function,

${APL}_{S}\left( \phi\right)= \frac{1}{G}\sum_{g=1}^{G} {APL}_{g}\left( \phi\right)$,

where ${APL}_{g}$ is the APL of the dispersion $\phi_{g}$. The trended dispersion models $\phi_{g}$ as a smooth function of the average read count of each gene. If the number of genes is greater than 200, bin-wise dispersions will be calculated and a smooth spline will be fitted to each bin. Else, a low-level smooth function will be used to model $\phi_{g}$. The gene-wise dispersion can be estimated by maximizing

${APL}_{g}\left( \phi_{g} \right)+ G_{0}{APL}_{Sg}(\phi_{g})$,

where $G_{0}$ is the weight of the likelihood and ${APL}_{Sg}(\phi_{g})$ is the local shared log-likelihood [13]. To account for other sources of variations, the sex and age of the samples were considered in the design matrix in addition to group information for fitting the models. For each gene, a quasi-likelihood (QL) negative binomial GLM in log form was fitted to the gene counts based on the calculated dispersions and the design matrix. The primary generalized linear model (GLM) in log form can be modelled as,

$log\mu_{gi}= \boldsymbol{x}_{i}^{T}\beta_{g}+logN_{i}$,

where $\boldsymbol{x}_{i}$ is the set of covariates in the design matrix for each sample *I*, and $\beta_{g}$ is the regression coefficient for gene *g* [13].

Dimension reduction and clustering

**ScRNA-sequencing:** post-integration for each tissue, we carried out dimension reduction using uniform manifold approximation and projection (UMAP) and *t*-distributed stochastic neighbour embedding (*t*-SNE) using Seurat to accommodate the non-linearized characteristics of the conventional high-dimensional scRNA-Seq data based on the first 30 batch-corrected Harmony embeddings. Even though an elbow plot of the ranking of Harmony components based on the percentage of variance explained by each component was considered to select for significant embeddings, on average, the first 10 embeddings were selected across tissues using this method; however, by using only the first 10 embeddings, clustering was poorly performed across tissues based on the results and with the different number of clusters performed. Clusters were homogeneously mixed up with one another, which might be due to the inadequacy of information captured by the first 10 Harmony embeddings for such large integrated datasets used. To ensure that more information could be captured, the first 30 batch-corrected Harmony embeddings were chosen. Clustering results significantly improved, and clusters were segregated from each other even with different clustering numbers tested. For each tissue, graph-based clustering was performed using Seurat by constructing a k-nearest neighbors (k-NN) graph followed by a shared nearest neighbor (SNN) modularity optimization clustering procedure using the original Louvain algorithm [2].

**ScATAC-Sequencing:** dimension reduction of scATAC-Seq was carried out based on the corrected LSI embeddings using Seurat. 2nd to 30th corrected LSI embeddings were used, as the first LSI component has a high correlation with sequencing depths (close to an absolute correlation of 1) compared to less (< 0.5) or no correlations of other LSI components with sequencing depths, suggesting that rather than biological variation captured, technical variations were captured, and therefore the first component was skipped. Dimension reduction was done for both UMAP and *t*-SNE. Similar to scRNA-Seq, graph-based clustering was performed using k-NN and SNN, followed by a smart local moving (SLM) algorithm for optimization of community detection in large networks.

**ScImmune-profiling:** only multi-modal scImmune-profiling samples possessing multi-dimensional scRNA-Seq data were required for dimension reduction and clustering procedures. Similar to the procedure mentioned for the scRNA-Seq data, UMAP and *t*-SNE were performed based on the first 30 batch-corrected Harmony embeddings. Clustering was performed using k-NN and SNN followed by optimization of modularity using the original Louvain algorithm.

**Spatial transcriptomics:** dimension reductions using UMAP and *t*-SNE were carried out using the first 30 PCA components, and similar to scRNA-Seq, clustering was performed using k-NN and SNN followed by optimization of modularity using the original Louvain algorithm.

**CyTOF:** dimension reductions using UMAP and *t*-SNE were carried out using the first 30 corrected PCA components using the integrated assay constructed using Seurat, and similar to scRNA-Seq, clustering was performed using k-NN and SNN followed by optimization of modularity using the original Louvain algorithm.

**Flow cytometry:** dimension reductions using UMAP and *t*-SNE were carried out using the first 30 Harmony components, and similar to scRNA-Seq, clustering was performed using k-NN and SNN followed by optimization of modularity using the original Louvain algorithm.

**RNA-sequencing:** PCA components were pre-computed based on the number of highly variable genes present in the dataset. Dimension reductions using UMAP and *t*-SNE were carried out using the first 30 PCA components, and similar to scRNA-Seq, clustering was performed using k-NN and SNN followed by optimization of modularity using the original Louvain algorithm.

Differential expression analysis and celltype annotation

**ScRNA-sequencing:** To identify DEGs in each cell cluster, for each gene in each cluster, the Wilcoxon rank-sum test was carried out between cells in that particular cluster and cells present in all other clusters to determine if the expression of the gene was significantly expressed. P values were corrected for multiple testing using Bonferroni. A threshold was set for each comparison to require the gene to be detected at a minimum of 0.1 in proportion in either of the two groups of cells. A cut-off of at least 0.5 absolute average log-fold change was needed for performing the statistical test between the two groups of cells. Genes passing a threshold of Bonferroni corrected P < 0.01 were retained. Based on the DEG signatures, cell types were manually annotated based on knowledge and the literature. Cell-type level differential expression analyses were performed to identify DEGs in each cell type based on the annotation.

**ScATAC-sequencing:** To identify differentially expressed accessible regions between cell clusters, a differential accessibility (DA) test was carried out using logistic regression followed by a likelihood ratio test [5]. Peak counts per cell were used as an underlying latent variable for the reduction of the sequencing depth effect. A threshold was set for each comparison to require the peak to be detected at a minimum of 0.25 in proportion in either of the two groups of cells. A cut-off of at least 0.25 absolute average log-fold change was needed for performing the statistical test between the two groups of cells. Peaks passing a threshold of Bonferroni corrected P < 0.05 were retained. To interpret the top peaks in each cluster across tissues, the closest gene to each of the peaks was identified based on the positions of the top peaks.

**ScImmune profiling:** in the multi-modal samples, similar to scRNA-Seq, the same procedure and settings were carried out on scRNA-Seq samples to identify DEGs between cell clusters. Genes passing a threshold of Bonferroni corrected P < 0.01 were retained. Based on the DEG signatures, cell types were manually annotated based on knowledge, literature, and the T and B immune repertoire information mapped onto T and B cells.

**Spatial transcriptomics:** Similar to scRNA-Seq data, the same procedure and settings were carried out in spatial transcriptomics to identify DEGs between cell clusters. Genes passing a threshold of Bonferroni corrected P < 0.05 were retained. Based on the DEG signatures, cell types were manually annotated based on knowledge and the literature, as well as automated cell annotation using the Human Primary Cell Atlas as a reference [14].

**CyTOF:** Similar to scRNA-Seq data, the same procedure and settings were carried out in CyTOF samples to identify DEGs between cell clusters. Genes passing a threshold of Bonferroni corrected P < 0.05 were retained. Based on the DEG signatures, cell types were manually annotated based on knowledge and the literature.

**Flow cytometry:** Similar to scRNA-Seq data, the same procedure and settings were carried out in CyTOF samples to identify DEGs between cell clusters. Genes passing a threshold of Bonferroni corrected P < 0.05 were retained.

**RNA-Seq:** To identify DEGs of each tissue compared to all other tissues, DE gene-wise hypothesis testing was carried out for each gene using the QL F test based on the fitted QL negative binomial GLM (*33*), adjusted by the set of covariates in the design matrix. This process accounts for the discovery of DEGs in a single tissue. The process is repeated for every tissue by setting the group information in the design matrix to G1 for the tissue intended for DE testing and G2 for all other tissues in the dataset. To adjust for multiple testing, p values were BH FDR corrected, and genes with BH FDR < 0.05 were chosen as DEGs for the tissues.

Enrichment analyses

For DEGs of scRNA-Seq, multi-modal scImmune-Profiling and RNA-Seq, enrichment analyses [15-18] for each cell type in each tissue (scRNA-Seq and scImmune-Profiling) or each sample (RNA-Seq) were carried out to identify enriched terms and pathways. The top 200 genes for each set ranked by log-fold-change were used for the analyses.

Inference of cell regulatory networks

**ScRNA-sequencing:** Regulon activities in cell types of all tissues were assessed using SCENIC [19] to infer and construct gene regulatory networks. For each tissue, soft gene filtering was carried out by retaining genes with expression counts > 3 in at least 1% of the total cells and genes that were detected in at least 1% of the cells. Subsampling was done to reduce the computational complexity. Correlation analysis was carried out to segregate activation and repression targets. Genie3 was used to infer potential transcription factor targets based on the gene expression data in each cell type across tissues using the Random Forest approach [20]. Regulon activity scores in terms of AUROC metric values and regulon specificity score (RSS) [21] calculated via the Jensen-Shannon (JS) divergence, were obtained for all cell types across tissues. Regulons with AUROC scores > 0.1 and RSS > 0.1 were retained. To maximize the difference across cell types and to reduce potential technical biases, activity scores were binarized, Euclidean distances between retained regulons in cell types of each tissue were calculated, and a complete linkage method was used for hierarchical clustering. Based on the average expression of the regulon obtained for each cell type, the sum of squares (WSS) was obtained based on k-means clustering, and an elbow plot of the number of clusters against its corresponding WSS was drawn and used to determine an appropriate number of regulon modules for each tissue.

Cell-cell communications

**ScRNA-sequencing:** For the construction of intercellular signal networks, we utilized CellPhoneDB [22] to infer cell-to-cell communications mediated by ligand-receptor complexes. Using the CellPhoneDB ligand-receptor repository as a reference, each tissue was first subsampled using geometric sketching to reduce time and computational complexities. Pairwise cell type to cell type comparisons was made within each tissue based on the expression of each receptor in one cell type and the expression of each ligand in the other cell type. Receptor-ligand pairs with significant means (p < 0.05) denoting high cell-type specificity, significant mean > 25th quantile of the overall significant mean values and rank > 25th quantile of the overall ranks were retained.

Immune repertoire analysis

**ScImmune profiling:** We first divided the analysis into two categories, namely, 1) tissue-level T or B repertoire analyses and 2) analysis of the post-annotated T or B cell sub-types mapped with repertoire information (for multi-modal samples). Using scRepertoire, clonotypes were first quantified to assess the number of unique clonotypes present for each category, and their relative abundance was calculated. CDR3 sequence lengths were determined for each category. Within each category, the top clonotypes based on the CDR3 nucleotide sequence, amino acid sequence, VDJC gene sequence, and the combination of CDR3 nucleotide and VDJC gene sequences were obtained and compared across each tissue to observe any similarities in terms of the top clonotypes across tissues. Clonal homeostasis was calculated by categorizing clonotype relative abundance proportions into five clonal groups, namely, single (clonotype with one cell event); small (clonotypes with more than 1 and less than or equal to 5 cell events); medium (clonotypes with more than 5 and less than or equal to 20 cell events); large (clonotypes with more than 20 and less than or equal to 100 cell events) and hyperexpanded (clonotypes with more than 100 and less than or equal to 500 cell events) groups. T or B repertoire gene usages were computed to examine the rearrangement frequency of these genes in diversifying the T and B immune repertoires. Clonal overlapping based on each gene usage was carried out to measure repertoire similarities across samples by computing the level of shared clonotypes and Morisita’s overlap index based on each gene. Spectratyping to look at the top gene segments based on CDR3 lengths was calculated based on CDR3 nucleotide length and amino acid length. K-mer and sequence motif analysis was carried out to examine k-mer positional amino acid proportions and chemical characteristics of the amino acids present at each position. In the atlas, K was chosen based on the median CDR3 length of each repertoire in each tissue. In the search database, the k-mer was calculated from k = 2 to k = 20.

Integrative analysis of omics

**ScRNA** **sequencing:** Integrative analysis was performed on adult tissues with matching fetal tissues to observe phenotypic differences between the two groups at the cell-type level. Samples from these tissues were integrated and dimensionally reduced using UMAP. Cell type annotations were mapped back onto the UMAP coordinates to give an overall representation of cell types present in the tissues. MetaNeighbors were used for the identification of clusters of cell types with high similarities/correlations between fetal and adult tissues. Average gene expression was computed for the DEGs of each cell type in each tissue using Seurat. The top 100 upregulated DEGs for each cell type across all tissues were considered, and similarity matrices were calculated and represented in heatmaps to observe clustering trends in tissues, physiological systems, cell types, and cell type classes. The same similarity matrices were constructed based on the top regulon in each cell type across fetal and adult tissues. Regulons that demonstrated enrichment/activeness with AUROC scores > 0.1 and RSS > 0.1 were selected to construct the similarity matrices. Cell types with AUROC scores > 0.9 in the DEG correlation analysis indicated that high correlation confidence (0 being the lowest and 1 being the highest confidence) was retained for the construction of connectivity maps in terms of Circos plots for both fetal and adult tissues.

**ScATAC-Sequencing:** Top peaks obtained from differential expression analyses across tissues were collated and visualized with Manhattan plots, displaying these top peaks at their respective chromosome positions against negative log P values. The closest genes near the top 10 peaks of each tissue were chosen and are shown in each separate plot.

**ScImmune** **profiling**: For each immune repertoire, the clonotypes of each tissue were compared with one another, and the degree of overlap between tissues was calculated. Hierarchical clustering based on the degree of repertoire overlap was also assessed. For each repertoire, the top 10 clonotypes from each tissue were compared simultaneously with one another to observe similarities among the top clonotypes across tissues. The distance based on VDJ gene usage between all tissues and repertoires was calculated based on JS divergence, multidimensional scaling (MDS), and k-means. Distance based on each specific gene usage between all tissues and repertoires was calculated using the same algorithms. Clonal diversities were estimated based on six different methods: 1) Chao1 estimator, a non-parametric asymptotic estimator for clonal richness; 2) hill numbers; a mathematically unified family of diversity indices; 3) true diversity, the effective number of equally abundant types needed for the average proportional abundance to be equal to observed abundance; 4) Gini-Simpson index, the probability of two entities being different types; 5) Inverse Simpson index, to indicate the effective number of clonotypes; 6) D50, percentage of dominant unique species that made up 50% of the total community [6, 7].

**Integrative scImmune-Profiling with scRNA-Seq:** For multimodal samples, integrative analysis of immune repertoires with their corresponding scRNA-Seq data was done by projecting the clonal homeostasis information of the repertoires onto cells of the corresponding tissues to dissect homeostasis in terms of the cell subtypes. The top 10 clonotypes for each cell subtype were compared across tissues to observe clonal similarities.

**Integrative RNA-sequencing with cis-eQTLs and cis-sQTLs:** The top 50 DEGs from each tissue of RNA-Seq data were chosen and mapped onto the cis-sQTLs and cis-eQTLs of all tissues. Manhattan plots were used to illustrate the relationships of these top DEGs with their corresponding QTLs across all tissues. In other words, the significance level ($-{log}_{10}(p$)) of each sQTLs or eQTLs of the top 50 DEGs of a particular tissue for all tissues was displayed on the same plot to observe the inter-connectedness of DEGs in RNA-Seq with the genetic traits of the same tissue, as well as across tissues. The top 10 matching RNA-Seq DEGs that were also highly significant for the genes of top eQTLs or sQTLs (ranked from the highest $-{log}_{10}(p$) are labeled. To serve as a form of comparison, the top 10 sQTLs or eQTLs of each tissue were also subsequently assessed.

**Integration of bulk RNA-Seq with pseudobulk scRNA-Seq:** Taking the scRNA-Seq data with manually annotated cell types, the average expression in raw read counts (produced after quantification) of each gene of each tissue and cell type was obtained. For RNA-Seq data, raw read counts (produced after quantification) were retrieved from the GTEx data portal [23] and subsequently log-normalized with a scale factor of 1 M, and the average expression in raw read count form of each gene of each tissue was obtained. Using Seurat integration, the two datasets underwent integration. HV genes that varied across samples of bulk and scRNA-Seq were selected and identified as integration features. Dimension reduction based on Reciprocal PCA was performed to identify mutual nearest neighbors from each dataset, in other words, to identify a set of anchors that can be used for integration. Integration was performed based on the anchors and z score transformation. PCA embeddings for the integrated dataset were calculated based on the HV genes present in the integrated dataset. Dimension reduction to 3D space was carried out using both UMAP and *t*-SNE using the first 30 PCA components.

Website construction

The website consists of two main sections: 1) multi-tissues phenotypic characteristics across different omics to provide insights in a multiomics perspective and 2) database summarizing phenotypic discoveries for multiomics query. The website is hosted by Wix, and its database queries are hosted by RShiny and Github. The website was constructed mainly based on R, PHP, HTML, JS, and CSS.

**References**

1. DePasquale EAK, Schnell DJ, Van Camp PJ, Valiente-Alandí Í, Blaxall BC, Grimes HL, Singh H, Salomonis N: **DoubletDecon: Deconvoluting Doublets from Single-Cell RNA-Sequencing Data.** *Cell Rep* 2019, **29:**1718-1727.e1718.

2. Hao Y, Hao S, Andersen-Nissen E, Mauck WM, 3rd, Zheng S, Butler A, Lee MJ, Wilk AJ, Darby C, Zager M, et al: **Integrated analysis of multimodal single-cell data.** *Cell* 2021, **184:**3573-3587.e3529.

3. Fang R, Preissl S, Li Y, Hou X, Lucero J, Wang X, Motamedi A, Shiau AK, Zhou X, Xie F, et al: **Comprehensive analysis of single cell ATAC-seq data with SnapATAC.** *Nature Communications* 2021, **12:**1337.

4. Li H, Handsaker B, Wysoker A, Fennell T, Ruan J, Homer N, Marth G, Abecasis G, Durbin R, Subgroup GPDP: **The Sequence Alignment/Map format and SAMtools.** *Bioinformatics* 2009, **25:**2078-2079.

5. Stuart T, Srivastava A, Madad S, Lareau CA, Satija R: **Single-cell chromatin state analysis with Signac.** *Nature Methods* 2021, **18:**1333-1341.

6. Borcherding N, Bormann NL, Kraus G: **scRepertoire: An R-based toolkit for single-cell immune receptor analysis.** *F1000Res* 2020, **9:**47.

7. Nazarov V: **immunarch. bot, and Eugene Rumynskiy. 2020. immunomind/immunarch: 0.6. 5: Basic single-cell support.** Zenodo; 2020.

8. Weisenfeld NI, Kumar V, Shah P, Church DM, Jaffe DB: **Direct determination of diploid genome sequences.** *Genome Res* 2017, **27:**757-767.

9. Hahne F, LeMeur N, Brinkman RR, Ellis B, Haaland P, Sarkar D, Spidlen J, Strain E, Gentleman R: **flowCore: a Bioconductor package for high throughput flow cytometry.** *BMC Bioinformatics* 2009, **10:**106.

10. Finak G, Frelinger J, Jiang W, Newell EW, Ramey J, Davis MM, Kalams SA, De Rosa SC, Gottardo R: **OpenCyto: An Open Source Infrastructure for Scalable, Robust, Reproducible, and Automated, End-to-End Flow Cytometry Data Analysis.** *PLOS Computational Biology* 2014, **10:**e1003806.

11. Robinson MD, McCarthy DJ, Smyth GK: **edgeR: a Bioconductor package for differential expression analysis of digital gene expression data.** *Bioinformatics* 2009, **26:**139-140.

12. Korsunsky I, Millard N, Fan J, Slowikowski K, Zhang F, Wei K, Baglaenko Y, Brenner M, Loh P-r, Raychaudhuri S: **Fast, sensitive and accurate integration of single-cell data with Harmony.** *Nature Methods* 2019, **16:**1289-1296.

13. McCarthy DJ, Chen Y, Smyth GK: **Differential expression analysis of multifactor RNA-Seq experiments with respect to biological variation.** *Nucleic Acids Res* 2012, **40:**4288-4297.

14. Mabbott NA, Baillie JK, Brown H, Freeman TC, Hume DA: **An expression atlas of human primary cells: inference of gene function from coexpression networks.** *BMC Genomics* 2013, **14:**632.

15. Ritchie ME, Phipson B, Wu D, Hu Y, Law CW, Shi W, Smyth GK: **limma powers differential expression analyses for RNA-sequencing and microarray studies.** *Nucleic Acids Research* 2015, **43:**e47-e47.

16. Yu G, Wang LG, Yan GR, He QY: **DOSE: an R/Bioconductor package for disease ontology semantic and enrichment analysis.** *Bioinformatics* 2015, **31:**608-609.

17. Wu T, Hu E, Xu S, Chen M, Guo P, Dai Z, Feng T, Zhou L, Tang W, Zhan L, et al: **clusterProfiler 4.0: A universal enrichment tool for interpreting omics data.** *Innovation (Camb)* 2021, **2:**100141.

18. Yu G, He QY: **ReactomePA: an R/Bioconductor package for reactome pathway analysis and visualization.** *Mol Biosyst* 2016, **12:**477-479.

19. Aibar S, González-Blas CB, Moerman T, Huynh-Thu VA, Imrichova H, Hulselmans G, Rambow F, Marine J-C, Geurts P, Aerts J, et al: **SCENIC: single-cell regulatory network inference and clustering.** *Nature Methods* 2017, **14:**1083-1086.

20. Huynh-Thu VA, Irrthum A, Wehenkel L, Geurts P: **Inferring Regulatory Networks from Expression Data Using Tree-Based Methods.** *PLOS ONE* 2010, **5:**e12776.

21. Suo S, Zhu Q, Saadatpour A, Fei L, Guo G, Yuan GC: **Revealing the Critical Regulators of Cell Identity in the Mouse Cell Atlas.** *Cell Rep* 2018, **25:**1436-1445.e1433.

22. Efremova M, Vento-Tormo M, Teichmann SA, Vento-Tormo R: **CellPhoneDB: inferring cell–cell communication from combined expression of multi-subunit ligand–receptor complexes.** *Nature Protocols* 2020, **15:**1484-1506.

23. **The GTEx Consortium atlas of genetic regulatory effects across human tissues.** *Science* 2020, **369:**1318-1330.
