# Supplementary material for: Single Cell Atlas: a single-cell multi-omics human cell encyclopedia
Source: Genome Biol. 2024 Apr 19;25:104. doi: 10.1186/s13059-024-03246-2 (PMC11027364; doi:10.1186/s13059-024-03246-2)
Supplement: Supplementary file 4 — Additional file 4. Review history. [file 13059_2024_3246_MOESM4_ESM.docx]

Review History

**First round of review**

**Reviewer 1**

**Were you able to assess all statistics in the manuscript, including the appropriateness of statistical tests used?**

No

**Comments to author:**

The authors collected single-cell and bulk multi-omics data from multiple human tissues and build a resource named SingleCellAtlas. The atlas presented here is different from previous single cell atlases is because it cover multiple omics data type and multiple tissues. I believe this resource will be useful for many research projects. However, I have some concerns about the manuscript.

1. Allowing user to download the raw data, processed data, and results would help to increase the impact of this research. I strongly encourage author to provide a download link. Batch download like ENCODE project is more appreciated.

2. An Experiment matrix, like ENCODE project, will be helpful for user to find the tissue and data type (https://www.encodeproject.org/matrix/?type=Experiment&control_type!=*&status=released&perturbed=false). People may be interested in some combination of omics data, like scRNA-seq + scATAC-seq. Experiment matrix clearly shows these data are available on which tissues.

3. Some researchers may be interested in matched omics data on the same individuals. It is better to provide a unique id for each individual so that it can be used to map among omics datasets.

4. A description of the original source of the data is missing. I didn't find the "Key Resources Table" mentioned in page 19.

5. The "AUROC > 0.1" ( page 4, line 43) is not clear. AUROC of what? What is the label? What is the score? A higher AUROC reflect what?

6. How author perform peak calling in scATC-seq is not clear. The scATAC-seq peak in table is 50k, which is too broad, and thus hard to be used in regulatory analysis.

7. The section of "scATAC-Sequencing analysis of genotype-phenotype associations in tissue cell clusters" is not clear. Especially "Among the significant disease associations, SNP**, are in high-risk association with the cause of death by motor neuron disease".

**Reviewer 2**

**Were you able to assess all statistics in the manuscript, including the appropriateness of statistical tests used?**

**Yes:** The appropriateness of statistical tests is discussed in the comments to the authors.

**Comments to author:**

In this manuscript by the Single Cell Atlas consortium, the authors present a database integrating single-cell multi-omics datasets across human adult and fetal healthy tissues. This appears to in principle represent the largest collection of processed and more readily accessible single-cell data, covering a large breath of human tissues. The authors use this database to showcase various integrative analyses of the diverse phenotypic variations across omics layers and tissues. Complementarily, the authors provide a web-based resource to facilitate the interactive visualization and exploration of the data. Overall, this is a valuable database aimed at facilitating the access of curated single-cell data to the research community.

However, this reviewer has concerns and suggestions about the curation of the data, applied quality control, the benefits and limitations of the database compared to existing ones, and the metadata annotation. The reviewer would also recommend enhancing the presented study cases, as they could be more meaningful. The value and relevance of comparing TCR sequences between donors are also unclear. Overall, the presented examples provide a rather limited showcase of the full potential and utility of the database.

Major comments
1. The database integrates both adult and fetal human tissues utilizing multi-omic data and resources such as this one will be hugely beneficial for the single-cell community to navigate the ever-growing landscape of publicly available datasets. However, existing databases such as the single cell portal (https://singlecell.broadinstitute.org/single_cell), the Human Cell Atlas (https://data.humancellatlas.org/), or GTEX (https://gtexportal.org/home/singleCellOverviewPage) provide similar functions and data modalities. Can the authors highlight distinguishing features, advantages, and limitations of their database more clearly (e.g., in form of a table) and do they foresee any integration of these multiple orthogonal efforts?

2. The number of cells included in the analysis is inconsistent/confusing in both the paper and the website. Ultimately, only the cells meeting the respective quality control (QC) criteria will likely be used by the research community and should therefore be the ones that are primarily highlighted.

3. Related to the previous point, the authors should provide QC plots/information for all relevant data for the reader to get a sense of how the applied thresholds (e.g., maximum 30,000 number of peaks for scATAC-seq) or exclusion of thresholds (e.g., TSS enrichment was not used?) affect filtering. This may in fact also vary from tissue to tissue depending on the experimental tissue processing and resulting quality. Also, the filtering of cells with a mitochondrial content > 5% in the single-cell RNA-seq datasets might be too strict for many human tissues with higher physiologic mitochondrial content (e.g., heart) and this threshold probably makes a significant difference depending on whether nuclei or cells were used for the transcriptomic profiling. As such, QC metrics might need to be adjusted depending on the tissue of origin and experimental setup. In general, we are concerned about the very high proportion of cells discarded after applying used thresholds (Introduction: We profiled 66,863,102 cells from scRNA-Seq, Results: After filtering, 2,866,972 high-quality cells were obtained for scRNA-Seq). As less than 5% of cells remain after filtering, this appears very unusual compared to what is typically reported in the literature.

4. The paper is, unfortunately, lacking metadata. There is no information about the number of donors contributing to the different datasets, while it highly preconditions the representativity of the results and will certainly be a legitimate concern for the users. This information is particularly crucial while comparing the T cells/B cells clonotypes. For each single-cell modality and tissue, the authors should provide a UMAP where the different individuals are projected and a plot summarizing each individual's relative contribution to the pool of cells. They should also include a general plot indicating the number of individuals contributing to each tissue for each modality and provide a supplementary table with basic donor data (e.g., age, sex), or at the very least, the link to the metadata page on their website.

5. The authors make an indistinctive use of t-SNE and UMAP 2D/3D visualizations. For the sake of consistency and because this is an integrative resource, this reviewer recommends using one of the two methods for all the figures.

6. The authors implement the Harmony algorithm to correct inter-dataset batch effects. However, they do not take into account the potential batch effects existing within each data source. The authors should therefore provide a UMAP colored by data source in order to assess the potential intra-dataset batch effects.

7. Multiple testing corrections were processed using both Bonferroni and Benjamini-Hochberg methods without further clarification on why these two methods were used indistinctly. As for comment 5, the authors should be consistent in their methodology throughout the manuscript.

8. The authors should provide use cases demonstrating the usefulness of the database. Can users integrate their own data with the provided multimodal data? Is there any workflow they could implement to do so?

9. Single-cell research is a rapidly evolving field, continually including new methods to generate or analyze data. The authors did not clarify how the database will be maintained and updated. Is it planned to integrate other omics modalities, analyses, and datasets in the future?

10. After all, flow cytometry and CyTOF datasets were of limited value. While the authors should discuss the consideration of such data, their advertising in the introduction and in the results section is not warranted, as also no such data is accessible on the website right now.

11. Overall, the level of granularity of cell type/state annotation (330 cell types) appears rather limited. Similar and comparatively less comprehensive efforts already identify up to 657 subtypes DOI: 10.1126/science.aba7721. Now, this reviewer understands the immense complexity of harmonizing cell type and state annotation, which should also be thoroughly discussed by the authors. The current categorization of the annotated 330 cell types into 17 major cell type classes and comparisons thereof is a vast oversimplification of the actual biological complexity, whereas resolving this complexity, should be the major emphasis of these and other single-cell databases.

Minor comment
1. Pseudotime is a complex concept that, despite its name, should not be confused with real-time. Therefore, the authors should not mention terms such as "earlier" transitions.

**Authors Response**

**Point-by-point responses to the reviewers’ comments:**

**Response to the editor and the reviewers**

**Additional comments from the Chief Editor:**

As you will see from the reports, both referees are broadly favorable and find the work of potential interest, but they raise important issues that we must ask you to address, in the form of a revised manuscript, before we reach a final decision on publication. In particular, it seems to us to be essential that both Referees' concerns about the lack of (complete) metadata are resolved. Referee 2 has also requested more transparency on the QC process, intra-dataset batch corrections and a more uniform use of statistics and plots. Both referees have made suggestions to increase the functionality of your database such as separate download function and demonstration of how to integrate one’s own dataset. Referee 1 also wants more clarity on the dataset sources, in addition to adhering to our mandatory open data policies (see also below), and in line with the question by Reviewer #2 on updating and maintaining your database, we recommend that you provide a timestamped database content with accession numbers. Please ensure that these and all other issues raised by the referees are addressed in full.

**Response:**

Thank you for your feedback and for providing a summary of the reviewers' comments on our manuscript. We greatly appreciate the overall positive reception of our work and the recognition of its potential interest. We fully acknowledge the importance of addressing the concerns raised by both referees before reaching a final decision on publication.

Regarding the concerns about the lack of complete metadata, we understand the significance of providing comprehensive information to enable reproducibility and further exploration of the data. We have taken these concerns to heart and have made substantial efforts to enhance the metadata accompanying our datasets. In the revised manuscript and on the website, we have included a more detailed description of the metadata for each dataset, ensuring that key information such as sample attributes, universal keys to each sample and project, references to the projects such as their relevant literature and web link, as well as accession numbers are fully available.

We appreciate the request of Referee 2 for more transparency regarding the quality control (QC) process and intra-dataset batch corrections. In response, we have constructed a web page displaying before and after batch correction visualizations across tissues to allow a more comprehensive overview of the batch correction process to ensure data integrity. We believe that these additions will greatly improve the transparency and usability of our resource. We have also provided explanations for our QC procedures, including the specific metrics and thresholds employed. We believe that these clarifications will address the concerns raised by Referee 2 and provide a more robust framework for data analysis.

Furthermore, we have carefully considered the suggestions made by both referees to enhance the functionality of our database. We have implemented a separate download function, allowing users to easily access and integrate our data into their own analyses. Additionally, we are working on another project to utilize the datasets present in the SingleCellAtlas (SCA) to enable integrate their own datasets with our resources, and the project is still under development. Once the project is published, we will link up the two websites and not only could the users explore the datasets interactively, but also could conduct comprehensive and vibrant analyses to produce results covering a wide range of functionalities.

Regarding the concerns raised by Referee 1 regarding dataset sources and adherence to mandatory open data policies, we have revised the manuscript to provide greater clarity on the sources of our datasets in Supplementary Table 8 as well as on the website under the Download tab, with an expression matrix showing the information across omics and datasets, as well as the Source tab explicitly displaying the references to each data source. As the datasets were collected from open-access repositories, we have also ensured the compliance with open data policies by providing their accession numbers for the current datasets. Furthermore, we provided a timestamped database content with accession numbers to facilitate traceability and future updates. The current version of the database is SCA V1.0.0 and we will conduct periodical updates and update the version number depending on the degree of update (i.e., if there is a minor or small update, the version will be V1.0.1 or V1.1.0 and will list down the changes made in the new versions in point form on the website).

**Reviewer #1**

Comments for the Author

The authors collected single-cell and bulk multi-omics data from multiple human tissues and build a resource named SingleCellAtlas. The atlas presented here is different from previous single cell atlases is because it cover multiple omics data type and multiple tissues. I believe this resource will be useful for many research projects. However, I have some concerns about the manuscript.

**Response:**

Thank you very much for your thoughtful comments and great suggestions and we are appreciative of your acknowledgment of our work. We have made significant efforts according to your concerns and suggestions, which have extensively strengthened our manuscript and enhanced the overall impact of our resource. These included an incorporation of interactive expression matrix to have an overview of the tissues across omics in both adult and fetal groups. Besides, we provided a batch download function by simply select the tissues or omics of interest and the links to the associated files will be batch retrieved for the users. Besides, we incorporated unique sample and project identity numbers universally across the omics. We have also addressed your concerns with regards to the broad range of peaks in the single-cell ATAC-Sequencing (scATAC-Seq) results. Explicitly, we have responded your comments point-by-point as listed below.

1. Allowing user to download the raw data, processed data, and results would help to increase the impact of this research. I strongly encourage author to provide a download link. Batch download like ENCODE project is more appreciated.

**Response:**

Thank you so much for the great suggestion. We fully agree that making the data and results available for download would increase the impact and usefulness of our resource to the research community. We have now provided a data selection page for batch download of raw and processed data on our atlas platform and can be accessible at [www.singlecellatlas.org/download.](http://www.singlecellatlas.org/download) Currently, the download page is protected (password to access the page: *gbreviewers*) and will be made publicly accessible once published. By clicking on the tissues/omics/age groups/etc. of interest, download links to all relevant files will be compiled in a text readable .txt file and can be downloaded for batch download after clicking on the Download button as shown in **Figure R1**. We appreciate your suggestion for making this happen. To add on, most data files were stored in a R readable *.RDS* format in a Seurat object [1], and both the raw and processed data can be retrieved from the *.RDS* files. We did not include the download for raw sequencing files such as *.fastq* format files, due to the excessive large storage space. Instead, we provide web links to the published paper or to their websites where raw data can be retrieved from. This information can be found under the data Source page [(www.singlecellatlas.org/source](http://www.singlecellatlas.org/source)).

2. An Experiment matrix, like ENCODE project, will be helpful for user to find the tissue and

data	type
[(https://eur01.safelinks.protection.outlook.com/?url=https%3A%2F%2Fwww.encodeproject.o](https://eur01.safelinks.protection.outlook.com/?url=https%3A%2F%2Fwww.encodeproject.org%2Fmatrix%2F%3Ftype%3DExperiment%26control_type!%3D*%26status%3Dreleased%26perturbed%3Dfalse&data=05%7C01%7Cxuexin.li%40ki.se%7Cd5a376022d244153af2108db3107e2d9%7Cbff7eef1cf4b4f32be3da1dda043c05d%7C0%7C0%7C638157682165608180%7CUnknown%7CTWFpbGZsb3d8eyJWIjoiMC4wLjAwMDAiLCJQIjoiV2luMzIiLCJBTiI6Ik1haWwiLCJXVCI6Mn0%3D%7C3000%7C%7C%7C&sdata=xJ1EBuSbfgMuAysD3JGIF693s2CZuShO%2FfaOI1zv8iY%3D&reserved=0) [rg%2Fmatrix%2F%3Ftype%3DExperiment%26control_type!%3D*%26status%3Dreleased%](https://eur01.safelinks.protection.outlook.com/?url=https%3A%2F%2Fwww.encodeproject.org%2Fmatrix%2F%3Ftype%3DExperiment%26control_type!%3D*%26status%3Dreleased%26perturbed%3Dfalse&data=05%7C01%7Cxuexin.li%40ki.se%7Cd5a376022d244153af2108db3107e2d9%7Cbff7eef1cf4b4f32be3da1dda043c05d%7C0%7C0%7C638157682165608180%7CUnknown%7CTWFpbGZsb3d8eyJWIjoiMC4wLjAwMDAiLCJQIjoiV2luMzIiLCJBTiI6Ik1haWwiLCJXVCI6Mn0%3D%7C3000%7C%7C%7C&sdata=xJ1EBuSbfgMuAysD3JGIF693s2CZuShO%2FfaOI1zv8iY%3D&reserved=0) [26perturbed%3Dfalse&data=05%7C01%7Cxuexin.li%40ki.se%7Cd5a376022d244153af210](https://eur01.safelinks.protection.outlook.com/?url=https%3A%2F%2Fwww.encodeproject.org%2Fmatrix%2F%3Ftype%3DExperiment%26control_type!%3D*%26status%3Dreleased%26perturbed%3Dfalse&data=05%7C01%7Cxuexin.li%40ki.se%7Cd5a376022d244153af2108db3107e2d9%7Cbff7eef1cf4b4f32be3da1dda043c05d%7C0%7C0%7C638157682165608180%7CUnknown%7CTWFpbGZsb3d8eyJWIjoiMC4wLjAwMDAiLCJQIjoiV2luMzIiLCJBTiI6Ik1haWwiLCJXVCI6Mn0%3D%7C3000%7C%7C%7C&sdata=xJ1EBuSbfgMuAysD3JGIF693s2CZuShO%2FfaOI1zv8iY%3D&reserved=0) [8db3107e2d9%7Cbff7eef1cf4b4f32be3da1dda043c05d%7C0%7C0%7C6381576821656081](https://eur01.safelinks.protection.outlook.com/?url=https%3A%2F%2Fwww.encodeproject.org%2Fmatrix%2F%3Ftype%3DExperiment%26control_type!%3D*%26status%3Dreleased%26perturbed%3Dfalse&data=05%7C01%7Cxuexin.li%40ki.se%7Cd5a376022d244153af2108db3107e2d9%7Cbff7eef1cf4b4f32be3da1dda043c05d%7C0%7C0%7C638157682165608180%7CUnknown%7CTWFpbGZsb3d8eyJWIjoiMC4wLjAwMDAiLCJQIjoiV2luMzIiLCJBTiI6Ik1haWwiLCJXVCI6Mn0%3D%7C3000%7C%7C%7C&sdata=xJ1EBuSbfgMuAysD3JGIF693s2CZuShO%2FfaOI1zv8iY%3D&reserved=0) [80%7CUnknown%7CTWFpbGZsb3d8eyJWIjoiMC4wLjAwMDAiLCJQIjoiV2luMzIiLCJBTiI6Ik](https://eur01.safelinks.protection.outlook.com/?url=https%3A%2F%2Fwww.encodeproject.org%2Fmatrix%2F%3Ftype%3DExperiment%26control_type!%3D*%26status%3Dreleased%26perturbed%3Dfalse&data=05%7C01%7Cxuexin.li%40ki.se%7Cd5a376022d244153af2108db3107e2d9%7Cbff7eef1cf4b4f32be3da1dda043c05d%7C0%7C0%7C638157682165608180%7CUnknown%7CTWFpbGZsb3d8eyJWIjoiMC4wLjAwMDAiLCJQIjoiV2luMzIiLCJBTiI6Ik1haWwiLCJXVCI6Mn0%3D%7C3000%7C%7C%7C&sdata=xJ1EBuSbfgMuAysD3JGIF693s2CZuShO%2FfaOI1zv8iY%3D&reserved=0) [1haWwiLCJXVCI6Mn0%3D%7C3000%7C%7C%7C&sdata=xJ1EBuSbfgMuAysD3JGIF693s](https://eur01.safelinks.protection.outlook.com/?url=https%3A%2F%2Fwww.encodeproject.org%2Fmatrix%2F%3Ftype%3DExperiment%26control_type!%3D*%26status%3Dreleased%26perturbed%3Dfalse&data=05%7C01%7Cxuexin.li%40ki.se%7Cd5a376022d244153af2108db3107e2d9%7Cbff7eef1cf4b4f32be3da1dda043c05d%7C0%7C0%7C638157682165608180%7CUnknown%7CTWFpbGZsb3d8eyJWIjoiMC4wLjAwMDAiLCJQIjoiV2luMzIiLCJBTiI6Ik1haWwiLCJXVCI6Mn0%3D%7C3000%7C%7C%7C&sdata=xJ1EBuSbfgMuAysD3JGIF693s2CZuShO%2FfaOI1zv8iY%3D&reserved=0) [2CZuShO%2FfaOI1zv8iY%3D&reserved=0](https://eur01.safelinks.protection.outlook.com/?url=https%3A%2F%2Fwww.encodeproject.org%2Fmatrix%2F%3Ftype%3DExperiment%26control_type!%3D*%26status%3Dreleased%26perturbed%3Dfalse&data=05%7C01%7Cxuexin.li%40ki.se%7Cd5a376022d244153af2108db3107e2d9%7Cbff7eef1cf4b4f32be3da1dda043c05d%7C0%7C0%7C638157682165608180%7CUnknown%7CTWFpbGZsb3d8eyJWIjoiMC4wLjAwMDAiLCJQIjoiV2luMzIiLCJBTiI6Ik1haWwiLCJXVCI6Mn0%3D%7C3000%7C%7C%7C&sdata=xJ1EBuSbfgMuAysD3JGIF693s2CZuShO%2FfaOI1zv8iY%3D&reserved=0)). People may be interested in some combination of omics data, like scRNA-seq + scATAC-seq. Experiment matrix clearly shows these data are available on which tissues.

**Response:**

Thank you so much for the valuable suggestion and providing such a great idea in helping us to improve the website. We have now added an experiment matrix to help users to find the tissue and the data type they are interested in. As shown in **Figure R1**, the matrix can be found in the Download page [(www.singlecellatlas.org/download)](http://www.singlecellatlas.org/download). We provided as many selection or filtering criteria to help users to identify the data or tissue type that they are interested in, and as mentioned, after selection, the data can be readily and easily retrieved via batch download. We will made further improvements in the next version of the database by providing more information for each project or sample to the users.

**Figure R1.** Screenshot of the download page containing expression matrix as well as data selection options for batch download of selected datasets.

3. Some researchers may be interested in matched omics data on the same individuals. It is better to provide a unique id for each individual so that it can be used to map among omics datasets.

**Response:**

Thank you very much for your insightful and thoughtful suggestion. We have now added universal unique ids for each sample and project across omics types so that it can be mapped among various omics datasets. This information can be retrieved from the metadata of the Seurat objects stored in each downloadable datasets, with each cell or sample labeled with both sample (under column name *SCA_SID*) and project (under column name *SCA_PID*) unique ids.

4. A description of the original source of the data is missing. I didn't find the "Key Resources Table" mentioned in page 19.

**Response:**

Thank you for the careful inspection. The ‘Key Resources Table’ was a table constructed to indicate all the data sources (with relevant literature titles, original data accession, and weblinks to the original data source) used for the construction of the SingleCellAtlas. We have

now replaced the phrase ‘Key Resources Table’ with ‘Table S8’ and added the supplementary Table S8 to the manuscript.

5. The "AUROC > 0.1" ( page 4, line 43) is not clear. AUROC of what? What is the label? What is the score? A higher AUROC reflect what?

**Response:**

Thank you so much for noticing this. AUROC stands for area under the receiver operating characteristic curve (AUROC) or simply, area under the curve (AUC). This is a scoring system (ranges from 0 to 1) calculated based on the AUCell algorithm [2], to represent the activity of the regulons in a cell, such that, the higher the AUROC value, the more enriched is the regulon at the top genes in the cell, which the top genes are ranked by decreasing expression values [2]. Hereby, we calculated the activity of the regulons for each cell type and retained regulons with an activity of AUROC > 0.1. We have also added clarification to the abbreviation ‘AUROC’ in the manuscript on page 4, line 43.

6. How author perform peak calling in scATC-seq is not clear. The scATAC-seq peak in table is 50k, which is too broad, and thus hard to be used in regulatory analysis.

**Response:**

Thank you so much for the comment and for noticing the broad range in the peaks. For scATAC-Seq data, unfortunately, many papers did not provide a full dataset, such as lack of fragment data. For instance, a typical 10X scATAC-Seq data Cell Ranger output consists of input files such as the fragment or peak file as shown in the following link[:https://www.10xgenomics.com/resources/datasets/10k-human-pbmcs-atac-v2-](https://www.10xgenomics.com/resources/datasets/10k-human-pbmcs-atac-v2-chromium-controller-2-standard)

[chromium-controller-2-standard,](https://www.10xgenomics.com/resources/datasets/10k-human-pbmcs-atac-v2-chromium-controller-2-standard) yet many failed to provide such information, especially for fragment files, and most of them only contained the ‘peak by cell matrix’ files. This is restricting our analysis for scATAC-Seq to a great extent such as TSS enrichment step cannot be performed due to the lack of fragment data, not to say to infer gene activity and carry out cell type annotations. Therefore, unlike other omics which in most cases contained a complete input data for downstream analysis, analysis of scATAC-Seq was rather limited. However, in the future versions of the database, while populating the datasets, we will have more datasets will complete information to showcase a more vibrant and less limited series of analysis outputs.

As for the peak range, we totally agreed with you as it is also a concern for us. As we were merging different databases to construct an integrative analysis of the scATAC-Seq data, which were conducted based on first merging the common peaks between samples [3], and for the datasets we collected, this substantially reduced the number of peaks left after incorporating more samples, and the remaining common peaks were only at a few hundred for some tissue types. As such, during the merging process (i.e., merging was done without considering just the common peaks, but all post-quality control peaks), the software Signac [3] that we used for scATAC-Seq analysis will combine some peaks to form a large peak range as you have seen in the manuscript. To resolve problems you have pointed out, we decided to utilize a sample-wise approach by analyzing each sample separately to avoid lost of information during merging due to less commonly shared peaks. Such samplewise results were updated in the database website under the scATAC-Seq page for all available tissues, as well as in the peak search query and kept only peaks with <=5000 range.

7. The section of "scATAC-Sequencing analysis of genotype-phenotype associations in tissue cell clusters" is not clear. Especially "Among the significant disease associations, SNP**, are in high-risk association with the cause of death by motor neuron disease".

**Response:**

Thank you for the invaluable comment. After considering also the broad range of the significant peaks, we filtered out those with > 5,000 peak range and only around 20 associations past the criteria. Especially when input files such as fragment files were missing in many data sources, making the cell type annotation and identification difficult. As such, we decided to employ a samplewise approach for running each sample of the tissues individual and display their clustering patterns, differentially expressed peaks, as well as closest genes in these peaks of each cluster on the website instead, and we removed any conclusions drawn from the broad peak and integrated sample results as the case study input data for scATAC-Seq is comparatively limited as compared to omics such as scRNA-Seq and spatial transcriptomics. Nonetheless, as our purpose is to construct a large single-cell multiomics database, current information available on the website will be beneficial for quick references, especially top peaks and the closest genes to these peaks.

**Reviewer #2**

Comments for the Author

In this manuscript by the Single Cell Atlas consortium, the authors present a database integrating single-cell multi-omics datasets across human adult and fetal healthy tissues. This appears to in principle represent the largest collection of processed and more readily accessible single-cell data, covering a large breath of human tissues. The authors use this database to showcase various integrative analyses of the diverse phenotypic variations across omics layers and tissues. Complementarily, the authors provide a web-based resource to facilitate the interactive visualization and exploration of the data. Overall, this is a valuable database aimed at facilitating the access of curated single-cell data to the research community.

However, this reviewer has concerns and suggestions about the curation of the data, applied quality control, the benefits and limitations of the database compared to existing ones, and the metadata annotation. The reviewer would also recommend enhancing the presented study cases, as they could be more meaningful. The value and relevance of comparing TCR sequences between donors are also unclear. Overall, the presented examples provide a rather limited showcase of the full potential and utility of the database.

**Response:**

We are grateful for your positive feedback and the valuable suggestions which have significantly contributed to the improvements made in our work. We acknowledge your concerns and suggestions regarding the curation of the data, applied quality control, the benefits and limitations of our database compared to existing ones, and the metadata annotation. In response to your feedback, we have carefully revised and expanded these aspects of our manuscript. We have placed particular emphasis on providing detailed meta and quality control information and made discussions of the strengths and limitations of our database in comparison to existing resources. Moreover, we have constructed relevant webpages to display metadata information and to provide data downloads, ensuring a more thorough understanding of the accompanying information and better benefit the users. Furthermore, we appreciate your point about the unclear value and relevance of comparing TCR sequences between donors. For this part, we aimed to examine at the macro-level, rather than the TCR sequence itself. We emphasized in the manuscript of stratifying the TCR expansion by groups (i.e., hyperexpansion, large expansion, etc.), as the cell type level information of such expansion characteristics may be beneficial to those who are interested in the expansion patterns across tissues in non-diseased samples. We will incorporate datasets of different cancer types in the future versions and such information will be even be more beneficial as direct expansion changes across cell types between normal and cancer can be readily made available across tissue types for users to retrieve and referred. Nonetheless, the current version still serves as a valuable, in-depth database for quick reference and inference down to many aspects within each omics and across omics.

We believe the modifications made in the revision will address your concerns and provide a more comprehensive demonstration of the database's capabilities. Once again, we sincerely appreciate your constructive feedback, as it has guided us in making substantial improvements to our manuscript. We are confident that the database now better showcases the full potential and utility of our work, allowing for more meaningful exploration and interpretation of the data. Thank you for your invaluable contribution to our work and we have listed down the responses to your comments by questions below.

Major Comments:

Extensive phenotypic explorations,

including gene expression, cell type visualizations, tissue-to-tissue, adult-to-fetal comparisons, transcription factor activities, cell-cell interactions, etc.

Data repository site for mass downloads

Basic gene expression and cell type visualizations. Analysis tools listed in their website were built by third parties and collectively listed as external links in the HCA websites, and they are from standalone solutions and are not endorsed by the HCA.

Basic gene expression and

metadata visualizations

1. The database integrates both adult and fetal human tissues utilizing multi-omic data and resources such as this one will be hugely beneficial for the single-cell community to navigate the ever-growing landscape of publicly available datasets. However, existing databases such as the single cell portal [(https://eur01.safelinks.protection.outlook.com/?url=https%3A%2F%2Fsinglecell.broadinstitut](https://eur01.safelinks.protection.outlook.com/?url=https%3A%2F%2Fsinglecell.broadinstitute.org%2Fsingle_cell&data=05%7C01%7Cxuexin.li%40ki.se%7Cd5a376022d244153af2108db3107e2d9%7Cbff7eef1cf4b4f32be3da1dda043c05d%7C0%7C0%7C638157682165608180%7CUnknown%7CTWFpbGZsb3d8eyJWIjoiMC4wLjAwMDAiLCJQIjoiV2luMzIiLCJBTiI6Ik1haWwiLCJXVCI6Mn0%3D%7C3000%7C%7C%7C&sdata=SsdwM5pMcmBfey1EuTeEvC0nEBMKYfxABaCP0%2FS5GlI%3D&reserved=0) [e.org%2Fsingle](https://eur01.safelinks.protection.outlook.com/?url=https%3A%2F%2Fsinglecell.broadinstitute.org%2Fsingle_cell&data=05%7C01%7Cxuexin.li%40ki.se%7Cd5a376022d244153af2108db3107e2d9%7Cbff7eef1cf4b4f32be3da1dda043c05d%7C0%7C0%7C638157682165608180%7CUnknown%7CTWFpbGZsb3d8eyJWIjoiMC4wLjAwMDAiLCJQIjoiV2luMzIiLCJBTiI6Ik1haWwiLCJXVCI6Mn0%3D%7C3000%7C%7C%7C&sdata=SsdwM5pMcmBfey1EuTeEvC0nEBMKYfxABaCP0%2FS5GlI%3D&reserved=0)_[cell&data=05%7C01%7Cxuexin.li%40ki.se%7Cd5a376022d244153af2108d](https://eur01.safelinks.protection.outlook.com/?url=https%3A%2F%2Fsinglecell.broadinstitute.org%2Fsingle_cell&data=05%7C01%7Cxuexin.li%40ki.se%7Cd5a376022d244153af2108db3107e2d9%7Cbff7eef1cf4b4f32be3da1dda043c05d%7C0%7C0%7C638157682165608180%7CUnknown%7CTWFpbGZsb3d8eyJWIjoiMC4wLjAwMDAiLCJQIjoiV2luMzIiLCJBTiI6Ik1haWwiLCJXVCI6Mn0%3D%7C3000%7C%7C%7C&sdata=SsdwM5pMcmBfey1EuTeEvC0nEBMKYfxABaCP0%2FS5GlI%3D&reserved=0) [b3107e2d9%7Cbff7eef1cf4b4f32be3da1dda043c05d%7C0%7C0%7C638157682165608180](https://eur01.safelinks.protection.outlook.com/?url=https%3A%2F%2Fsinglecell.broadinstitute.org%2Fsingle_cell&data=05%7C01%7Cxuexin.li%40ki.se%7Cd5a376022d244153af2108db3107e2d9%7Cbff7eef1cf4b4f32be3da1dda043c05d%7C0%7C0%7C638157682165608180%7CUnknown%7CTWFpbGZsb3d8eyJWIjoiMC4wLjAwMDAiLCJQIjoiV2luMzIiLCJBTiI6Ik1haWwiLCJXVCI6Mn0%3D%7C3000%7C%7C%7C&sdata=SsdwM5pMcmBfey1EuTeEvC0nEBMKYfxABaCP0%2FS5GlI%3D&reserved=0) [%7CUnknown%7CTWFpbGZsb3d8eyJWIjoiMC4wLjAwMDAiLCJQIjoiV2luMzIiLCJBTiI6Ik1h](https://eur01.safelinks.protection.outlook.com/?url=https%3A%2F%2Fsinglecell.broadinstitute.org%2Fsingle_cell&data=05%7C01%7Cxuexin.li%40ki.se%7Cd5a376022d244153af2108db3107e2d9%7Cbff7eef1cf4b4f32be3da1dda043c05d%7C0%7C0%7C638157682165608180%7CUnknown%7CTWFpbGZsb3d8eyJWIjoiMC4wLjAwMDAiLCJQIjoiV2luMzIiLCJBTiI6Ik1haWwiLCJXVCI6Mn0%3D%7C3000%7C%7C%7C&sdata=SsdwM5pMcmBfey1EuTeEvC0nEBMKYfxABaCP0%2FS5GlI%3D&reserved=0) [aWwiLCJXVCI6Mn0%3D%7C3000%7C%7C%7C&sdata=SsdwM5pMcmBfey1EuTeEvC0nE](https://eur01.safelinks.protection.outlook.com/?url=https%3A%2F%2Fsinglecell.broadinstitute.org%2Fsingle_cell&data=05%7C01%7Cxuexin.li%40ki.se%7Cd5a376022d244153af2108db3107e2d9%7Cbff7eef1cf4b4f32be3da1dda043c05d%7C0%7C0%7C638157682165608180%7CUnknown%7CTWFpbGZsb3d8eyJWIjoiMC4wLjAwMDAiLCJQIjoiV2luMzIiLCJBTiI6Ik1haWwiLCJXVCI6Mn0%3D%7C3000%7C%7C%7C&sdata=SsdwM5pMcmBfey1EuTeEvC0nEBMKYfxABaCP0%2FS5GlI%3D&reserved=0) [BMKYfxABaCP0%2FS5GlI%3D&reserved=0](https://eur01.safelinks.protection.outlook.com/?url=https%3A%2F%2Fsinglecell.broadinstitute.org%2Fsingle_cell&data=05%7C01%7Cxuexin.li%40ki.se%7Cd5a376022d244153af2108db3107e2d9%7Cbff7eef1cf4b4f32be3da1dda043c05d%7C0%7C0%7C638157682165608180%7CUnknown%7CTWFpbGZsb3d8eyJWIjoiMC4wLjAwMDAiLCJQIjoiV2luMzIiLCJBTiI6Ik1haWwiLCJXVCI6Mn0%3D%7C3000%7C%7C%7C&sdata=SsdwM5pMcmBfey1EuTeEvC0nEBMKYfxABaCP0%2FS5GlI%3D&reserved=0)),

the Human Cell Atlas

[(https://eur01.safelinks.protection.outlook.com/?url=https%3A%2F%2Fdata.humancellatlas.o](https://eur01.safelinks.protection.outlook.com/?url=https%3A%2F%2Fdata.humancellatlas.org%2F&data=05%7C01%7Cxuexin.li%40ki.se%7Cd5a376022d244153af2108db3107e2d9%7Cbff7eef1cf4b4f32be3da1dda043c05d%7C0%7C0%7C638157682165608180%7CUnknown%7CTWFpbGZsb3d8eyJWIjoiMC4wLjAwMDAiLCJQIjoiV2luMzIiLCJBTiI6Ik1haWwiLCJXVCI6Mn0%3D%7C3000%7C%7C%7C&sdata=7ksOX9Hlx%2FJ9osHweAGukrzIg9o%2FduFcdF2MiDLcJLQ%3D&reserved=0) [rg%2F&data=05%7C01%7Cxuexin.li%40ki.se%7Cd5a376022d244153af2108db3107e2d9%](https://eur01.safelinks.protection.outlook.com/?url=https%3A%2F%2Fdata.humancellatlas.org%2F&data=05%7C01%7Cxuexin.li%40ki.se%7Cd5a376022d244153af2108db3107e2d9%7Cbff7eef1cf4b4f32be3da1dda043c05d%7C0%7C0%7C638157682165608180%7CUnknown%7CTWFpbGZsb3d8eyJWIjoiMC4wLjAwMDAiLCJQIjoiV2luMzIiLCJBTiI6Ik1haWwiLCJXVCI6Mn0%3D%7C3000%7C%7C%7C&sdata=7ksOX9Hlx%2FJ9osHweAGukrzIg9o%2FduFcdF2MiDLcJLQ%3D&reserved=0) [7Cbff7eef1cf4b4f32be3da1dda043c05d%7C0%7C0%7C638157682165608180%7CUnknow](https://eur01.safelinks.protection.outlook.com/?url=https%3A%2F%2Fdata.humancellatlas.org%2F&data=05%7C01%7Cxuexin.li%40ki.se%7Cd5a376022d244153af2108db3107e2d9%7Cbff7eef1cf4b4f32be3da1dda043c05d%7C0%7C0%7C638157682165608180%7CUnknown%7CTWFpbGZsb3d8eyJWIjoiMC4wLjAwMDAiLCJQIjoiV2luMzIiLCJBTiI6Ik1haWwiLCJXVCI6Mn0%3D%7C3000%7C%7C%7C&sdata=7ksOX9Hlx%2FJ9osHweAGukrzIg9o%2FduFcdF2MiDLcJLQ%3D&reserved=0) [n%7CTWFpbGZsb3d8eyJWIjoiMC4wLjAwMDAiLCJQIjoiV2luMzIiLCJBTiI6Ik1haWwiLCJXV](https://eur01.safelinks.protection.outlook.com/?url=https%3A%2F%2Fdata.humancellatlas.org%2F&data=05%7C01%7Cxuexin.li%40ki.se%7Cd5a376022d244153af2108db3107e2d9%7Cbff7eef1cf4b4f32be3da1dda043c05d%7C0%7C0%7C638157682165608180%7CUnknown%7CTWFpbGZsb3d8eyJWIjoiMC4wLjAwMDAiLCJQIjoiV2luMzIiLCJBTiI6Ik1haWwiLCJXVCI6Mn0%3D%7C3000%7C%7C%7C&sdata=7ksOX9Hlx%2FJ9osHweAGukrzIg9o%2FduFcdF2MiDLcJLQ%3D&reserved=0) [CI6Mn0%3D%7C3000%7C%7C%7C&sdata=7ksOX9Hlx%2FJ9osHweAGukrzIg9o%2FduFc dF2MiDLcJLQ%3D&reserved=0)](https://eur01.safelinks.protection.outlook.com/?url=https%3A%2F%2Fdata.humancellatlas.org%2F&data=05%7C01%7Cxuexin.li%40ki.se%7Cd5a376022d244153af2108db3107e2d9%7Cbff7eef1cf4b4f32be3da1dda043c05d%7C0%7C0%7C638157682165608180%7CUnknown%7CTWFpbGZsb3d8eyJWIjoiMC4wLjAwMDAiLCJQIjoiV2luMzIiLCJBTiI6Ik1haWwiLCJXVCI6Mn0%3D%7C3000%7C%7C%7C&sdata=7ksOX9Hlx%2FJ9osHweAGukrzIg9o%2FduFcdF2MiDLcJLQ%3D&reserved=0),

or GTEX

[(https://eur01.safelinks.protection.outlook.com/?url=https%3A%2F%2Fgtexportal.org%2Fho](https://eur01.safelinks.protection.outlook.com/?url=https%3A%2F%2Fgtexportal.org%2Fhome%2FsingleCellOverviewPage&data=05%7C01%7Cxuexin.li%40ki.se%7Cd5a376022d244153af2108db3107e2d9%7Cbff7eef1cf4b4f32be3da1dda043c05d%7C0%7C0%7C638157682165608180%7CUnknown%7CTWFpbGZsb3d8eyJWIjoiMC4wLjAwMDAiLCJQIjoiV2luMzIiLCJBTiI6Ik1haWwiLCJXVCI6Mn0%3D%7C3000%7C%7C%7C&sdata=sKw7ooXLPGCK7Sc81Jy1gOZvn9KPVSFA6%2BNf%2F9Qhjmo%3D&reserved=0) [me%2FsingleCellOverviewPage&data=05%7C01%7Cxuexin.li%40ki.se%7Cd5a376022d244](https://eur01.safelinks.protection.outlook.com/?url=https%3A%2F%2Fgtexportal.org%2Fhome%2FsingleCellOverviewPage&data=05%7C01%7Cxuexin.li%40ki.se%7Cd5a376022d244153af2108db3107e2d9%7Cbff7eef1cf4b4f32be3da1dda043c05d%7C0%7C0%7C638157682165608180%7CUnknown%7CTWFpbGZsb3d8eyJWIjoiMC4wLjAwMDAiLCJQIjoiV2luMzIiLCJBTiI6Ik1haWwiLCJXVCI6Mn0%3D%7C3000%7C%7C%7C&sdata=sKw7ooXLPGCK7Sc81Jy1gOZvn9KPVSFA6%2BNf%2F9Qhjmo%3D&reserved=0) [153af2108db3107e2d9%7Cbff7eef1cf4b4f32be3da1dda043c05d%7C0%7C0%7C63815768](https://eur01.safelinks.protection.outlook.com/?url=https%3A%2F%2Fgtexportal.org%2Fhome%2FsingleCellOverviewPage&data=05%7C01%7Cxuexin.li%40ki.se%7Cd5a376022d244153af2108db3107e2d9%7Cbff7eef1cf4b4f32be3da1dda043c05d%7C0%7C0%7C638157682165608180%7CUnknown%7CTWFpbGZsb3d8eyJWIjoiMC4wLjAwMDAiLCJQIjoiV2luMzIiLCJBTiI6Ik1haWwiLCJXVCI6Mn0%3D%7C3000%7C%7C%7C&sdata=sKw7ooXLPGCK7Sc81Jy1gOZvn9KPVSFA6%2BNf%2F9Qhjmo%3D&reserved=0) [2165608180%7CUnknown%7CTWFpbGZsb3d8eyJWIjoiMC4wLjAwMDAiLCJQIjoiV2luMzIiL](https://eur01.safelinks.protection.outlook.com/?url=https%3A%2F%2Fgtexportal.org%2Fhome%2FsingleCellOverviewPage&data=05%7C01%7Cxuexin.li%40ki.se%7Cd5a376022d244153af2108db3107e2d9%7Cbff7eef1cf4b4f32be3da1dda043c05d%7C0%7C0%7C638157682165608180%7CUnknown%7CTWFpbGZsb3d8eyJWIjoiMC4wLjAwMDAiLCJQIjoiV2luMzIiLCJBTiI6Ik1haWwiLCJXVCI6Mn0%3D%7C3000%7C%7C%7C&sdata=sKw7ooXLPGCK7Sc81Jy1gOZvn9KPVSFA6%2BNf%2F9Qhjmo%3D&reserved=0) [CJBTiI6Ik1haWwiLCJXVCI6Mn0%3D%7C3000%7C%7C%7C&sdata=sKw7ooXLPGCK7Sc8](https://eur01.safelinks.protection.outlook.com/?url=https%3A%2F%2Fgtexportal.org%2Fhome%2FsingleCellOverviewPage&data=05%7C01%7Cxuexin.li%40ki.se%7Cd5a376022d244153af2108db3107e2d9%7Cbff7eef1cf4b4f32be3da1dda043c05d%7C0%7C0%7C638157682165608180%7CUnknown%7CTWFpbGZsb3d8eyJWIjoiMC4wLjAwMDAiLCJQIjoiV2luMzIiLCJBTiI6Ik1haWwiLCJXVCI6Mn0%3D%7C3000%7C%7C%7C&sdata=sKw7ooXLPGCK7Sc81Jy1gOZvn9KPVSFA6%2BNf%2F9Qhjmo%3D&reserved=0) [1Jy1gOZvn9KPVSFA6%2BNf%2F9Qhjmo%3D&reserved=0)](https://eur01.safelinks.protection.outlook.com/?url=https%3A%2F%2Fgtexportal.org%2Fhome%2FsingleCellOverviewPage&data=05%7C01%7Cxuexin.li%40ki.se%7Cd5a376022d244153af2108db3107e2d9%7Cbff7eef1cf4b4f32be3da1dda043c05d%7C0%7C0%7C638157682165608180%7CUnknown%7CTWFpbGZsb3d8eyJWIjoiMC4wLjAwMDAiLCJQIjoiV2luMzIiLCJBTiI6Ik1haWwiLCJXVCI6Mn0%3D%7C3000%7C%7C%7C&sdata=sKw7ooXLPGCK7Sc81Jy1gOZvn9KPVSFA6%2BNf%2F9Qhjmo%3D&reserved=0) provide similar functions and data modalities. Can the authors highlight distinguishing features, advantages, and limitations of their database more clearly (e.g., in form of a table) and do they foresee any integration of these multiple orthogonal efforts?

**Response:**

Thank you very much for your comments. We have compared the databases you have mentioned and compared with our database, and have listed the key features in **Table R1** as shown below:

**Strength/	Single Cell	Human Cell Atlas**

**Limitation	SingleCellAtlas	Portal	(HCA)	GTEx Portal**

Mainly for extensive phenotypic explorations across multiple omics and also served as a data

**Purpose**	repository site

Data repository site for mass downloads

Basic gene expression and cell type visualizations

Data repository site for mass downloads

**Data**

**Exploration features**

| **Single Cell Datasets** | Mostly are single-cell datasets, with bulk- sequencing datasets from the GTEx portal. | Mostly are single-cell datasets | Mostly are single-cell datasets | Mostly are bulk-sequencng datasets, only 8 tissues have single-cell data |
| --- | --- | --- | --- | --- |
| **Data Sources** | Publicly available datasets | Publicly available datasets | Multiple sources, including public and datasets produced by the HCA groups | Datasets produced by the GTEx consortium |
| **Data**  **Accessibility** | Publicly assessible data | Publicly  assessible  data | Publicly assessible data | Both publicly assessible data and protected sensitive data |
| **Available  Data Type** | Raw expression  matrices and  metadata | Raw expression matrices and metadata | Raw sequencing files, expression matrices and metadata | Raw sequencing files, expression matrices, metadata, clinical information |
| **Omics types** | Several omics, including scRNA- Seq, spatial transcriptomics, single-cell immune profiling, scATAC- Seq, mass cytometry (CyTOF), flow cytometry,and bulk RNA-Seq. | Most datasets included are single-cell RNA-sequencing (scRNA-Seq), with a small portion of spatial transcriptomic s and single- cell ATAC- Sequencing. Included datasets from the HCA. | Most datasets included are single-cell RNA- sequencing (scRNA- Seq), with a small portion of spatial transcriptomics and single-cell ATAC- Sequencing (scATAC- Seq). | Most datasets included are bulk RNA-Sequencing and genotyping datasets, with a small portion of scRNA-Seq datasets. |
| **Species** | Human datasets | Mostly human, but also included datasets from several other organisms. | Mostly human, but also included a small portion of mouse datasets. | Human datasets |

**Table R1.** Comparison of the SingleCellAtlas with three other databases.

In summary, the three databases (i.e., Single Cell portal, the HCA, and GTEx portal) served different purposes than ours. They are, in summary, database or repository sites for storing raw/processed study data for the benefit of the overall research community and do not contain in-depth analysis of the data present. They mainly served as platforms to archive these study data, and to provide download options and external links to the original datasets where they have downloaded and archived the same files on their database server. The datasets were kept separately and no integrative efforts were made. Moreover, they often only portray an overview of the cell type compositions or gene expressions in their archived study data, which limits more in-depth exploration of their data apart from the papers published. Additionally, more time and effort are needed for researchers who are interested in exploring their data to download and analyze in order to explore other in-depth aspects of the datasets. In other words, they are the upstream data sources of our SingleCellAtlas in terms of data retrieval and we extensively analyzed and presented the different phenotypic aspects of the datasets. SingleCellAtlas aims to serve as a multi-tissue healthy human single-cell multiomics atlas, for the research community to explore inter/intra-tissue specific heterogeneities within/between fetal and adult tissues across different molecular layers (or omics), as well as to data-mine the different aspects of various omics characteristics, such as transcription factor profiles of each cell type from fetal or adult tissues, differential gene expression signatures or ligand -receptor binding profiles, etc. Last but not least, single-cell experiments are still expensive at this stage, and many studies could only afford the sequencing of a few samples for their studies. As such, mass integration and in-depth assessment of these data sources not only increase study effect size, but also allow comparisons of large tissue samples that could allow us to draw conclusions that better represent the actual study population concerned. As such, the three databases are currently not able to offer such comparisons at the multi-omics levels.

2. The number of cells included in the analysis is inconsistent/confusing in both the paper and the website. Ultimately, only the cells meeting the respective quality control (QC) criteria will likely be used by the research community and should therefore be the ones that are primarily highlighted.

**Response:**

Thank you very much for your suggestions. We have gone through every single omics file across the tissues and made sure that the number of cells or samples, especially in **Supplementary Table S1**, is correct, and the current table is the most up-to-date numbers for the current database version SCA V1.0.0.

3. Related to the previous point, the authors should provide QC plots/information for all relevant data for the reader to get a sense of how the applied thresholds (e.g., maximum 30,000 number of peaks for scATAC-seq) or exclusion of thresholds (e.g., TSS enrichment was not used?) affect filtering. This may in fact also vary from tissue to tissue depending on the experimental tissue processing and resulting quality. Also, the filtering of cells with a mitochondrial content > 5% in the single-cell RNA-seq datasets might be too strict for many human tissues with higher physiologic mitochondrial content (e.g., heart) and this threshold probably makes a significant difference depending on whether nuclei or cells were used for the transcriptomic profiling. As such, QC metrics might need to be adjusted depending on the tissue of origin and experimental setup. In general, we are concerned about the very high proportion of cells discarded after applying used thresholds (Introduction: We profiled 66,863,102 cells from scRNA-Seq, Results: After filtering, 2,866,972 high-quality cells were obtained for scRNA-Seq). As less than 5% of cells remain after filtering, this appears very unusual compared to what is typically reported in the literature.

**Response:**

Thank you so much for the suggestions. For the single-cell ATAC-Sequencing (scATAC-Seq) datasets, unfortunately, many papers did not provide a full dataset, such as lack of fragment data. For instance, a typical 10X scATAC-Seq data Cell Ranger output consists of input files such as the fragment or peak file as shown in the following link: [https://www.10xgenomics.com/resources/datasets/10k-human-pbmcs-atac-v2-chromium-](https://www.10xgenomics.com/resources/datasets/10k-human-pbmcs-atac-v2-chromium-controller-2-standard)

[controller-2-standard,](https://www.10xgenomics.com/resources/datasets/10k-human-pbmcs-atac-v2-chromium-controller-2-standard) yet many failed to provide such information, especially for fragment files, and most of them only contained the ‘peak by cell matrix’ files. This is restricting our analysis for scATAC-Seq to a great extent, such as TSS enrichment cannot be performed due to the lack of fragment data. To avoid any bias quality control, at the general basis, we filtered for the information available for all the scATAC-Seq datasets, which is the peak number, and based on the threshold as indicated in the methods section. Luckily for other omics, most of the input data

are fully available and did not become a constraint for us during the analysis. Therefore, for the scRNA-Seq, we provided the QC plots for the batch control step, and the plots are available under the Source tab, after the first table on the page [(www.singlecellatlas.org/sources](http://www.singlecellatlas.org/sources)). We will continue to populate the QC visualization parts on this page with other steps and omics outputs, as the reproducibility time is huge. We will furnish this part and incorporate the QC for other omics in the next database version.

As for the mitochondrial content threshold, we have also considered if we need to have a adjusted threshold cutoff. Accordingly, as our database will be getting larger and larger in the future versions, we wish to include as less noise in the data as possible. To choose between higher threshold with cells with higher qualities but losing a high number of cells, or to incorporate a higher threshold to include more cells (i.e. more cell types too) while long termwise may added more noise to the huge dataset, especially when we control the batches by project. To strike a balance, we include a soft 5% cut-off approach, i.e., if the tissue (not the sample) has loose almost all cells with little remaining for the downstream analysis, we will increase the cut-off to 30%. Please let us know if you have a better approach than this and we will implement in the next version of our database. Nonetheless, we fully agreed with you for such concerns, yet other problems as mentioned may arise too, and it is difficult to strike a balance.

As for the high number of cells been removed after QC, to provide an example, we used the samples from Gene Expression Omnibus (GEO) accession GSE134355 obtained from the Human Cell Landscape [4] and filtered the cells at 5% percentage of mitochondria genes expressed. As seen from **Figure R2**, most cells were damaged cells (>=5% cutoff). These substantially reduced the number of cells after the QC procedure.

**Figure R2.** The percentage of damaged cells, marked by the percentage of mitochondria genes expressed, in the Human Cell Landscape [4] dataset.

4. The paper is, unfortunately, lacking metadata. There is no information about the number of donors contributing to the different datasets, while it highly preconditions the representativity of the results and will certainly be a legitimate concern for the users. This information is particularly crucial while comparing the T cells/B cells clonotypes. For each single-cell modality and tissue, the authors should provide a UMAP where the different individuals are projected and a plot summarizing each individual's relative contribution to the pool of cells. They should also include a general plot indicating the number of individuals contributing to each tissue for each modality and provide a supplementary table with basic donor data (e.g., age, sex), or at the very least, the link to the metadata page on their website.

**Response:**

Thank you very much for the useful comment. For each omics and tissue, we have provided a general plot indicating the sample number in each tissue and modality for both the adult and fetal groups. The plot is now in **Supplementary Figure S1** and subsequent figures in the supplementary were moved to

the next supplementary figure indices in sequence. As for the basic donor data, we have provided a supplementary table (**Supplementary Table S8**) to include the title of the project, original accession number, as well as web link to the data source for each project. This information is also interactively available on the website under the Source tab: [www.singlecellatlas.org/sources.](http://www.singlecellatlas.org/sources) For the age and sex data, may are still missing due to the inconsistency of the information made available to the public and varied in querying difficulties, i.e., some are present in the original data repository site with a meta file containing such information which is easy to retrieve, some are present in individual sample page which sometimes the names of their sample accession number (e.g., GSM number in the GEO repository) may not be the sample id found in the actual data, and thus increased the difficulties. For some others, such information may be found in the supplementary table of their published work, while some were hidden in the figures or among the main text, making the retrieval process even more difficult. Nonetheless, as you may remember, we made efforts in incorporating age and sex information in the Source page. For the current SCA V1.0.0 database version, we decided to remove the sex and age information and will incorporate that in for the next version.

5. The authors make an indistinctive use of t-SNE and UMAP 2D/3D visualizations. For the sake of consistency and because this is an integrative resource, this reviewer recommends using one of the two methods for all the figures.

**Response:**

Thank you for the great suggestion and we agreed that the consistency should be followed. We have now unified the visualization to confine the figures to UMAP visualizations for both 2D and 3D visualizations rather than to use a mixture of visualizations in the figures.

6. The authors implement the Harmony algorithm to correct inter-dataset batch effects. However, they do not take into account the potential batch effects existing within each data source. The authors should therefore provide a UMAP colored by data source in order to assess the potential intra-dataset batch effects.

**Response:**

Thank you very much for the useful suggestions. We have now provided PCA (before integration), Harmony, UMAP, and tSNE (after integration) visualizations colored by data source on our website, and can be interactively accessed under the Sources page [(www.singlecellatlas.org/sources](http://www.singlecellatlas.org/sources)).

7. Multiple testing corrections were processed using both Bonferroni and Benjamini-Hochberg methods without further clarification on why these two methods were used indistinctly. As for comment 5, the authors should be consistent in their methodology throughout the manuscript.

**Response:**

Thank you for the great suggestion. We are more prone to use the Benjamini-Hochberg (BH) approach to control for the false discovery rate in many of our analyses steps as shown in the methods section in the manuscript, as it offers a more balanced approach by allowing for a controlled number of false positives while maintaining statistical power. Yet as you noticed, we also deployed the Bonferroni correction, especially in the differential expression/peak analyses, as this is not only the default method implemented by the Seurat package [1], but also it is a more stringent form of error control than the BH approach to conservatively minimize false positives, i.e., the significant differentially expressed genes in our results are more strictly controlled for false positives. Many have also raised the question to change the Seurat package to allow for BH approach [(https://github.com/satijalab/seurat/issues/191](https://github.com/satijalab/seurat/issues/191)) but the developer has declared that they will not implement it in. While we fully understand your concern on harmonizing the choice of the method used, the amount of effort in updating all the results across omics and tissues on the website and in the manuscript maybe enormous. However, for the coming versions, we will make efforts in switching the results timely from Bonferroni to the BH approach and will state which sections has been changed in bullet points during version control on the website. Despite so, maintaining a strict control over false positives is crucial in differential expression analysis, and that the Bonferroni method may be preferred. However, there is always a balanced trade-off between controlling false positives and maintaining statistical power, especially in exploratory analysis, and that we felt that the Benjamini-Hochberg method is often more suitable.

8. The authors should provide use cases demonstrating the usefulness of the database. Can users integrate their own data with the provided multimodal data? Is there any workflow they could implement to do so?

**Response:**

Thank you very much for your suggestions. We have been thinking of that too and have created workflows across the omics types we have and have constructed a web platform with comprehensive workflow for omics analyses, so that the reference datasets in the SingleCellAtlas can be used for analysis by the researchers. This is another project of our team and is still under development. We believe that the strong analytic support together with the current data resources and phenotypic features across omics, will make the database even more powerful in the future.

9. Single-cell research is a rapidly evolving field, continually including new methods to generate or analyze data. The authors did not clarify how the database will be maintained and updated. Is it planned to integrate other omics modalities, analyses, and datasets in the future?

**Response:**

Thank you very much for the thoughtful comment. We have provided versioning of the database and will update periodically to fill in the gaps of the tissues for each omics types, while at the same time, to expand the sample size in each tissue. On a timely basis, we will made improvements to the database and will indicate the new features or new datasets added in point forms, while updating the accession number accordingly. Currently, we versioned the database as SCA V1.0.0. Specifically for new datasets, we will summarize on a yearly basis at the end of each year, the new data sets published in the same year, and include them periodically together with new phenotypic features. As for integration and analysis, indeed, we have another project to provide substantial analytic computational frameworks for analyzing different omics datasets. The aim of our atlas is not only to provide useful data and phenotypic features resources, but also to allow easy and fast accessibility to data resources across various omics and provide a platform for cross comparison of their datasets with our dataset. Currently the analytic frameworks are still under development, and once it is published, we will link up the project up on the SingleCellAtlas website.

0. After all, flow cytometry and CyTOF datasets were of limited value. While the authors should discuss the consideration of such data, their advertising in the introduction and in the results section is not warranted, as also no such data is accessible on the website right now.

**Response:**

Thank you very much for your thoughtful considerations. We agreed with you that flow and CyTOF datasets were of limited value, yet given the number of tissues we have collected for the two omics, the cell types found across the tissues in these omics can serve as references to the cell types that can be detected based on a certain reference panels, and their relative proportions with respect to each tissue type. We believe that such information can be valuable to researchers to serve as quick references to panel design and cell type presence based on the selected markers. Moreover, researchers who are interested in comparing across multiple omics for the tissue of interest may also be of value to them. Moreover, the data downloaded can also be used for multi-omics integration procedure between single-cell RNA-Sequencing (scRNA-Seq) data and flow cytometry or CyTOF datasets, based on the overlapping markers. As for the datasets, they are accessible on the website at [www.singlecellatlas.org/download.](http://www.singlecellatlas.org/download) Currently, the download page is password-protected (password to access the page: *gbreviewers*) and will be made publicly accessible once published. By clicking on the tissues/omics/age groups/etc. of interest on the page, download links to all relevant files will be compiled in a text readable .txt file and can be downloaded in a batch.

11. Overall, the level of granularity of cell type/state annotation (330 cell types) appears rather limited. Similar and comparatively less comprehensive efforts already identify up to 657 subtypes DOI: 10.1126/science.aba7721. Now, this reviewer understands the immense complexity of harmonizing cell type and state annotation, which should also be thoroughly discussed by the authors. The current categorization of the annotated 330 cell types into 17 major cell type classes and comparisons thereof is a vast oversimplification of the actual biological complexity, whereas resolving this complexity, should be the major emphasis of these and other single-cell databases.

**Response:**

Thank you very much for the thoughtful comments. We agreed with you that the level of granularity of the annotation is limited. Our database is a comprehensive database integrating various datasets with varying quality control (QC) performed by the studies themselves. Therefore, we did not use the original thresholds indicated by the studies themselves. Instead, we performed data cleaning with a universal cut-off to remove damaged and low-quality cells with extensive mitochondrial leakage. This was done homogenously across all datasets for the same omics (here scRNA-Seq datasets) and we lost cell types along the QC process. Moreover, during the integration process, the remaining cells with much similar expression profiles will be clustered together, and due to the amount of cells lost during QC, the remaining cells clustered together reduced the level of granularity to a greater extent. As such, we considered to lower the level of granularity to ensure that our database is a relatively major and reliable reference accordingly rather than to focus in identifying rare cell types and increase the granularity. As the database expands in the future, rare cell types passing the QC procedure will be retained, and these cells will reflect a better quality of phenotypic discoveries than to increase annotation granularity while having less cells.

Minor Comments

1. Pseudotime is a complex concept that, despite its name, should not be confused with real-

time. Therefore, the authors should not mention terms such as "earlier" transitions.

**Response:**

Thank you very much for the careful inspection and the great advice. We totally agreed with you. We understand that pseudotime infers the temporal ordering of the cells and cannot be assumed to equate it to the actual biological developing time. We have now avoided such term and fixed the line on “earlier” transitions on page 8, second paragraph.

**References**

1. Hao Y, Hao S, Andersen-Nissen E, Mauck WM, 3rd, Zheng S, Butler A, Lee MJ, Wilk AJ, Darby C, Zager M, et al: **Integrated analysis of multimodal single-cell data.** *Cell* 2021, **184:**3573-3587.e3529.
2. Aibar S, González-Blas CB, Moerman T, Huynh-Thu VA, Imrichova H, Hulselmans G, Rambow F, Marine J-C, Geurts P, Aerts J, et al: **SCENIC: single-cell regulatory network inference and clustering.** *Nature Methods* 2017, **14:**1083-1086.
3. Stuart T, Srivastava A, Madad S, Lareau CA, Satija R: **Single-cell chromatin state analysis with Signac.** *Nature Methods* 2021, **18:**1333-1341.
4. Han X, Zhou Z, Fei L, Sun H, Wang R, Chen Y, Chen H, Wang J, Tang H, Ge W, et al: **Construction of a human cell landscape at single-cell level.** *Nature* 2020, **581:**303­309.

**Second round of review**

**Reviewer 1**

Authors addressed all my comments and I suggest to accept this manuscript.

**Reviewer 2**

In their revised version, the authors have made an important effort to increase the consistency of their database by unifying latent space visualization, batch control QC plots, and including further metadata information. The plan to include new datasets on a yearly basis is also reasonable and should be briefly mentioned in the main text of the manuscript.

Furthermore, the authors have made a great effort to gather a considerably important number of studies and facilitate their access to other researchers. For this reason, this reviewer believes the SingleCellAtlas project has great potential. However, the resource remains considerably handicapped given the use of stringent quality control metrics. This not only affects the important data collection effort they have made but also raises issues regarding the absence or minimally under- if not misrepresentation of numerous cell types, which should remain an absolute focus when building an exhaustive atlas. In this regard, carefully evaluated inclusion criteria are required (as opposed to stringent and homogenous exclusion criteria).

As explained in the following comments (directly following up on the comments/responses to the first review), the reviewer is concerned about the absence of crucial information that could impair the usability of this resource. This includes the absence of fragment files in some of the included scATAC-seq experiments and more importantly, the absence of basic QC plots that are essential for guiding further analyses. Additionally, the authors included donors with very little contribution in terms of cell number, which can artificially inflate the impression of capturing inter-individual heterogeneity.

In summary, the authors improved the manuscript by providing metadata and by uniformizing methods. Yet, major comments were not properly addressed, due to which this reviewer has significant concerns about the publication of the manuscript in its current form.

1. The reviewer believes a more exhaustive characterization of the existing resources is needed to display the advantages and disadvantages of the SingleCellAtlas. The authors did not include any other database, but the ones already mentioned by this reviewer, nor did they clarify whether a more exhaustive search was done. In addition, the descriptions used to compare the four selected resources are rather subjective. For instance, the authors describe the Single Cell Portal as a "Data repository site for mass downloads", ignoring the exploratory modules it includes. Most of the descriptions include wordings such as "mostly", "mainly" or "several". This reviewer suggests the use of objective terminology, detailing specific numbers and comparison metrics. Furthermore, the authors could provide a graphic representation (e.g., histograms) comparing the number of datasets/cells/represented tissues/data modalities between databases. The major novelty of SingleCellAtlas is the integration of multi-omics data. However, as shown in Supplementary Figure 1, multimodal datasets only represent a small proportion of the whole database and surprisingly do not include multi-ome (scATACseq + scRNAseq) or CITE-seq data.

2. The main text (Introduction) still mentions the total number of cells that were initially profiled rather than the number of cells remaining after quality control. Likewise, the text attached to the UMAP representing all the scRNA-seq data (accessible via the website, see screenshot), for example, still mentions all included cells as opposed to those remaining following QC (around 2 million cells).

Although the authors have provided updated post-qc numbers in the Results section, this reviewer can only reemphasize, given the unusually high number of cells discarded in this work, that the post-qc numbers, and not the number of screened cells, are being properly displayed in the full manuscript/website. This is essentially the common scientific practice in the field.


3. This reviewer understands the difficulties of accessing and obtaining publicly available data. However, as the aim of the SingleCellAtlas is to facilitate the access of single-cell data to the research community, the reviewer believes the absence of some specific files will restrict the database's usability. For instance, in the case of scATAC-seq experiments, fragment files are fundamental for data integration, and therefore, lacking this information will largely limit its use.

Regarding the mitochondrial content, the reviewer thinks that the default threshold of 5% remains too stringent. In fact, it has been previously shown that 10% is more appropriate for human normal tissue (https://doi.org/10.1093/bioinformatics/btaa751). In addition, according to figure R2, some samples contain very few cells after filtering with a threshold of 5%, thus contributing very little to the whole dataset. The authors mention also losing cell types (Response to major comment 11), which is very problematic in the context of building an exhaustive atlas. This could also be improved with a higher mitochondrial percentage threshold. Increasing this default value for some tissues as the authors proposed, is also very important but should rather be guided by biological reasons. Moreover, it was not clear to this reviewer whether this will be implemented in a future version or in the current one.

Overall, this reviewer still has concerns about the inclusion of samples with very few good-quality cells or lacking data. Finally, this reviewer reemphasizes the inclusion of pre-filtering QC plots for a better understanding of the current data quality metrics.

4.-6. The reviewer is satisfied with these responses.

7. The reviewer understands the authors used the default parameters for the distinct methodologies applied. Still, implementing a BH correction in the p-value list generated by the FindMarkers function shouldn't be a limitation. This reviewer hopes this point will be addressed in future releases.

8. This reviewer believes this question was not properly addressed. While it is understandable the authors might have future projects under development, it would still be necessary to provide "use cases demonstrating the usefulness of the database" in this first release. Furthermore, the authors do not clarify whether users can integrate their own data with the provided multimodal data.

9. The authors should include this clarification in their main text.

10. The reviewer is satisfied with this response.

11. The authors clarify that the use of a universal cut-off to remove low-quality and damaged cells has been key in the loss of data granularity. Indeed, after looking at some tissues manually, this review can only deplore the absence of major cell types (e.g., hepatic stellate cells in the liver). For this reason, as stated in Major comment 3, this reviewer reemphasizes that the authors must reconsider their QC thresholds (if necessary, in a cell-type specific manner) and adjust their analysis workflow to the current best practices. In the end, excluding close to 63 out of 66 million cells for the scRNA-seq data, severely limits the scope of the SingleCellAtlas the authors aim to provide for the community.

Minor comments:
1. The reviewer is satisfied with this response.

**Authors Response**

**Point-by-point responses to the reviewers’ comments:**

**Reviewer #1**

Authors addressed all my comments and I suggest to accept this manuscript.

**Response:**

We are deeply appreciative of the substantial effort expended in reviewing our manuscript and web-based platform. We recognize the intensity of the review process and value the time dedicated to our work. We will continue to work on our database and update it periodically, by adding new data and features to maintain its relevance and enhance its value to a broader audience.

**Reviewer #2**

In their revised version, the authors have made an important effort to increase the consistency of their database by unifying latent space visualization, batch control QC plots, and including further metadata information. The plan to include new datasets on a yearly basis is also reasonable and should be briefly mentioned in the main text of the manuscript.

Furthermore, the authors have made a great effort to gather a considerably important number of studies and facilitate their access to other researchers. For this reason, this reviewer believes the SingleCellAtlas project has great potential. However, the resource remains considerably handicapped given the use of stringent quality control metrics. This not only affects the important data collection effort they have made but also raises issues regarding the absence or minimally under- if not misrepresentation of numerous cell types, which should remain an absolute focus when building an exhaustive atlas. In this regard, carefully evaluated inclusion criteria are required (as opposed to stringent and homogenous exclusion criteria).

As explained in the following comments (directly following up on the comments/responses to the first review), the reviewer is concerned about the absence of crucial information that could impair the usability of this resource. This includes the absence of fragment files in some of the included scATAC-seq experiments and more importantly, the absence of basic QC plots that are essential for guiding further analyses. Additionally, the authors included donors with very little contribution in terms of cell number, which can artificially inflate the impression of capturing inter-individual heterogeneity.

In summary, the authors improved the manuscript by providing metadata and by uniformizing methods. Yet, major comments were not properly addressed, due to which this reviewer has significant concerns about the publication of the manuscript in its current form.

**Response:**

First of all, we would like to thank you for the extensive effort in reviewing our manuscript, especially at such an intensive level of information from us. We are grateful for your insightful feedback and your recognition of the potential of SCA. Your comments have given us essential guidance on how to improve the quality, consistency, and usability of our database. We are particularly appreciative of the time and expertise you have invested in our work.

We have now included a statement in the main text that we will update and add new datasets on a yearly basis at the end of the Discussion section of the main text in the manuscript. With regards to the stringent quality control metrics, we really appreciate the thoughtful concern raised by you, and have now adopted the threshold suggested by you and update the database based on the new threshold.

We acknowledge the concerns you raised about the stringent quality control metrics. It is not our intention to exclude or misrepresent any cell types in the SingleCellAtlas. Our concern was that, if we continue to update and include new datasets, the number of cells with comparatively less viable quality will expand exponentially too. To compromise, we decided to take stringency and higher quality over quantity. However, we are also aware of concerns raised by you with regards to the stringent cutoff. To address your concern, we have now re-evaluated the inclusion criteria and adjusted our quality control thresholds based on your suggested mitochondrial content threshold (< 10% now) to ensure a higher cell type coverage and thus more accurate representation. We concur that it is of paramount importance to ensure the exhaustiveness of the atlas.

Your feedback regarding the missing fragment files in the scATAC-seq experiments is duly noted. We have now remedied this by uploading and incorporating the missing files into the database. We have extensively investigated all published datasets provided by these scATAC-seq studies, and for some tissues, the files are indeed incomplete, or the files provided are less suitable for displaying on a tissue basis. There is no problem with getting complete datasets with regards to other omics types, but for scATAC-seq, many users seemed to upload partial input files, such that, some of the input files are missing from the provider end, making the analysis procedure more difficult. Nonetheless, we have incorporated as many features as we could based on the information provided for scATAC-seq data, and will continue to update the database when new studies are released, and we believe that the data will be more and more vibrant and complete by adding new studies.

For the basic QC plots, visualizations can be found in the bottom of the page, after the section of the data source visualization on [https://www.singlecellatlas.org/sources.](https://In) In response to your concerns about the inclusion of donors with minor cell number contributions, we have revised our methods to prevent the artificial inflation of inter-individual heterogeneity. We now take into account the contribution of cell numbers from each donor to ensure more balanced representation.

We believe that with these changes, we have substantially improved the manuscript and addressed your primary concerns. We hope that our revisions now meet the concerns raised by you and look forward to any further comments or suggestions you may have. Specifically, we have provided point-to-point response to the comments below.

1. The reviewer believes a more exhaustive characterization of the existing resources is needed to display the advantages and disadvantages of the SingleCellAtlas. The authors did not include any other database, but the ones already mentioned by this reviewer, nor did they clarify whether a more exhaustive search was done. In addition, the descriptions used to compare the four selected resources are rather subjective. For instance, the authors describe the Single Cell Portal as a “Data repository site for mass downloads”, ignoring the exploratory modules it includes. Most of the descriptions include wordings such as “mostly”, “mainly” or “several”. This reviewer suggests the use of objective terminology, detailing specific numbers and comparison metrics. Furthermore, the authors could provide a graphic representation (e.g., histograms) comparing the number of datasets/cells/represented tissues/data modalities between databases. The major novelty of SingleCellAtlas is the integration of multi-omics data. However, as shown in Supplementary Figure 1, multimodal datasets only represent a small proportion of the whole database and surprisingly do not include multi-ome (scATACseq + scRNAseq) or CITE-seq data.

**Response:**

We deeply appreciate your insightful feedback and constructive recommendations. We recognize the significance of presenting SingleCellAtlas in a clear and objective manner that distinguishes it from existing databases. While many current databases specialize in single omics, primarily scRNA-seq, we initially did not consider these given they do not fall within the ambit of single-cell multiomics databases. However, we have now expanded our previous comparison table to include additional databases and more precise comparison metrics, and the table can be found in supplementary Table S1.

The key innovation of SingleCellAtlas is not simply the integration of multi-omics data, but its role as a comprehensive repository containing phenotypic attributes of over 100 adult and fetal tissues across eight different omics fields. In this sense, the phenotypic features of each omics are distinctly displayed within SingleCellAtlas. No other database has offered such a broad perspective on multiple omics multi-perspective phenotyping. We acknowledge your concern about the relatively small proportion of multimodal datasets. We are actively sourcing new datasets to enhance our coverage in this area, specifically aiming to incorporate scATACseq + scRNAseq and CITE-seq data in our database. As mentioned, obtaining complete datasets (emphasizing the term complete, as while datasets exist, their input files often are incomplete) for scATAC-seq from published resources is challenging, hence limiting our ability to offer a comprehensive multimodal visualization of scATAC-seq datasets, let alone integrate scATAC-seq with scRNA-seq data across various tissues. Nonetheless, we are committed to incorporating these modalities whenever new datasets become available in future iterations of SingleCellAtlas.

2. The main text (Introduction) still mentions the total number of cells that were initially profiled rather than the number of cells remaining after quality control. Likewise, the text attached to the UMAP representing all the scRNA-seq data (accessible via the website, see screenshot), for example, still mentions all included cells as opposed to those remaining following QC (around 2 million cells).

Although the authors have provided updated post-qc numbers in the Results section, this reviewer can only reemphasize, given the unusually high number of cells discarded in this work, that the post-qc numbers, and not the number of screened cells, are being properly displayed in the full manuscript/website. This is essentially the common scientific practice in the field.

**Response:**

We appreciate your astute feedback and the time you have dedicated to this review. In light of your comments, we have updated the introduction in the main text to accurately mentioned that the numbers mentioned are before quality control. As quality control steps are needed to be mentioned before showing the actual cell counts, the post-quality control numbers are more appropriate to be displayed under the Results section after mentioning quality control steps. Therefore, to ensure better clarity, we have used the phrasing, “prior to quality control filtering, we have collected..” to specify that the numbers we presented in the Introduction are the raw collected numbers, and we have also highlighted in the Results section of the manuscript, the post-filtering counts for each omics type. We acknowledge the crucial importance of accurately reporting the final number of cells remaining post-quality control. In hindsight, we concur that referring to the total number of initially profiled cells without an explicit emphasis on the post-quality control numbers could lead to potential misunderstandings. Your recommendation aligns with the standard scientific practice in our field, and we have amended our manuscript and website to better reflect this important detail.

3. This reviewer understands the difficulties of accessing and obtaining publicly available data. However, as the aim of the SingleCellAtlas is to facilitate the access of single-cell data to the research community, the reviewer believes the absence of some specific files will restrict the database's usability. For instance, in the case of scATAC-seq experiments, fragment files are fundamental for data integration, and therefore, lacking this information will largely limit its use.

Regarding the mitochondrial content, the reviewer thinks that the default threshold of 5% remains too stringent. In fact, it has been previously shown that 10% is more appropriate for human normal tissue (<https://doi.org/10.1093/bioinformatics/btaa751)>. In addition, according to figure R2, some samples contain very few cells after filtering with a threshold of 5%, thus contributing very little to the whole dataset. The authors mention also losing cell types (Response to major comment 11), which is very problematic in the context of building an exhaustive atlas. This could also be improved with a higher mitochondrial percentage threshold. Increasing this default value for some tissues as the authors proposed, is also very important but should rather be guided by biological reasons. Moreover, it was not clear to this reviewer whether this will be implemented in a future version or in the current one.

Overall, this reviewer still has concerns about the inclusion of samples with very few good-quality cells or lacking data. Finally, this reviewer reemphasizes the inclusion of pre-filtering QC plots for a better understanding of the current data quality metrics.

**Response:**

Thank you so much for your concerns and suggestions. For the scATAC-seq data, we have taken action to enhance the scATAC-seq database by thoroughly examined the published datasets provided by these scATAC-seq studies. For certain tissues, we found that the files were indeed incomplete or less suited for in-depth analysis (i.e., only fragment files are provided yet peak counts across cells are not included, e.g., [https://www.ncbi.nlm.nih.gov/geo/query/acc.cgi?acc=GSE184462)](https://www.ncbi.nlm.nih.gov/geo/query/acc.cgi?acc=GSE184462). While obtaining complete datasets for other omics types is not an issue, we found that for scATAC-seq, many published studies appear to have uploaded only partial input files (either fragment files or peak counts are missing). This means that some input files were missing from the end of the providers, limiting in-depth analysis procedure. Nevertheless, we have processed and phenotyped as much as possible, including top coaccessible peak regions in each cell type across tissues based on the provided scATAC-seq data and we will continue to update the database as new studies are published. We are confident that with each new addition, the scATAC-seq database will grow more comprehensively and robustly.

We recognize and appreciate the critical points you have raised about our stringent quality control metrics. Our intention with SingleCellAtlas was never to omit or misrepresent any cell types. Our concern was that, as we perpetually add and update datasets, the quantity of cells of relatively lower quality would correspondingly increase. Our initial choice was to prioritize quality over quantity by maintaining stringent criteria. However, in light of the concerns you have expressed about these rigid cut-offs, we have revisited and adjusted our quality control thresholds, such as the mitochondrial content threshold now being set to <10% as suggested by you. This adjustment will allow a broader coverage of cell types for a more faithful representation, thereby ensuring the comprehensiveness of the atlas, which we agree is of paramount significance.

4.-6. The reviewer is satisfied with these responses.

**Response:**

Thank you for the positive comment.

7. The reviewer understands the authors used the default parameters for the distinct methodologies applied. Still, implementing a BH correction in the p-value list generated by the FindMarkers function shouldn’t be a limitation. This reviewer hopes this point will be addressed in future releases.

**Response:**

Thank you for your suggestions. We have now included a BH correction by the FindMarkers function (refer to supplementary Table S3), and we will stick to BH correction (a single form of correction method) in any further release.

8. This reviewer believes this question was not properly addressed. While it is understandable the authors might have future projects under development, it would still be necessary to provide “use cases demonstrating the usefulness of the database” in this first release. Furthermore, the authors do not clarify whether users can integrate their own data with the provided multimodal data.

**Response:**

We appreciate your feedback and understand the need for more illustrative examples on how to leverage the SingleCellAtlas (SCA) for comprehensive multiomics analysis. As exemplified in Figures 5 and 6 of our manuscript, we've conducted an in-depth multiomics study of colon tissues using all available omics data from SCA. We believe that these figures, along with the associated analyses detailed in the main text, can serve as valuable blueprints for users on how to exploit the resources offered by SCA.

Thank you for your feedback. As shown in Figure 5 and 6 of the manuscript, we have indeed used colon as an example, by utilizing all available omics data provided by SCA, and conducted a comprehensive multiomics analysis of colon tissues. We hope that the examples and phenotypic analysis provided by Figure 5 and 6, as well as interpreted analysis results shown in the main text, would provide to the users with ideas on how to make use of the database provided by SCA and conduct a thorough multiomics analysis. Additionally, we provide a walkthrough of use cases of the platform under the tab "Demonstration". As the datasets are made downloadable under the "Download" tab on the SCA website, users could freely analysis the provided datasets with or without integrating their own data locally, depending on their research of interest. As mentioned, as the analysis part is part of the other project, nonetheless, similar to the Analysis Tools page on the Human Cell Atlas [(https://data.humancellatlas.org/analyze)](https://data.humancellatlas.org/analyze) where they included a list of external tools (please note that the tools they have provided are built by third parties, as mentioned on the Analysis Tools page itself), we have also dedicated a page under the tab “Analyze”, displaying a list of tools [(https://singlecellatlas.org/analyze)](https://singlecellatlas.org/analyze), either online analysis tools (for small datasets), or offline tools (for large datasets) for users to carry out integrative analysis. Details and examples can be found on the page of each individual tools.

9. The authors should include this clarification in their main text.

**Response:**

Thank you so much for your concerns. We have now added a statement at the end of the Discussion section of the main text in the manuscript to make readers aware that we will update SCA on a timely basis. Many thanks for your suggestions.

0. The reviewer is satisfied with this response.

**Response:**

Thank you so much for your feedback.

11. The authors clarify that the use of a universal cut-off to remove low-quality and damaged cells has been key in the loss of data granularity. Indeed, after looking at some tissues manually, this review can only deplore the absence of major cell types (e.g., hepatic stellate cells in the liver). For this reason, as stated in Major comment 3, this reviewer reemphasizes that the authors must reconsider their QC thresholds (if necessary, in a cell-type specific manner) and adjust their analysis workflow to the current best practices. In the end, excluding close to 63 out of 66 million cells for the scRNA-seq data, severely limits the scope of the SingleCellAtlas the authors aim to provide for the community.

**Response:**

Thank you so much for the thoughtful concern. As mentioned in the earlier response, we have now used a mitochondrial content threshold of < 10% to include more cells, i.e., to retain higher number of cell types. We have updated every tissue affected by the mitochondrial threshold and regenerate all phenotypic plots with regards to the change in the threshold. These changes can be viewed on the website under the individual page of each tissue under: [https://www.singlecellatlas.org/sca-atlas](https://and) and database queries under: [https://www.singlecellatlas.org/omics-search.](https://www.singlecellatlas.org/omics-search) We have also updated corresponding in the manuscript, figures, and supplementary tables.

Minor comments:

1. The reviewer is satisfied with this response.

**Response:**

Thank you so much for the suggestions and we will continue to make SCA better.

**Third round of review**

**Reviewer 2**

In their revised version, the authors have made a great effort to improve the manuscript based on this reviewer's suggestions, by for example updating the mitochondrial content threshold, following the recommended best practices. In addition, the authors have modified their multiple testing correction method to standardize it throughout the whole database. Furthermore, missing files have been added to the resource.

However, this reviewer continues to be concerned about the absence of fragment files in the database. Specifically, the reviewer does not understand how the authors could integrate the scATAC-seq datasets without considering the same set of peaks per sample. The genome liftover might not be enough when all samples have been analyzed separately and therefore, no common peaks have been detected.

Concerning the QC, this reviewer has not found the requested plots. Instead, the authors have included the embedding QC which this reviewer doesn’t understand to be useful. This reviewer is also concerned by the comparative database table, as this reviewer deems the presentation to be biased or even wrong. For instance, one can easily check the number of omics included in the Single Cell Portal, which is higher than what is stated.

Please see the related comments below, based on the major points that were raised/remaining in the previous review. This reviewer absolutely considers the author’s manuscript to be worthy of publication but would consider the remaining points to be important to address.

1. The reviewer is concerned by the comparative table the authors have generated, as we think some of the columns are biased or inaccurate. For instance, the Single Cell portal has more data modalities available than the ones mentioned in the table. It would also be important that the authors specify the number of cells per study and the number of cell types in the comparative table. Furthermore, the descriptions used to compare the selected resources continue to be rather subjective as no weaknesses are discussed.

2. The reviewer is satisfied with this response.

3. This reviewer does not understand how the data integration was performed when the FASTQ files were not available. According to best practices, integration in scATAC-seq data should be performed using the same set of peaks. In the future, the direct processing of FASTQ files by a stable pipeline should be considered, especially for scATAC-seq where it could help for data integration and providing missing files.

Furthermore, this reviewer does not understand how it is still possible to lose so many cells after increasing the mitochondrial cut-off and wonders whether the raw cell matrix instead of the filtered cell matrix was used for the calculation of the total amount of cells considered for this database. The authors should clarify this and update the numbers according to the filtered cell matrix in case this was not done previously. In addition, the authors forgot to modify the new mitochondrial threshold in the methods section (page 19).

7.-9. The reviewer is satisfied with this response.
11. The reviewer is satisfied with this response.

**Authors Response**

**Point-by-point responses to the reviewers’ comments:**

**Additional Comments from the Chief Editor:**

As you will see, the report is broadly favorable, so we are interested in publishing the manuscript, however a few issues remain. We feel that these issues must be addressed in full, before we make a firm commitment to publication. When revising your manuscript, please ensure that all the points raised by the referee are addressed. In particular, please elaborate on the scATAC-seq integration and absence of fragment files, clarify and further correct mitochondrial content cut-off, please also further update and discuss the comparative database overview as suggested by the reviewer.

**Response:**

Thank you so much for the feedback and we have made the following changes to the

manuscript and the database accordingly:

- **scATAC-seq Integration and Absence of Fragment Files:**

We have now run the samples with their fragments files available and presented an extensive overview of these samples, in terms of sample-specific (individual samples), and tissue-specific (integrated samples) phenotypes. These extensive and in-depth assessments, including quality control (QC) metrics, can be accessed via:

1. Sample-specific phenotypic queries: <https://www.singlecellatlas.org/scatac-samplewise>
2. Tissue-specific phenotypic queries: <https://www.singlecellatlas.org/scatac-tissuewise-phenotypes>

Through the integration process, we adhere to the recommended steps by the Signac package [1] and the quality of integration has been traced back as shown in **Figure R1**. Moreover, to demonstrate further, we have also compared, across the tissues with fragment files, the outcomes for (i) merged peaks (the pre-integration plots shown under the dropdown list “Pre/Post Integration (Method used by SCA)” under the Quality Control tab in <https://www.singlecellatlas.org/scatac-tissuewise-phenotypes>); (ii) post-integration outcomes based on the methods deployed by Signac [1] (the post-integration plots shown under the dropdown list “Pre/Post Integration (Method used by SCA)” under the Quality Control tab in the above link); (iii) as well as the QC outcome based on common peaks. Of these quality control plots across the tissues, integration procedure has shown to outweigh the effect of merging by common peaks, which is also the other method apart from integration suggested by Signac. We therefore are confident of our analysis outcomes and even though sample files with no fragment files have limited further phenotypic exploratory analyses such as motif enrichment analysis, the process before that, including QC, dimension reduction, clustering, etc, have all closely abided by the steps recommended by Signac.

- **Mitochondrial Content Cut-Off:**

Your feedback on the mitochondrial content cut-off is noted. We have revisited our data, revised our methods, and have now included a summary table (**Table R1**) of the difference in cell count across tissues for both 5% and 10% mitochondrial cut-offs. Please also note that, as other processes, such as removal of doublets using DoubletDecon [2] and further filtering of cells based on their number of unique features, could also contribute to the drainage of low-quality cells. However, raw files are huge and it has been difficult to trace back the intermediate cell count numbers for each of these process.

- **Comparative Database Overview:**

In light of the concerns raised, we have made necessary corrections and double-

checked on the extent of features included for comparison.

Thank you so much once again for summarizing the concerns and suggestions raised by you and reviewer #2.

**Figure R1. UMAP representations of tissues previously run without accompanied fragment files.** Colours indicate sample id.

**Reviewer #2**

In their revised version, the authors have made a great effort to improve the manuscript based on this reviewer's suggestions, by for example updating the mitochondrial content threshold, following the recommended best practices. In addition, the authors have modified their multiple testing correction method to standardize it throughout the whole database. Furthermore, missing files have been added to the resource.

However, this reviewer continues to be concerned about the absence of fragment files in the database. Specifically, the reviewer does not understand how the authors could integrate the scATAC-seq datasets without considering the same set of peaks per sample. The genome liftover might not be enough when all samples have been analyzed separately and therefore, no common peaks have been detected.

Concerning the QC, this reviewer has not found the requested plots. Instead, the authors have included the embedding QC which this reviewer doesn't understand to be useful. This reviewer is also concerned by the comparative database table, as this reviewer deems the presentation to be biased or even wrong. For instance, one can easily check the number of omics included in the Single Cell Portal, which is higher than what is stated.

Please see the related comments below, based on the major points that were raised/remaining in the previous review. This reviewer absolutely considers the author's manuscript to be worthy of publication but would consider the remaining points to be important to address.

**Response:**

Thank you so much for the unwavering support and the faith you have placed in our work. Your insights and guidance have been invaluable in refining our manuscript and we truly appreciate the time and expertise you have dedicated to our work to bring the quality of the database to a greater height. With regards to the concerns you have raised:

- **Concerning scATAC-seq fragment files and data integration:**

We acknowledged your concerns regarding the absence of fragment files and the potential implications on data integration. We recognize the importance of having a unified set of peaks when integrating scATAC-seq datasets. Our earlier approach, while standard in certain contexts, may not be optimal for providing a unified resource that caters to diverse research questions. To address this concern, as mentioned above, we have re-processed the scATAC-seq datasets and conducted extensive analyses for the sample with fragment files, and have also tested the quality control differences between merging based on common peaks, as well as based on integration method following practices by Signac [1]. These outcomes can be found Certainly:

Analyses were run for both sample-specific and tissue-specific phenotypes and is accessible on the database website under:

1. Sample-specific phenotypic queries: <https://www.singlecellatlas.org/scatac-samplewise>
2. Tissue-specific phenotypic queries: <https://www.singlecellatlas.org/scatac-tissuewise-phenotypes>

We have compared outcomes across tissues using fragment files in areas such as merged peaks, post-integration, and QC based on common peaks. The provided links showcase these comparisons. Our results, tested across tissues, indicate the superiority of the integration method over merging by common peaks. An example of which is the adipose tissue as shown in **Figure R2**. For the previous analysis without the fragment files, good integration quality can be seen in the examples provided in **Figure R1**, despite the limitations of processing without fragment files.

With regards to the genome liftover, we have used it in prior due to the differences in the reference genome used by different data source during the alignment step and for finding of overlapping peaks due to the shortage of fragment files. Despite limitations with the fragment files, our analysis, from QC to clustering, aligns with the recommended practices by Signac [1].

**Figure R2. UMAP representations of the adipose tissue run with accompanied fragment files.** Results of the merged by common peaks were compared against merged without considering common peaks (Before Integration) and with the integration procedure. Colours indicate sample id.

- **Concerning the QC plots for scRNA-seq and scATAC-seq:**

We sincerely apologize for any oversight on our part in including the most relevant QC plots. The embedding QC under the Source tab of the database [(https://www.singlecellatlas.org/sources)](https://www.singlecellatlas.org/sources) was included as an additional quality measure for the quality control process (**Figure R3**). If you meant the QC plots for the scATAC-seq, the newest links provided contained a list of QC plots for both samplewise (**Figure R4**) and tissuewise quality control steps (**Figure R5**). We aim to provide transparency in the data processing and quality assessment stages, and these plots are crucial in that aspect.

- **With regards to the comparative database table:**

We have promptly re-examined our comparative table, cross-check with the most updated sources and ensure that the information provided is both accurate and unbiased. We appreciate your vigilance in pointing out these discrepancies. We have stated in asterisks in **Table S1** of the manuscript, that, “Please note that technologies such as SmartSeq2 and 10X Genomics, or protocols such as single-nucleus RNA-seq and single-cell RNA-seq, are all considered under scRNA-seq omics and are counted towards one omics type.”. In any case, thank you for your careful observation, we have missed out the omics, scImmune Profiling, as the protocol list is labelling it with the version number, 10X v5’, in their portal (see **Figure R6**). We have made the necessary changes in **Table S1**.

For CITE-seq, the ability of the technology to profile multimodally, the surface antibodies (proteomics) in addition to its scRNA-sequencing (transcriptomics), we regard it as having two omics (hereby when we talked about the number of omics, we take it as separate omics entities, i.e. two omics are sequenced by CITE-seq).

With regards to the number of omics stated in the manuscript, we do not consider multimodal as one omics type, as they include individual omics type and it will be overly confusing in addition to different technology names.

Thank you for considering our work worthy of publication. We will endeavor to make all necessary changes promptly to meet the standards expected by the journal and its readership.

**Figure R3. Quality control metrics under the Source tab of the database.** QC plots showing the extent of integration for scRNA-seq data.

**Figure R4. User-interface (UI) for scATAC-seq samplewise analysis with fragment files included.** Three main tabs containing extensive visualizations on various parts of the analysis and phenotypic discovery steps.

**Figure R5. UI for scATAC-seq tissuewise analysis with fragment files included.** Three main tabs containing extensive visualizations on various parts of the analysis and phenotypic discovery steps.

"mem ra

a Some 0,110. I'l•••••••••.

***WM. iremin• spin I***

***=ode.*	•••••**

**Roe reeve, ow,**

**wereleomusreveeve•eweemeasc.'"••••4**

**.	.	.
vee•••**

***mow..* 410 1•••••• 1 •**

**.4810.**

***SOW 044 4.01 at• nbp if Ana^,^ MINN, '***

- **PloloR OPoPP14411,•••••••••••••4, ylmomprolm OmmlOworrim...••••••• Oyu *r ••• eldrandlint***

!WY rr. a. ***v....4,4.May... Oa. wi•* meet.**

**Pimpoomi Alm ,mma, *ma* am]**

- **10410**
- ***OM. . SY 444.e^.^* *VW 0•60 P. VIM.. •••••••• POO townie ••••••••••••••• P.I.1141144••••••••4•0 MOO,. O. *WPM* moylmmam..mOmmllyolmaml immylal 1 • •M ••••••••••••••**

**M.o..**

**10.•••**

***OmbOo* PO. DOOM Pol.. *MP* ••••**

- **MollM100•1•0704010••••••• boyfmnyirommold.lemmml1.43. *fom***

**eeeel•-•4^4^44,4^4^44 eleteml meter Ted, IPPON.**

**•••••••••••••••••••=y.W•14n**

**Oro. ••••••••••••••••••••••••••••0**

**•••••. ma.**

***a..r	a env***

**Ueieve.44ee,	*ovalite***

**toeclz *ur,a rae.***

***U***

**Cell**

**r—crs er. A=Arstrq vrq•-adeamrct**

**PORTAL**

**^IIMII^IIIaOSir29Ie Cell**

- **tamet mem a•Seavre**

men •	yen *•*

**Mro.PCI• SY PI pd. ppolaP	P.M.**

**eve pewee n'•**

**•••••**

**moo,**

**fmoOl.**

***Ow* Yam**

**room. moymaimp**

**e	414411fm.• PPM. PIC • WYK 1p**

**Vo•^0^01.•^,^00 wan am, g •mg mew. NI ••••••••••••••••••4•0 IO. •••11••••• ilIMP•momMPLOY • he P. 11•1•0•110% PAYM•62**

- ***k^.^"^.^*	rerenre. 0, OCI••••••• • TOM.. OM**

**OA. 4	weeieereeer**NM

***onl*	pa M. Y./ • &Mem MONO *AAra* NW Po ••••••••**

**WV. ow. mai, •1Immpl 'rem r Pe*.**

Vol.,. lea WO. i

***••••••••*	•••••••	pommomv.**

- **Noe* Ire.fee I }met ”••**

**11.1.1.1•114mimIlmmi**

- **Y•Sa.	1.04.1441.44•1•Pmfairo**

**OMOol <Mk LOY • I* •••	••••0•40**

**••••• w.*r***.ee

**analoopre• Malls mime DWI.. IMMO •••**

**pew elle. areere amommo**

**pg••••••••0 yymmoy IP•mmo	km, ova**

**1.0•00.**

***vermeve* ewe**

_n**omoym yalloo Immo re•Mmy. 3406-yry**

**eve 4444eree•	Ile•MIVeseell**

SIM

•

4

**IPPYIPOMPIP	vex. It**

ova • Ir. segrn.abee •••••• teal

**or***

**eepeeemeepee•	*•••••••••••••* tem.. Ollee ••••• •
lymme••••••••••••**

**UM VI l••••• Yoe**

**Lyra v•per.# eve.^4^4# • he *•Ingeoltie***

S***OY***

***'NI* OOP..**

**omoomommolmvm1mommmi,•••••••,••**

**Woo,* ommillymy MIZ•••••••4•Ymi**

***..••••* PM,**

**POMP MP • MINIM *Moo.a***

4	v.**ONO 44MM Val if••••••	PVIIMYCO, CAN 1011101.3**

- **mar	my.* ,p,p_mo_p_o_.	yyp_m_**
- **,•• worm "epee. twee WeltlelTeel1.#0441•Ve• ••••••••••**

***••••••mis* .m••••Im	ImMaY••••••mo. 'Now.**

- **YimPY**

*elv•VPIIIIIIKO* PPM.

***.4( ,P^,^TIMA***

Cal

| **N. NAY •••*4 oral aloof* •** | **—d MCA.%** |
| --- | --- |

**10••••••• .11 CMCYC/4•41•1•6101•11V**

**CS, *lapowcaa a “Will 1.0* tnr ee4**

**atm**

**eel...neve war •^4^4., .160 Ave,**

**4••• NO	Sas, ealcssirece**

**Maly •••47**

***.4•Nalo • 4..41***

***MAO 0.0. fel MS 1.561,1".141. hit* erernmas rmomol**

**3600 m• ova. MM.. ma. ••• • obit yo. OOP.**

**MOM..**

- **Y..3^.^.1174 • -**

**Figure R6. Omics included in Single Cell Portal.** Filtering list for the library protocols available in the portal. Specific omics-related names are box-out in red.

8

1. The reviewer is concerned by the comparative table the authors have generated, as we think some of the columns are biased or inaccurate. For instance, the Single Cell portal has more data modalities available than the ones mentioned in the table. It would also be important that the authors specify the number of cells per study and the number of cell types in the comparative table. Furthermore, the descriptions used to compare the selected resources continue to be rather subjective as no weaknesses are discussed.

**Response:**

Thank you so much for raising the concern and the careful checks. As mentioned above, we have corrected our comparative table accordingly. With regards to the number of cells per study, this may be difficult as portals such as Single Cell Portal and Human Cell Atlas, are data repositories sites and hundreds and up to thousands of studies were included in these portals (for archiving purposes). In any case, thank you very much for the advice given.

2. The reviewer is satisfied with this response.

**Response:**

Thank you for the positive comment.

3. This reviewer does not understand how the data integration was performed when the FASTQ files were not available. According to best practices, integration in scATAC-seq data should be performed using the same set of peaks. In the future, the direct processing of FASTQ files by a stable pipeline should be considered, especially for scATAC-seq where it could help for data integration and providing missing files.

Furthermore, this reviewer does not understand how it is still possible to lose so many cells after increasing the mitochondrial cut-off and wonders whether the raw cell matrix instead of the filtered cell matrix was used for the calculation of the total amount of cells considered for this database. The authors should clarify this and update the numbers according to the filtered cell matrix in case this was not done previously. In addition, the authors forgot to modify the new mitochondrial threshold in the methods section (page 19).

**Response**

Thank you so much for raising the question. As mentioned above, scATAC-seq data was run based on the steps recommended by Signac, and even though limited steps can be taken with the use of fragment files, procedures which could be done without fragment files were carried out. We have also now included analyses results and visualizations for both samplewise and tissuewise steps for samples with fragment files and are available in the following links:

- <https://www.singlecellatlas.org/scatac-samplewise>
- <https://www.singlecellatlas.org/scatac-tissuewise-phenotypes>

For the number of cells, we have tabulated the cell counts for each tissue in Table R1. As other steps such as the use of DoubletDecon to remove doublets, and filtering based on unique number of features, these steps also contributed to the drop in cell count after filtering. Many thanks for pointing out the mistake on page 19, it has been rectified now.

7.-9. The reviewer is satisfied with this response.

**Response:**

Thank you for the positive comment.

11. The reviewer is satisfied with this response.

**Response:**

Thank you so much for the positive comment.

**References**

1. Stuart T, Srivastava A, Madad S, Lareau CA, Satija R: **Single-cell chromatin state analysis with Signac.** *Nature Methods* 2021, **18:**1333-1341.
2. DePasquale EAK, Schnell DJ, Van Camp P-J, Valiente-Alandí Í, Blaxall BC, Grimes HL, Singh H, Salomonis N: **DoubletDecon: Deconvoluting Doublets from Single-Cell RNA-Sequencing Data.** *Cell Reports* 2019, **29:**1718-1727.e1718.

**Fourth round of review**

**Reviewer 2**

In this last revision, the authors have made an effort to accommodate some of this reviewer's requests, improving the consistency of their methodology and manuscript. As a result, the authors have incorporated quality control plots in their database (including sample-specific and tissue-specific phenotypic queries) and modified the comparative table for accuracy.

However, this reviewer continues to be concerned about some of the authors’ statements. Specifically, this reviewer believes the authors haven’t been able to justify their methodological strategy for scATAC-seq data integration. This remains a central concern and needs to be acknowledged before publishing the manuscript.

A. In general, this Reviewer truly believes the database would greatly benefit from the processing of FASTQ files by an established pipeline, in contrast to the direct use of cell ranger outputs as is the case so far. Yet, this Reviewer also acknowledges the challenge that this would represent for the first release of the SCA database. The authors should however at least make some clarifying statements concerning the next versions. This would constitute several major improvements, such as:
- global reproducibility (e.g., alignment against the same genome version)
- doublet removal for scATAC-seq (and not only scRNA-seq)
- working on complete processed files, enabling among others: the application of standard QC (e.g., TSS enrichment and nucleosome signal for scATAC-seq) and correct integrative analysis for scATAC-seq (see point C)

Those improvements should be implemented in the next release of the database as they correspond to the best practices (compare to Signac vignettes, see also below).

B. This reviewer appreciates the author’s effort in ensuring the accuracy of the comparative table. While agreeing with the general consensus established by the authors, the reviewer still notes some discrepancies concerning the following statement: “With regards to the number of omics stated in the manuscript, we do not consider multimodal as one omics type, as they include individual omics type, and it will be overly confusing in addition to different technology names” (See point E). Still, the reviewer believes this is more of a philosophical discussion and it is satisfied with the overall table.

C. This reviewer is rather confused by the comparison of the “merging” and “integration” methods the authors performed in this revised version of the manuscript. While the integration is optional and aims to correct batch effects, the unification of peaks across different samples is absolutely crucial and has to be performed prior to any integrative analysis, as explained in Signac “scATAC-seq data integration” vignette (https://stuartlab.org/signac/articles/integrate_atac; see text below):

“An important first step in any integrative analysis of single-cell chromatin data is to ensure that the same features are measured in each dataset. Here, we quantify the multiome peaks in the ATAC dataset to ensure that there are common features across the two datasets” See the merge vignette for more information about merging chromatin assays -> https://stuartlab.org/signac/articles/merging

In consequence, the reviewer believes that comparing both methodologies is not possible and, in that sense, concluding that one of the methods is superior to the other is inaccurate. This part should be excluded from both the manuscript and the website. Moreover, this points out the critical importance of having fragment files for each sample and thus the advantage of processing FASTQ files.

D. Regarding the cell numbers, the authors did not clarify whether raw matrices have been used (instead of filtered ones). If this is the case, numerous empty droplets inflate artificially the number of cells processed. Therefore, this reviewer must insist on the update of cell numbers in the manuscript (and website), starting from filtered matrices.

E. Multimodal datasets present obvious advantages, and their use is constantly increasing. As they represent the future of single-cell sequencing technologies, their inclusion in the SCA database, which is presented as the largest multi-omics database, is critical. They should not be reduced to the parallel assessment of several data modalities, instead, they should be considered for their capacity to extract different layers of information from the same cells. For instance, if the ATAC assay of a multiome experiment should indeed be integrated with regular scATAC-seq data, users could be interested and will probably benefit very much from complementing their experiment by including the RNA assay on the same cells.

**Authors Response**

**Point-by-point responses to the reviewers’ comments:**

**Response to the editor and the reviewers**

Reviewer #2: In this last revision, the authors have made an effort to accommodate some of this reviewer's requests, improving the consistency of their methodology and manuscript. As a result, the authors have incorporated quality control plots in their database (including sample-specific and tissue-specific phenotypic queries) and modified the comparative table for accuracy.

However, this reviewer continues to be concerned about some of the authors' statements. Specifically, this reviewer believes the authors haven't been able to justify their methodological strategy for scATAC-seq data integration. This remains a central concern and needs to be acknowledged before publishing the manuscript.

**Response:**

We would like to express our gratitude for your insightful feedback on our manuscript. Your comments have been instrumental in guiding the revisions of our study, particularly concerning the processing of scATAC-seq data. In response to your continued concern about our methodological strategy for scATAC-seq data integration, we have undertaken further extensive revisions to address this critical aspect:

1. **Alignment of Samples to Human Reference Genome hg19:** We have realigned the adult scATAC-seq samples to the human reference genome hg19, aligning with the reference used for the fetal samples.
2. **Rerunning of All Samples with Fragment Files:** To enhance the validity of our approach, all samples were rerun with their respective fragment files. Samples with no fragment files were discarded.
3. **Re-analysis of scATAC-seq Data Based on and Common Peaks and Conducted Integration:** We have completely redone the scATAC-seq data integration based on common peaks. For samples in each tissue, a set of common peaks were produced based on their respective peak bed files, followed by the production of fragment objects and subsequently, samples were then merged and integrated, following the same procedures as shown in:

<https://stuartlab.org/signac/articles/merging>	for	merging	and
<https://stuartlab.org/signac/articles/integrate_atac> for integration.

This revision was undertaken with a clear focus on addressing your concerns regarding the justification of our methodological strategy and other details you have mentioned in the points below. Below, we enclosed point-by-point responses to your comments. We believe that these changes have significantly strengthened the scientific integrity of our work and have addressed the central concern you raised. We hope that these changes meet your expectations and address the concerns you have raised. Thank you for your valuable feedback and for guiding us in improving our manuscript. We look forward to your response and are hopeful for a positive outcome. Please note that, in order to assess the website, pages such as DOWNLOAD tab is password-protected. Every page on the [www.singlecellatlas.org](http://www.singlecellatlas.org) platform that requires a password uses the same credential: “***forgbreview***”.

A. In general, this Reviewer truly believes the database would greatly benefit from the processing of FASTQ files by an established pipeline, in contrast to the direct use of cell ranger outputs as is the case so far. Yet, this Reviewer also acknowledges the challenge that this would represent for the first release of the SCA database. The authors should however at least make some clarifying statements concerning the next versions. This would constitute several major improvements, such as

-	global reproducibility (e.g., alignment against the same genome version)

-	doublet removal for scATAC-seq (and not only scRNA-seq)

-	working on complete processed files, enabling among others: the application of standard QC (e.g., TSS enrichment and nucleosome signal for scATAC-seq) and correct integrative analysis for scATAC-seq (see point C)

Those improvements should be implemented in the next release of the database as they correspond to the best practices (cf Signac vignettes).

**Response:**

Thank you for your insightful recommendations. We fully concur with your suggestions and have accordingly rerun the scATAC-seq data for adult samples using raw fastq files, aligning these to the human genome reference hg19, in line with the fetal samples. We have also reprocessed all samples with their respective fragment files, rerun the analysis based on common peaks between samples of the same tissue, in order to carry out correct integrative analysis for scATAC-seq data. In response to your suggestion, we have updated our website with details about future versions, which you can find at: [https://www.singlecellatlas.org/news.](https://This) This includes major enhancements suggested by you, apart from those which were already implemented in this current revision. We are committed to continual improvement and have scheduled our next release for summer 2024, which will also include the latest data from 2023.

B. This reviewer appreciates the author's effort in ensuring the accuracy of the comparative table. While agreeing with the general consensus established by the authors, the reviewer still notes some discrepancies concerning the following statement: "With regards to the number of omics stated in the manuscript, we do not consider multimodal as one omics type, as they include individual omics type, and it will be overly confusing in addition to different technology names" (See point E). Still, the reviewer believes this is more of a philosophical discussion and it is satisfied with the overall table.

**Response:**

Thank you for your valuable feedback and insights. We wholeheartedly agree that multimodal analysis is essential in this era of multiomics discovery. In our current database, we have incorporated multimodal findings that combine scImmune Profiling with scRNA-seq data. While we recognize the importance of multiome scATAC-seq, we are also mindful of the current limitations in the volume of available data for this modality. We are actively collecting published datasets in this area and plan to release them once we have amassed a more substantial number of samples across various tissues for multiome scATAC-seq analysis.

C. This reviewer is rather confused by the comparison of the "merging" and "integration" methods the authors performed in this revised version of the manuscript. While the integration is optional and aims to correct batch effects, the unification of peaks across different samples is absolutely crucial and has to be performed prior to any integrative analysis, as explained in Signac "scATAC-seq data integration" vignette [(https://eur01.safelinks.protection.outlook.com/?url=https%3A%2F%2Fstuartlab.org%2Fsign](https://eur01.safelinks.protection.outlook.com/?url=https%3A%2F%2Fstuartlab.org%2Fsignac%2Farticles%2Fintegrate_atac&data=05%7C01%7Cxuexin.li%40ki.se) [ac%2Farticles%2Fintegrate](https://eur01.safelinks.protection.outlook.com/?url=https%3A%2F%2Fstuartlab.org%2Fsignac%2Farticles%2Fintegrate_atac&data=05%7C01%7Cxuexin.li%40ki.se)_[atac&data=05%7C01%7Cxuexin.li%40ki.se](https://eur01.safelinks.protection.outlook.com/?url=https%3A%2F%2Fstuartlab.org%2Fsignac%2Farticles%2Fintegrate_atac&data=05%7C01%7Cxuexin.li%40ki.se) %7C0bbf673d5fb44 e5344e808dbdc5ff016%7Cbff7eef1cf4b4f32be3da1dda043c05d%7C0%7C0%7C638346076 837249312%7CUnknown%7CTWFpbGZsb3d8eyJWIjoiMC4wLjAwMDAiLCJQIjoiV2luMzIiLC JBTiI6Ik1haWwiLCJXVCI6Mn0%3D%7C3000%7C%7C%7C&sdata=%2B%2BglAnOThVMC RyTIabNRpOGc52H1kGmeFaZpOWEbMpQ%3D&reserved=0; see text below):

"An important first step in any integrative analysis of single-cell chromatin data is to ensure that the same features are measured in each dataset. Here, we quantify the multiome peaks in the ATAC dataset to ensure that there are common features across the two datasets" See the merge vignette for more information about merging chromatin assays -> [https://eur01.safelinks.protection.outlook.com/?url=https%3A%2F%2Fstuartlab.org%2Fsig](https://stuartlab.org/signac/articles/merging) [nac%2Farticles%2Fmerging&data=05%7C01%7Cxuexin.li%40ki.se%7C0bbf673d5fb44e534](https://stuartlab.org/signac/articles/merging) [4e808dbdc5ff016%7Cbff7eef1cf4b4f32be3da1dda043c05d%7C0%7C0%7C6383460768372](https://stuartlab.org/signac/articles/merging) [49312%7CUnknown%7CTWFpbGZsb3d8eyJWIjoiMC4wLjAwMDAiLCJQIjoiV2luMzIiLCJBTiI](https://stuartlab.org/signac/articles/merging) [6Ik1haWwiLCJXVCI6Mn0%3D%7C3000%7C%7C%7C&sdata=gAVGH9dsOC35JpsoIQQjF](https://stuartlab.org/signac/articles/merging) [9mUq%2FUG%2BoeUmpDW05FAGeY%3D&reserved=0](https://stuartlab.org/signac/articles/merging)

In consequence, the reviewer believes that comparing both methodologies is not possible and, in that sense, concluding that one of the methods is superior to the other is inaccurate. This part should be excluded from both the manuscript and the website. Moreover, this points out the critical importance of having fragment files for each sample and thus the advantage of processing FASTQ files.

**Response:**

Thank you so much for highlighting this issue and described in high detail. We have now removed the comparison of the two methods from both our manuscript and website. To ensure the integrity and consistency of our data, we have meticulously rerun all scATAC-seq samples, especially for adult scATAC-seq samples, which were rerun from fastq files to realign to human genome hg19, which was used by the fetal samples. Specifically, we have processed each sample with its corresponding fragment files, discarding any samples lacking these files. Our approach for the scATAC-seq samples in each tissue included the unification of peaks, ensuring that all samples shared common peaks, followed by the merging and integration of samples for each tissue based on the steps indicated in the links you have provided. The key steps taken are as follows:

1. **Alignment to Human Reference Genome hg19:** Adult scATAC-seq samples were realigned to the human genome reference hg19, so as to be consistent with the fetal data reference.
2. **Rerunning with Fragment Files:** All samples were rerun with their respective fragment files, and samples without fragment files were excluded.
3. **Re-analysis Based on Common Peaks and Integration:** The scATAC-seq data integration was completely redone based on common peaks. For each tissue, a unified set of common peaks was identified from sample peak bed files, leading to the merging and integration of samples. This process aligns with the protocols outlined at

<https://stuartlab.org/signac/articles/merging>	for	merging	and	for
[https://stuartlab.org/signac/articles/integrate_atac.](https://stuartlab.org/signac/articles/integrate_atac)

The results of all rerun analyses are accessible in four new applications located under the QUERY tab (refer to **Figure R1**)

**Figure R1.** Screenshot of the four additional query applications for scATAC-seq under the QUERY tab on the SCA platform.

D. Regarding the cell numbers, the authors did not clarify whether raw matrices have been used (instead of filtered ones). If this is the case, numerous empty droplets inflate artificially the number of cells processed. Therefore, this reviewer must insist on the update of cell numbers in the manuscript (and website), starting from filtered matrices.

**Response:**

We regret any confusion our previous terminology may have caused. In our initial manuscript, we used the term “raw gene counts” to indicate unnormalized gene counts, rather than referring exclusively to the raw_feature_matrix file in a 10X Genomics Cellranger output, as some samples were processed using technologies other than 10X Genomics. To clarify in this revision, we have replaced “raw gene counts” with “raw read counts (produced after quantification)” Specifically for 10X Genomics outputs, we have now clearly stated that we utilized the filtered_feature_matrix file, not the raw_feature_matrix file, as detailed in the Methods section under ‘Data source and data retrieval’.

E. Multimodal datasets present obvious advantages, and their use is constantly increasing. As they represent the future of single-cell sequencing technologies, their inclusion in the SCA database, which is presented as the largest multi-omics database, is critical. They should not be reduced to the parallel assessment of several data modalities, instead, they should be considered for their capacity to extract different layers of information from the same cells. For instance, if the ATAC assay of a multiome experiment should indeed be integrated with regular scATAC-seq data, users could be interested and will probably benefit very much from complementing their experiment by including the RNA assay on the same cells.

**Response:**

Thank you for your valuable feedback. We have dedicated a section on our website [(https://www.singlecellatlas.org/scimmune-scrnaseq-multitissues](https://www.singlecellatlas.org/scimmune-scrnaseq-multitissues)) specifically for showcasing the multimodal phenotypic discoveries from scRNA-seq and scImmune profiling. Expanding this multimodality beyond these two omics, as you suggested, to integrate ATAC assay data from multiome experiments with regular scATAC-seq data, is indeed a compelling direction. Although we currently possess some data with these characteristics, their volume is relatively limited. We plan to gather more samples to enhance the statistical power and will aim to include these enriched multimodal results in our upcoming release.

**Fifth round of review**

**Reviewer 2**

In this latest revised version, the authors have tried to accommodate the remaining requests, but some concerns unfortunately remain. Therefore, this reviewer thinks there are unresolved issues that are important to clarify before publication. Although this reviewer very much agrees that there should be a “balance between perfection and practicality”, this reviewer would like to emphasize that this balance should never be in dispute with good and very reasonable scientific practices. This is especially crucial for an atlas database aiming at providing “a one-stop, comprehensive, and time-effective resource” for researchers. Specifically, this reviewer thinks the authors must clarify point D as their answer to this date remains unclear.

A. The authors made a substantial effort to re-analyze all scATAC-seq data from FASTQ processing to peak integration. Overall, this reviewer is satisfied with the integrative analysis. Unfortunately, the authors did not use this opportunity to introduce the steps mentioned in the previous revisions related to their analysis, including standard scATAC-seq quality control and doublet removal. Furthermore, this reviewer would recommend the authors align their data to the current reference genome (GRCh38) in future database releases.

B. This reviewer is satisfied with the answer.

C. This reviewer is satisfied with the answer.

D. The answer to this point remains unclear. In the previous rounds of manuscript revision, this reviewer asked for clarification regarding the use of the raw or filtered matrix from cellranger output as a basis for their analysis, justifying the cell numbers before quality control. This is completely unrelated to the gene count normalization since both raw/filtered matrices report raw (i.e., unnormalized) gene counts. The authors claim to use the filtered matrix (which is the standard procedure). However, this reviewer has doubts about the veracity of this affirmation. For instance, according to Table S8, more than 40 million cells of the scRNA-seq dataset (i.e., about 60%) stem from one single study on tonsils. This study (https://pubmed.ncbi.nlm.nih.gov/34623901/) does not report such a high number of cells but rather around 33000 newly generated cells, which is also more in the range of standard 10x-based techniques. Moreover, its respective GEO (GSE165860) repository only provides raw matrices. Therefore, this reviewer would like to reiterate its request and ask the authors to carefully check whether the filtered matrices have been used consistently and update the number of cells collected in the text and on the website.

This is not simply a matter of refinement, as this can be very misleading to future users and as such is not in line with best practices.

E. This reviewer is satisfied with the answer.

**Authors Response**

**Point-by-point responses to the reviewers’ comments:**

**Response to reviewers**

Reviewer #2: In this latest revised version, the authors have tried to accommodate the remaining requests, but some concerns unfortunately remain. Therefore, this reviewer thinks there are unresolved issues that are important to clarify before publication. Although this reviewer very much agrees that there should be a “balance between perfection and practicality”, this reviewer would like to emphasize that this balance should never be in dispute with good and very reasonable scientific practices. This is especially crucial for an atlas database aiming at providing “a one-stop, comprehensive, and time-effective resource” for researchers. Specifically, this reviewer thinks the authors must clarify point D as their answer to this date remains unclear.

**Response:**

Thank you for your feedback and your efforts in persistently refining the database. We have now made the necessary changes to your comments, including the addition of detailed workflow for scATAC-seq data (which is now **Supplementary Figure S8**), and have made further clarification with point D, through comprehensive assessments on the cell counts in the manuscript (as stated in **Supplementary Table S8**). Any data retrieved from published studies which are raw matrices were further filtered based on the same filtering method used by CellRanger. All cell counts stated as Initial_Cell_Count_Filtered_Matrix in **Table S8** now are indeed the cell counts from the filtered matrices and no longer contain cells from the raw matrices.

A. The authors made a substantial effort to re-analyze all scATAC-seq data from FASTQ processing to peak integration. Overall, this reviewer is satisfied with the integrative analysis. Unfortunately, the authors did not use this opportunity to introduce the steps mentioned in the previous revisions related to their analysis, including standard scATAC-seq quality control and doublet removal. Furthermore, this reviewer would recommend the authors align their data to the current reference genome (GRCh38) in future database releases.

**Response:**

Thank you for your comments. Regarding the steps mentioned in previous revisions related to our analysis, we want to clarify that descriptions were indeed provided under the Methods section, specifically under the "ScATAC-Sequencing" sub-headings within the sub-sections "Data processing and assessment of data quality" and "Data integration with batch correction". Nonetheless, we have now enclosed a detailed workflow illustrating the steps taken for scATAC-seq analysis as shown in **Figure R1**. This figure is now cited and further described under the sub-headings “ScATAC-sequencing” in the Methods section and is now included as **Supplementary Figure S8**.

Regarding the alignment of our data to the reference genome version GRCh38 in future releases, we understand your preference for clarity in specifying the genome version. In your previous comment in the previous revision, you recommended to conduct “alignment against the same genome version**”** without specifying GRCh38. Consequently, in our previous improvements, we aligned our samples to the same genome version, which is hg19. Many published studies or databases are still using hg19 or are moving towards GRCh38, and there is an enormous effort to switch to GRCh38 for our project too. Moving forward, we acknowledge your recommendation to align our data to the current reference genome (GRCh38) in future database releases. We will align our data to both hg19 and GRCh38 versions in parallel and separately for users to interpret based on the reference genome version they used for their projects in future releases.

D. The answer to this point remains unclear. In the previous rounds of manuscript revision, this reviewer asked for clarification regarding the use of the raw or filtered matrix from cellranger output as a basis for their analysis, justifying the cell numbers before quality control. This is completely unrelated to the gene count normalization since both raw/filtered matrices report raw (i.e., unnormalized) gene counts. The authors claim to use the filtered matrix (which is the standard procedure). However, this reviewer has doubts about the veracity of this affirmation. For instance, according to Table S8, more than 40 million cells of the scRNA-seq dataset (i.e., about 60%)

stem	from	one	single	study	on	tonsils.	This	study
(<https://eur01.safelinks.protection.outlook.com/?url=https%3A%2F%2Fpubmed.ncbi>. [nlm.nih.gov](http://nlm.nih.gov)%2F34623901%2F&data=05%7C02%[7Cxuexin.li](http://7Cxuexin.li)%[40ki.se](http://40ki.se)%7C6ee4009b cff244ae81aa08dc177ee2ca%7Cbff7eef1cf4b4f32be3da1dda043c05d%7C0%7C0% 7C638411080941768372%7CUnknown%7CTWFpbGZsb3d8eyJWIjoiMC4wLjAwMD AiLCJQIjoiV2luMzIiLCJBTiI6Ik1haWwiLCJXVCI6Mn0%3D%7C3000%7C%7C%7C& sdata=m%2FfO%2FUdqcnSyPHBiJ%2Bk2xu3wjialAgWZN%2FDh9p3R0u0%3D&res erved=0) does not report such a high number of cells but rather around 33000 newly generated cells, which is also more in the range of standard 10x-based techniques. Moreover, its respective GEO (GSE165860) repository only provides raw matrices. Therefore, this reviewer would like to reiterate its request and ask the authors to carefully check whether the filtered matrices have been used consistently and update the number of cells collected in the text and on the website.

This is not simply a matter of refinement, as this can be very misleading to future users and as such is not in line with best practices.

**Response:**

Thank you for your comments. For samples retrieved from studies which are raw matrices, we have now filtered based on the inflection point of the rank plot curve for each of these samples, to obtain a filtered matrix similar to 10X Genomics output (see example of a tonsil sample from GSE165860 in **Figure R2**). We have now retabulated cell counts for filtered matrices and made necessary changes to **Supplementary Table S8**, the manuscript, and the website for the cell counts affected. The cell counts referenced as Initial_Cell_Count_Filtered_Matrix in **Supplementary Table S8** now exclusively represents counts from filtered matrices, excluding any cells from raw matrices. In the revised manuscript, these parts are highlighted in red, especially on Page 3.

4

**Figure R2. Rank plot of the raw matrix of a tonsil sample GSM5051493 from GSE165860.** Filtering of the raw matrix was done based on the inflection point of the rank curve. Cells which are identified as background (colored in grey) are removed from the matrix to form a filtered matrix similar to the filtered matrix output from CellRanger.
